# Visible‐Light Driven Control Over Triply and Quadruply Hydrogen‐Bonded Supramolecular Assemblies

**DOI:** 10.1002/chem.202304033

**Published:** 2024-02-19

**Authors:** Eleanor M. Hilton, Michael A. Jinks, Andrew D. Burnett, Nicholas J. Warren, Andrew J. Wilson

**Affiliations:** ^1^ School of Chemistry University of Leeds Woodhouse Lane Leeds LS2 9JT UK; ^2^ School of Chemical and Process Engineering University of Leeds Woodhouse Lane Leeds LS2 9JT UK; ^3^ School of Chemistry University of Birmingham, Edgbaston Birmingham B15 2TT UK; ^4^ Astbury Centre for Structural Molecular Biology University of Leeds Woodhouse Lane Leeds LS2 9JT UK

**Keywords:** foldamers, hydrogen-bonding motifs, photoswitches, supramolecular polymers •

## Abstract

Supramolecular polymers offer tremendous potential to produce new “smart” materials, however, there remains a need to develop systems that are responsive to external stimuli. In this work, visible‐light responsive hydrogen‐bonded supramolecular polymers comprising photoresponsive supramolecular synthons (**I–III**) consisting of two hydrogen bonding motifs (HBMs) connected by a central ortho‐tetrafluorinated azobenzene have been characterized by DOSY NMR and viscometry. Comparison of different hydrogen‐bonding motifs reveals that assembly in the low and high concentration regimes is strongly influenced by the strength of association between the HBMs. **I**, Incorporating a triply hydrogen‐bonded heterodimer, was found to exhibit concentration dependent switching between a monomeric pseudo‐cycle and supramolecular oligomer through intermolecular hydrogen bonding interactions between the HBMs. **II**, Based on the same photoresponsive scaffold, and incorporating a quadruply hydrogen‐bonded homodimer was found to form a supramolecular polymer which was dependent upon the ring‐chain equilibrium and thus dependent upon both concentration and photochemical stimulus. Finally, **III**, incorporating a quadruply hydrogen‐bonded heterodimer represents the first photoswitchable *AB* type hydrogen‐bonded supramolecular polymer. Depending on the concentration and photostationary state, four different assemblies dominate for both monomers **II** and **III**, demonstrating the ability to control supramolecular assembly and physical properties triggered by light.

## Graphical abstract

Foldamers consisting of two hydrogen bonding motifs (HBMs) connected by a central azobenzene exhibit concentration and light dependent switching between four states.

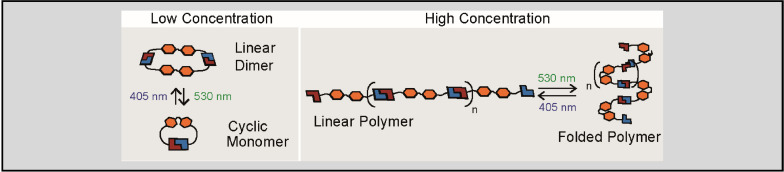

## Introduction

Supramolecular polymers have transformed materials chemistry.[^1^, ^2^, ^3^, ^4^] Such polymers offer tremendous potential as stimuli responsive materials, due to the dynamic nature of the non‐covalent interactions that hold them together; supramolecular materials exhibit properties that include self‐healing, shape memory, and actuation.[^5^, ^6^, ^7^, ^8^, ^9^] Moreover, supramolecular polymers have been used for a range of applications including adhesion, inkjet printing, tissue engineering and drug delivery.[^10^, ^11^, ^12^, ^13^, ^14^] Temperature, pH, redox control and light have all been harnessed to regulate supramolecular polymer assembly,[^15^, ^16^, ^17^, ^18^, ^19^] however, there remains a need to develop systems that are responsive to external stimuli. Foldamers could be used to achieve this; foldamers have been defined as: (i) oligomers or polymers that adopt well defined biomimetic and abiotic secondary, tertiary and quaternary structures; (ii) oligomers that fold into a conformationally ordered state in solution, the structures of which are stabilized by a collection of noncovalent interactions between nonadjacent monomer units.[^20^, ^21^] Foldamers have been used to recognize small molecules or biomacromolecules.[^22^, ^23^, ^24^, ^25^, ^26^, ^27^, ^28^] Regulation of foldamer function through switching is feasible where folding is driven by co‐operative non‐covalent interactions; several stimuli have been used to switch between unfolded and folded forms^[29]^ including: acid/base,^[30]^ cations,^[31]^ anions,[^32^, ^33^] redox state^[34]^ and light.^[35]^ Whilst single chain folded polymers have been reported,[^36^, ^37^] the development of self‐assembled foldamers is less explored.[^31^, ^38^, ^39^, ^40^, ^41^]

Hydrogen bonding motifs (HBMs) have been widely used for supramolecular polymer assembly;[^42^, ^43^, ^44^, ^45^, ^46^, ^47^, ^48^, ^49^] hydrogen‐bonds are reversible, directional, and, when combined into HBMs have tuneable association/disassociation affinities.[^44^, ^50^, ^51^, ^52^, ^53^] A number of light responsive hydrogen‐bond assembled supramolecular polymers have been described.[^54^, ^55^, ^56^, ^57^] Notably, stilbene, dithienylethene and azobenzene photoswitches have been used to regulate assembly of self‐complementary hydrogen‐bond assembled supramolecular polymers (i. e. *AA* type).[^58^, ^59^, ^60^, ^61^, ^62^, ^63^] In general, for ditopic ureidopyrimidinone (UPy)^[64]^ monomers, the concentration dependent ring‐chain equilibria[^65^, ^66^, ^67^] has been shown to bias assembly in favor of lower molecular weight cyclic oligomers for the *cis* or closed form whilst the *trans* or open form is biased towards chain extended polymers. There are few reports on visible light responsive hydrogen‐bond assembled supramolecular polymers,^[61]^ and similarly, no examples of light responsive supramolecular polymers assembled via heterodimerization (i. e. *AA*+*BB* or *AB* type).

Herein, we describe a series of supramolecular synthons that comprise ditopic azobenzene linked HBMs (**I**–**III**), specifically triply hydrogen‐bonded pyridylurea ⋅ amidonaphthyridine (Pyr ⋅ NAP) heterodimers, quadruply hydrogen‐bonded ureidopyrimidinone (UPy ⋅ UPy) homodimers and quadruply hydrogen‐bonded ureidopyrimidinone ⋅ diamidonaphthyridine UPy ⋅ DAN) heterodimers. We show that the folded architecture plays a central role in defining the visible‐light‐responsive supramolecular assembly which is obtained. Whilst the description of these supramolecular synthons as foldamers may be considered imprecise according to the Gellman or Moore definitions[^20^, ^21^] – foldamers generally consist of repeating recognition units – Opie and co‐workers who originally reported **I** ^[68]^ described it as such, whilst in the self‐assembled state, the properties of **I**–**III** all depend on the configuration (and thus conformation) of the monomer which infers interaction with non‐adjacent monomers, thus we use the term self‐assembled foldamer here. In each case, the *cis* (*E*) and *trans* (*Z*) photostationary states are biased toward cyclic or extended conformations respectively and this leads to different discrete hydrogen‐bonded rings and supramolecular polymer assemblies dependent on concentration and hydrogen‐bond dimerization affinity.

## Results and Discussion

**Figure 1:**
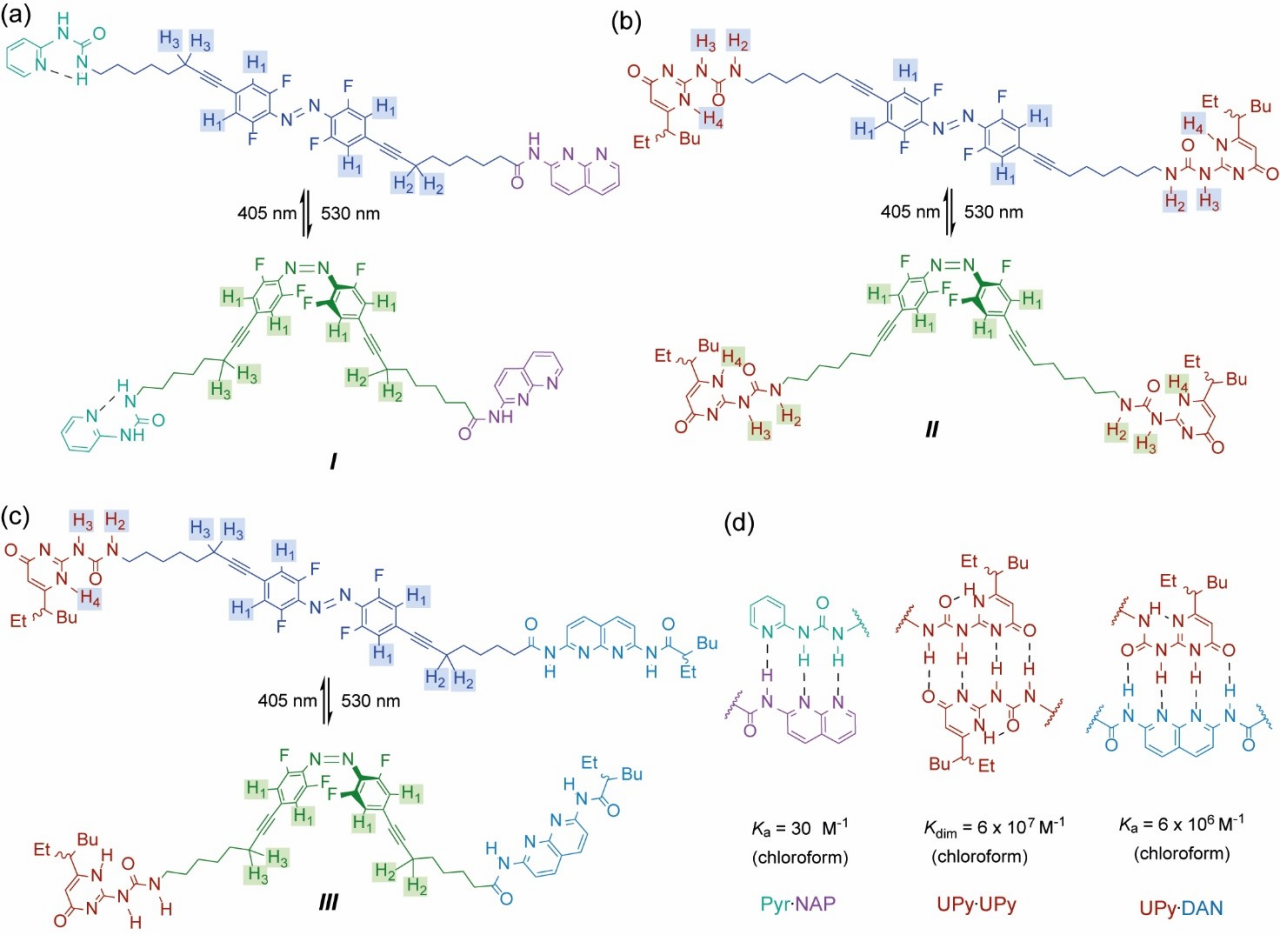
(a) Pyr ⋅ NAP monomer **I** and its *E* (blue) and *Z* (green) isomers; (b) UPy ⋅ UPy monomer **II** and its *E* (blue) and *Z* (green) isomers; (c) UPy ⋅ DAN monomer **III** and its *E* (blue) and *Z* (green) isomers;

We sought to use light as a stimuli to regulate conformation and therefore assembly due to its ability to induce a fast response and remote activation/control without recourse to the addition of further reagents.^[19]^ We selected azobenzenes; commonly used photoswitches that benefit from the ability to switch rapidly and without photofatigue.[^69^, ^70^, ^71^, ^72^, ^73^] Whilst unsubstituted azobenzenes are effectively photoisomerized under UV light, *o*‐tetrafluorinated azobenzenes are effectively isomerized using visible light. In addition, *o*‐tetrafluorinated azobenzenes have been shown to exhibit long half‐lives (over 2 years) in the less thermodynamically favorable *Z* isomer and are synthetically accessible.^,[74,75]^ A molecule incorporating a visible light activated photoswitch and two complementary hydrogen bonding motifs would provide the key elements of a stimuli responsive synthon capable of forming a self‐assembled foldamer. Opie and co‐workers recently described such a scaffold **I** (Figure 1a),^[68]^ comprising a central *o*‐tetrafluorinated azobenzene moiety appended with pyridylurea (Pyr) and amidonaphthyridine (NAP) triple HBMs. These HBMs were shown to associate intramolecularly when the azobenzene adopts the *Z* configuration but could not engage in intermolecular hydrogen‐bonding with the azobenzene in the *E* configuration.^[68]^ The asymmetry of the hydrogen‐bonded heterodimerization enforced a handedness to the folding which could be biased through introduction of stereogenic centers on the backbone, however whilst the rate of photoswitching at different concentrations was assessed, concentration dependent oligomerization was not described in this prior report.

Due to the relatively low Pyr ⋅ NAP dimerization affinity (*K*_a_ ~30 M^−1^, Figure 1d),^[76]^ we considered the possibility that formation of a supramolecular polymer from synthon **I** may be unlikely. Therefore, a second supramolecular synthon, based on the same photoresponsive scaffold (Figure 1b), was designed. **II** Contained two uriedopyrmidinone (UPy), quadruple hydrogen bonding motifs. The dimerization constant for this interaction has been established to be at least *K*_dim_ ~6x10^7^ M^−1^ (Figure 1d).[^64^, ^77^, ^78^] **III** Contained UPy and diamidonaphthyridine (DAN) motifs (Figure 1c); the Upy ⋅ DAN dimerization affinity (*K*_a_ ~6x10^6^ M^−1^ in chloroform, Figure 1d)[^77^, ^79^] is favored over UPy homodimerization by a 20 : 1 ratio and we considered this promising as a supramolecular synthon to assemble the first visible light responsive *AB* type hydrogen‐bonded supramolecular polymer. In this initial study, an *AB* type rather than *AA*+*BB* type system was prioritized given the later presents the additional challenge of stoichiometry control, whilst the former represents a single component system that could be readily compared with **I** and **II**. Syntheses of **I–III** including notes on different routes we explored together with experimental procedures and characterization for final compounds and all intermediates are described in the supporting information.

### Photoswitching

**Figure 2:**
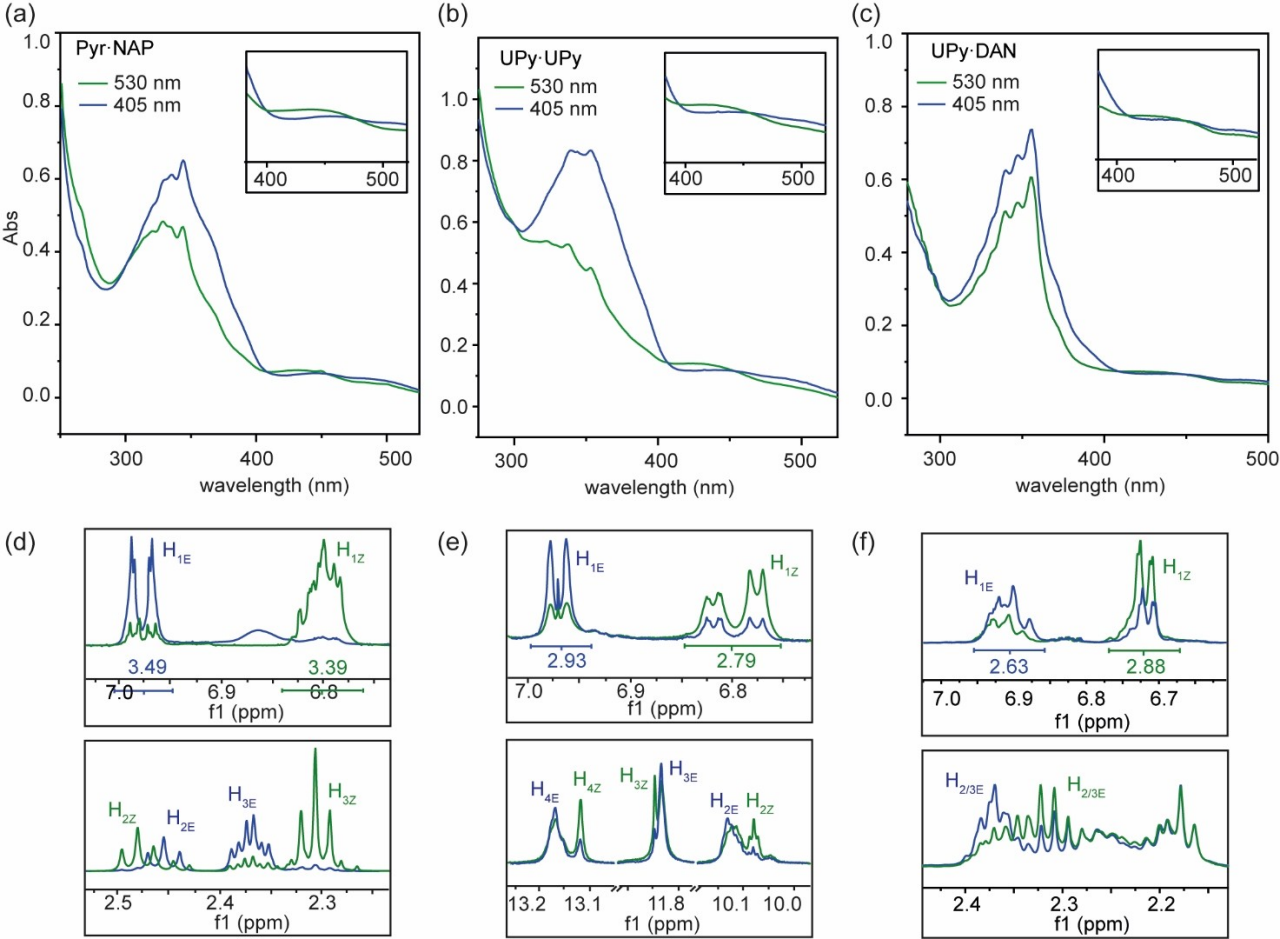
(a) UV‐Vis spectra of **I** in (chloroform, 0.01 mM) after irradiation at 405 nm (blue) 530 nm (green), enlarged inset indicates the n→π* transition within the visible range; (b) UV‐Vis spectra of **II** in (chloroform, 0.01 mM) after irradiation at 405 nm (blue) 530 nm (green), Enlarged inset indicates the n→π* transition within the visible range; (c) UV‐Vis spectra of **III** in (chloroform, 0.01 mM) after irradiation at 405 nm (blue) 530 nm (green), Enlarged inset indicates the n→π* transition within the visible range; (d) **I** ^1^H NMR (500 MHz, CDCl_3_, 298 K, 4 mM) signals showing evidence of switching between isomers after irradiation (Integrals used to calculate photostationary state (PSS) ratios); (e) **II** ^1^H NMR (500 MHz, CDCl_3_, 298 K, 4 mM) signals showing evidence of switching between isomers after irradiation (integrals used to calculate PSS ratios); (f) **III** ^1^H NMR (500 MHz, CDCl_3_, 298 K, 4 mM) signals showing evidence of switching between isomers after irradiation (integrals used to calculate PSS ratios).

To assess photoswitching of **I–III** we used an LED irradiation platform (see supporting information) and initially characterized the photoisomerization behavior using UV‐Vis absorption spectroscopy. In accordance with previous studies on compound **I**,^[68]^ we irradiated the samples in the visible range at 405 nm and 530 nm to exploit the n→π* absorption bands of the photoresponsive azobenzene moiety. The UV‐Vis spectra were recorded after irradiation of 0.01 mM samples for 10 minutes. Blue light (405 nm) was used to produce the *E* isomer of each compound **I–III** and green light (530 nm) was used to generate the *Z* isomer. Upon exposing blue light irradiated samples to green light, the absorption maxima underwent a hypsochromic shift. Upon re‐irradiation with blue light, back‐switching was achieved as evidenced by the corresponding bathochromic shift of the absorption maxima (Figure 2a–c). For ^1^H NMR studies, solutions were prepared in anhydrous deuterochloroform (4 mM) and irradiated with blue light (405 nm) for 10 min and green light (530 nm) for 10 min. ^1^H NMR spectra showed evidence of switching *via* a change of environment for key proton resonances (Figure 2d–f). The ratio of *E*/*Z* isomers could be estimated from the integrals of the azobenzene protons, which show characteristic changes in chemical shift (δ) after irradiation. The distribution at the photo‐stationary state for compound **I** was found to be 13 : 87 *Z* : *E* after irradiation at 405 nm and 85 : 15 *Z : E* after irradiation at 530 nm, similar to the previously reported values.^[68]^ For compound **II** the distribution at the photo‐stationary state after irradiation at 405 nm was 27 : 73 *Z : E* and 71 : 29 *Z : E* after irradiation at 530 nm. For compound **III** the distribution at the photo‐stationary after irradiation at 405 nm was 34 : 66 *Z : E* and 72 : 28 *Z : E* after irradiation at 530 nm). These results indicate that stronger hydrogen‐bonding may suppress photoswitching.

### Analyses of Supramolecular Polymer Assembly

After characterizing their photoswitching capabilities, the propensity for **I–III** to form supramolecular polymers was investigated. The dynamics of hydrogen‐bonding (i. e. reversibility) mean that reorganization would likely occur during size exclusion chromatography and so GPC is less appropriate for supramolecular assembly characterization in this instance, hence we initially used ^1^H DOSY NMR. Samples of **I** were prepared at 4 mM (deuterochloroform); at this concentration a small difference in diffusion coefficient (D) was observed upon photoswitching (as evidenced from the ^1^H NMR). When converted to molecular weight (M_w_, *vide infra*), these D values indicate the size of the *E−* **I** and *Z−* **I** species is similar and that the respective photostationary states are likely monomeric. In contrast at 4 mM, **II** exhibited a larger difference in diffusion coefficient (D) between the *E* and *Z* form suggesting a significant difference in molecular weight (which we attribute to formation of cyclic dimers *vide infra*). A similar but less pronounced difference in D was observed for **III**.

**Figure 3:**
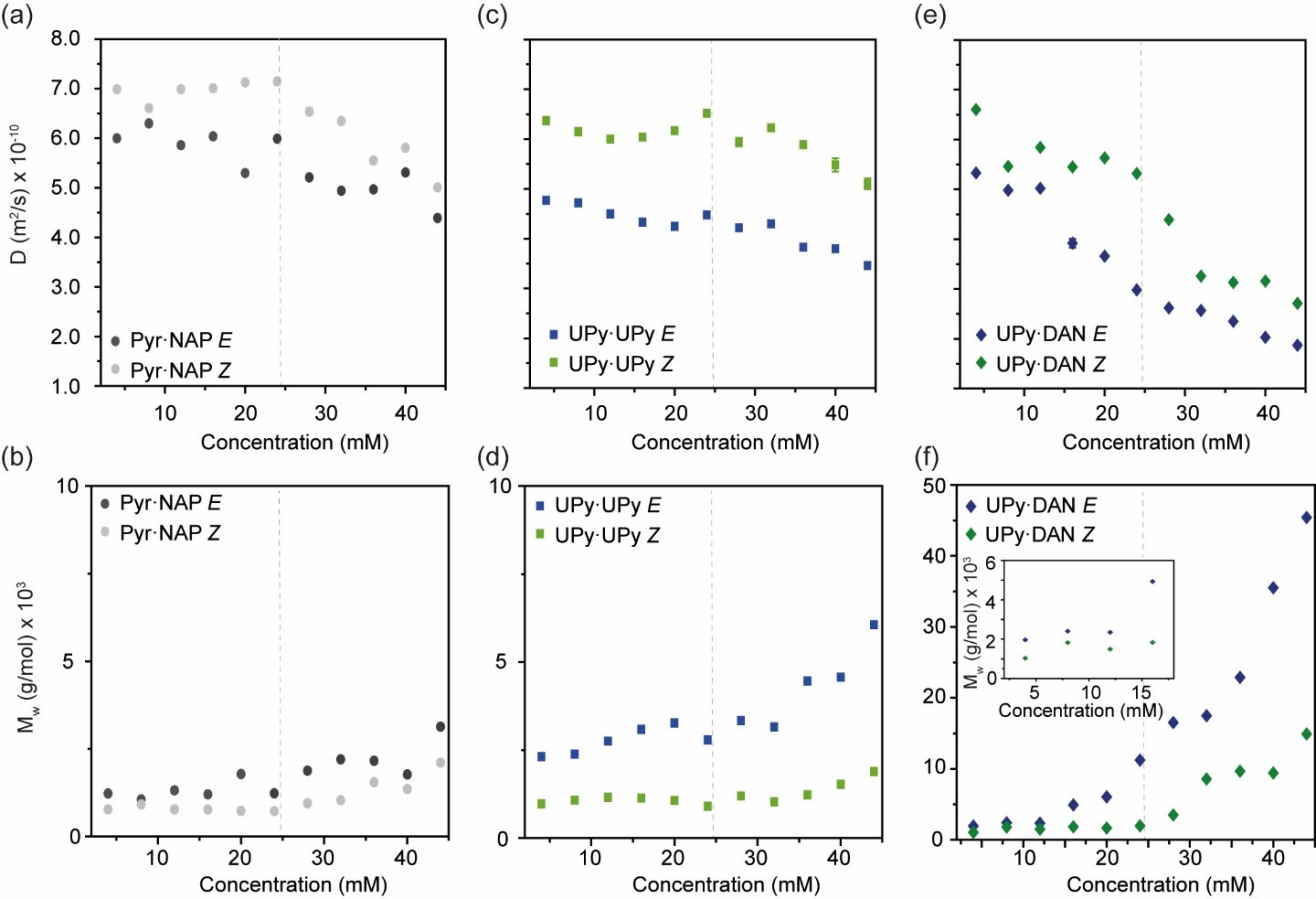
(a) Concentration dependent diffusion coefficient of **I** (Pyr ⋅ NAP *E*, dark grey and Pyr ⋅ NAP *Z*, light grey); (b) variation with concentration of approximate molecular weight of each isomer of **I**; (c) concentration dependent diffusion coefficient of **II** (UPy ⋅ UPy *Z*, light green and UPy ⋅ UPy *E*, blue); (d) variation with concentration of approximate molecular weight of each isomer of **II**; (e) concentration dependent diffusion coefficient of **III** (UPy ⋅ DAN *Z*, dark green and UPy ⋅ DAN *E*, dark blue; and, (f) variation with concentration of approximate molecular weight of each isomer of **III**. (dotted lines indicates the critical concentration).

Next, DOSY spectra were acquired at a range of concentrations of each supramolecular synthon (4–56 mM). As concentration increased, there was a decrease in D for all building blocks (Figure 3a) indicating an increase in size of the species. For **I**, at low concentrations (<24 mM), D decreased slightly for the *Z* isomer, whilst for the *E* isomer a similar trend was observed; D values were consistently slightly smaller for the *E* isomer than those for the *Z* isomer. Whilst these data imply limited chain elongation at lower concentrations this minor difference might be associated with the proclivity for the *Z* isomer to favor cyclic oligomers and the *E* isomer to favor extended oligomers (see later). At higher concentrations of **I** (>24 mM) the behavior differed, with the size of both the *E* and *Z* isomers both increasing to a comparable degree. This indicated a switch in the ring‐chain equilibria from cyclic species to chain extended oligomers. From these data, it was possible to calculate an approximate molecular weight (M_w_) for each isomer at each concentration. Assuming only monomeric species were present at 4 mM (for the *Z* form), we could calculate the M_w_ using the diffusion coefficients according to the following formula: M_w_=(D_monomer_/D)^3^.^[60]^ Conversion to molecular weight and plotting against concentration (Figure 2b), provides evidence to indicate both (*E)−* **I** and (*Z)−* **I** undergo concentration dependent step‐growth oligomerization.^[80]^ We also constructed a DOSY calibration curve using 2,2’‐dipyridyl and PMMA standards and determined M_w_ values from D values based on the calibration (see SI); this approach gave much lower M_w_ values which we consider to be less realistic given the dimerization affinities and concentrations in these systems.^[43]^ Overall, the data suggest that **I** forms short oligomers rather than supramolecular polymers which likely derives from the moderate strength for the triply hydrogen‐bonded heterodimerization.^[44]^

For **II**, there were more pronounced differences between the D values for the *E* and *Z* photostationary states at all concentrations (Figure 3c) when compared to **I**. Blue irradiated **II** was found to have lower D value than green light irradiated (*Z)−* **II** (which exhibited D values in a similar range to **I** in the low concentration regime). This implies a more significant difference in the assembly state for the *E* and *Z* states of **II**. For low concentration samples of **II** (<24 mM) D was approximately constant for the *Z* isomer. The D values for the *E* isomer when converted to M_w_ imply a species double the size of the *Z* isomer and exhibited a small decrease as the concentration increased, suggesting only moderate chain extension at these concentrations. Taken together, these data suggest the assembly state of **II** at low concentration differs between the *E* and *Z* states, which we attribute to the formation of a folded macrocycle for the *Z* isomer and cyclic hydrogen‐bonded oligomers (likely dimers) for the *E* isomer. Molecular modelling suggests that intramolecular hydrogen bonding for the *E* isomer is sterically suppressed, providing a rationale for the cyclization to give dimers in the low concentration regime (see SI). At higher concentrations of **II** (>24 mM) the D values for *Z* isomer solutions decreased moderately indicating a switch in the ring‐chain equilibria from cyclic species to oligomers. For the *E* isomer, the D values decreased more rapidly than those for the *Z* isomer indicating a switch from cyclic oligomers to supramolecular polymers. When converted to molecular weight and plotted against concentration (Figure 3d), it is evident that the species formed by (*E)−* **II** are significantly larger than any observed for I or (*Z)−* **II** samples consistent with step‐growth polymerization for (*E)−* **II** but oligomerization for (*Z)−* **II** at higher concentrations. Prior studies by K.‐D. Zhang and co‐workers on a shorter ditopic synthon bearing UPy units linked *via* a tetrafluoroazobenzene photoswitch reported DOSY data only at one concentration but could be used to ascertain the *E* isomer preferred a polydisperse assembly and the *Z* isomer former shorter oligomers with the smallest oligomer a cyclic dimer (see also discussion of viscosity data for **II** below).^[61]^

For **III**, more complex behavior was observed (Figure 3e). At 4 mM the difference in D value between blue irradiated (*E*)‐ **III** and green light irradiated (*Z)−* **III** differed as was the case for **II**. When converted to M_w_, the D values suggest the *E* isomer is larger than the Z isomer indicating the latter is monomeric whilst the former tends towards dimeric at low concentration. As concentration increases, the D value for the *Z* isomer undergoes small changes until ~24 mM and this then decreases at first dramatically and then from ~30 mM with a shallower gradient. For the *E* isomer D decreases significantly but in an uneven manner up to ~24 mM and then at a shallower gradient. Thus, the DOSY data imply three phases in the concentration dependent behavior of **III**. These phases are not as pronounced in the M_w_ versus concentration plots (Figure 3f) derived from the DOSY data and we interpret them to arise from the concentration dependent variation in the UPy ⋅ DAN and UPy ⋅ UPy speciation and associated chain stoppering effect.^[67]^ Taken together, the data suggest the assembly state of **III** at low concentration differs between the *E* (dimer) and *Z* (monomer) states, then switches to supramolecular polymers>24 mM. As for **II**, (*E)−* **III** and (*Z)−* **III** assemble to different extents or states, but both appear to form larger assemblies which we attribute to the higher UPy ⋅ DAN affinity.

### Viscosity measurements

**Figure 4:**
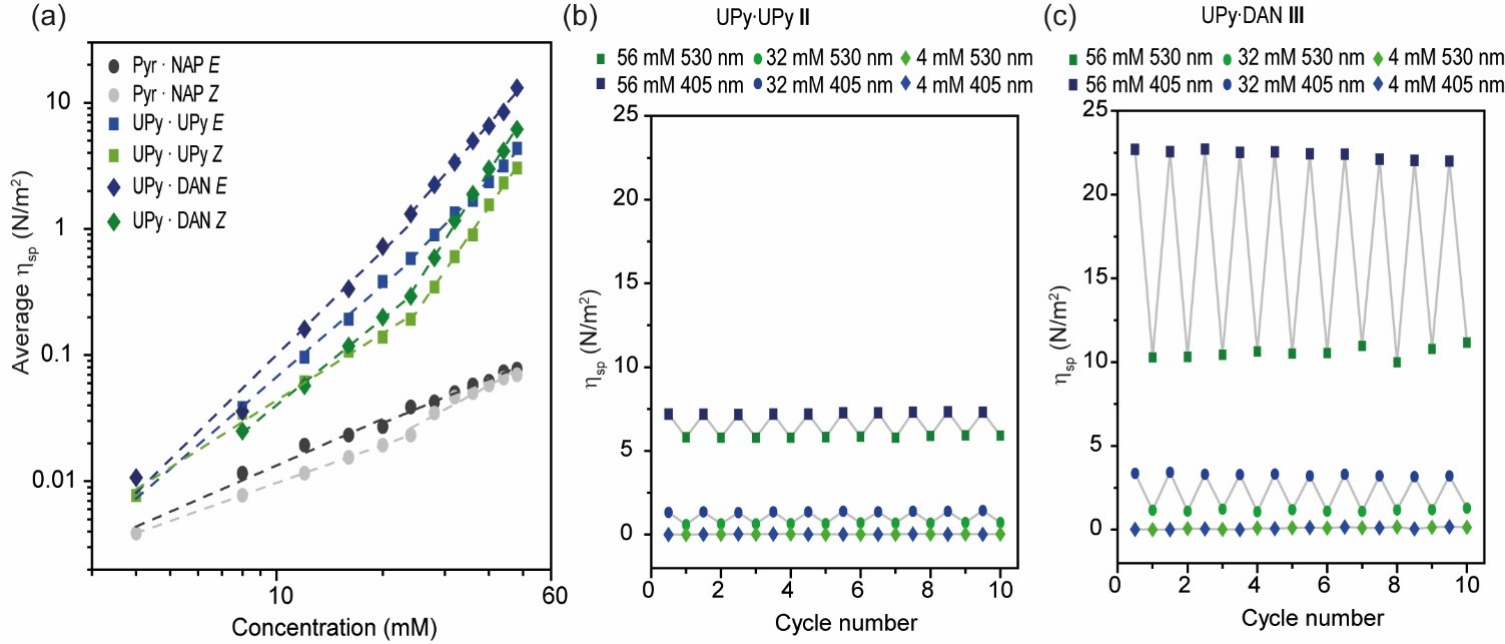
(a) Average specific viscosity of **I** (Pyr ⋅ NAP *E*, dark grey and Pyr ⋅ NAP *Z*, light grey), **II** (UPy ⋅ UPy *Z*, light green and UPy ⋅ UPy *E*, blue) **III** (UPy ⋅ DAN *Z*, dark green and UPy ⋅ DAN *E*, dark blue) at concentrations 4 mM‐ 56 mM. Solutions prepared in chloroform; (b) specific viscosity of **II** in response to ten successive cycles of light irradiation at 56, 32 and 4 mM concentrations (UPy ⋅ UPy *Z*, light green and UPy ⋅ UPy *E*, blue); and, (c) specific viscosity of **III** in response to ten successive cycles of light irradiation at 56, 32 and 4 mM concentrations (UPy ⋅ DAN *Z*, dark green and UPy ⋅ DAN *E*, dark blue).

To further assess the physical properties of **I–III**, viscosity measurements were performed on chloroform solutions of **I–III** using a micro‐Ostwald viscometer. A double‐logarithmic plot of the specific viscosity versus concentration was obtained (Figure 4a). (*Z)−* **I** exhibited only minor changes in viscosity between concentrations 4 mM–24 mM with a slope of ~1.0, suggesting small cyclic species were present. Above 24 mM the slope increased to ~1.5, indicating an increase in molecular weight.^[81]^ The viscosities of (*E)−* **I** increased steadily with concentration with a slope of 1.2, indicative of concentration dependent oligomerization – an inflection in the double‐logarithmic plot denoting a switch in ring‐chain bias is more difficult to perceive. Nonetheless, these findings were generally in agreement with the DOSY NMR results.

Below 24 mM a constant viscosity with a slope of 1.7 was observed for (*Z)−* **II**. Above 24 mM, the slope increased to 4.0, indicating oligomerization. In comparison to DOSY NMR data, which indicated a small increase in size, the viscosities measured for **II** suggest a more significant increase in molecular weight in both concentration regimes. A possible explanation for this is due to the folded shape of (*Z)−* **II**. Viscosity, depends also on solvation^[82]^ and shape^[83]^ for instance single stranded DNA has lower intrinsic viscosity than double stranded DNA, however at low ionic strength this trend can be reversed. We thus interpret the data to indicate (*Z)−* **II** forms a self‐assembled foldamer with shorter more compact and rigid structure. Given diffusion coefficient is correlated with the hydrodynamic radius of a spherical molecule, differences in shape between oligomers of (*Z*)‐ and (*E)−* **II** are unlikely to be accounted for. This relationship, defined by the Stokes‐Einstein equation, may offer a reasonable explanation for the discrepancy between the DOSY and viscosity data.^[84]^ The viscosities for (*E)−* **II** increased more steadily with concentration with a slope of 2.6, indicative of concentration dependent polymerization across the concentration gradient; an inflection point for the critical concentration (~24 mM) denoting transition from the cyclic to oligomeric regime in the double logarithmic plot can be perceived (Figure 4a). In the prior report by K.‐D. Zhang and co‐workers,^[61]^ a shorter ditopic synthon bearing UPy units linked *via* a tetrafluoroazobenzene photoswitch was reported to form cyclic dimers in the *Z* form which oligomerize with a critical concentration of ~45 mM whereas the *E* form assembled oligomers according to an isodesmic relationship. In contrast, richer behavior is observed here; taken together, the DOSY and viscosity data indicate (*E)−* **II** appears to prefer self‐assembled dimers in the low concentration regime and oligomerizes at higher concentration with a critical concentration of ~24 mM, whilst (*Z)−* **II** adopts an intramolecular hydrogen‐bonded monomeric configuration at low concentration and oligomerizes at higher concentration with a critical concentration of ~24 mM to produces a different self‐assembled structure to (*E)−* **II**.

Below 24 mM a constant viscosity with a slope of 2.3 was observed for (*Z)−* **III**. Above the critical concentration the slope changes to 4.4. The viscosities for (*E)−* **III** increased steadily with concentration with a slope of 2.7. An inflection point for the critical concentration denoting transition from the cyclic to oligomeric regime in the double logarithmic plot was observed ~24 mM at which point the slope increased to 3.2 (Figure 4a). Overall, the viscosity data for **III** indicate similar ring‐chain behaviour to that observed for **II** with the *Z* isomer exhibiting a greater tendency than the *E* isomer towards ring formation below the critical concentration (24 mM) and then above the critical concentration, both isomers oligomerizing with the *E* isomer forming more viscous assemblies. In each case the *E* and *Z* isomers of **III** are more viscous than the corresponding *E* and *Z* isomers of **II** in the higher concentration regime, which is consistent with the DOSY results which indicated **III** forms longer polymers than **II**.

To demonstrate reversible property switching, multiple cycles of irradiation were carried out at three concentrations (4 mM, 32 mM and 56 mM) for **II** (Figure 4b) and **III** (Figure 4c). In both cases the variation in viscosity was negligible at 4 mM, more significant at 32 mM and most significant at 56 mM). It was possible to switch the viscosities of **II** from approximately 7.25 N/m^2^ to approximately 5.75 N/m^2^ and for **III** from approximately 10.60 N/m^2^ to approximately 22.40 N/m^2^ through 10 cycles of irradiation under blue and green light (Figure 4b) demonstrating the robust nature of the physical property photoswitching. The greater variation in viscosity for **III** is consistent with our interpretation that **III** can form longer supramolecular foldamers than **II** driven by the higher affinity of the UPy ⋅ DAN interaction in comparison to the UPy ⋅ UPy interaction.

## Conclusions

**Figure 5:**
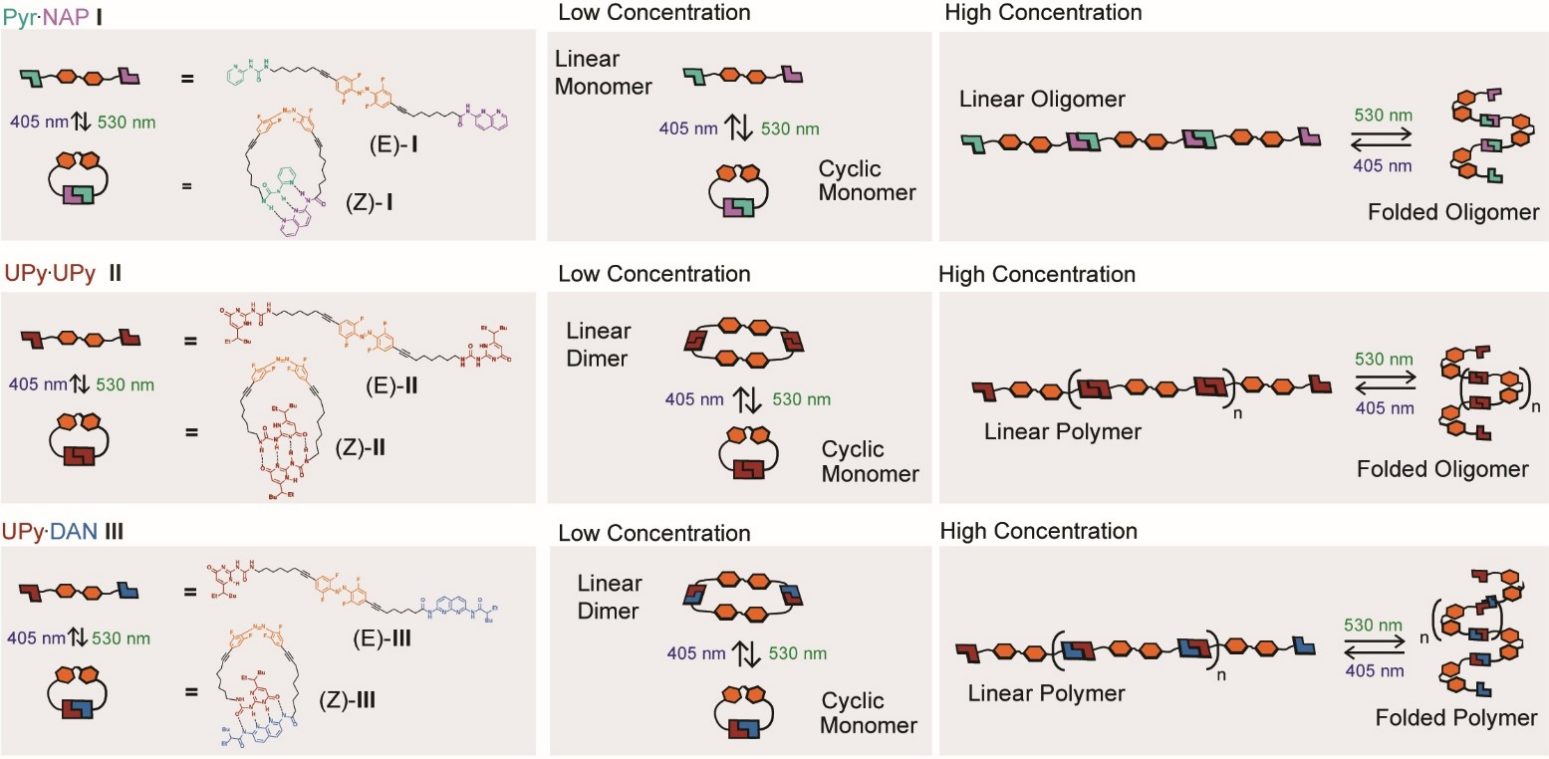
Schematic representing the architectures formed by the *E* and *Z* isomers of **I–III** at high and low concentrations: Pyr.NAP **I** at low concentrations switches between monomeric cyclic and linear structures, and at higher concentions switches between linear and folded oligomers. UPy.UPy **II**, at low concentrations switches between monomeric and dimeric cyclic structures and and at higher concentrations switches between linear polymers and folded oligomers in response to green/blue light. UPy.DAN **III**, at low concentrations switches between monomeric and dimeric cyclic structures and and at higher concentrations switches between linear polymers and folded polymers in response to green/blue light.

The photoresponsive oligomerisation and polymerization behaviours of the *E* and *Z* isomers of **I–III** have been characterized by DOSY NMR and viscosity studies. This allowed assessment on the effects of geometric orientation and strength of hydrogen‐bonding sites on ring‐chain equilibria in the context of a photoswitchable scaffold (Figure 5). At concentrations below 24 mM, a cyclic hydrogen‐bonded monomer dominates for (*Z)−* **I** whereas the linear non‐hydrogen‐bonded form is preferred for (*E)−* **I**. At concentrations above 24 mM both (*Z)−* **I** and (*E)−* **I** undergoes a shift in the ring‐chain equilibria toward oligomers, but hydrogen‐bonding is too weak to form polymers. **II** is able to form significantly larger molecules than **I** due to the higher association constant between the HBMs (*K*_a_ = 30 M^‐1^ v *K*_dim_ = 6 x 10^7^ M^‐1^ in chloroform). From viscosity and DOSY studies, it appears that below 24 mM **II** is able to switch in preference between cyclic monomers {(*Z*)−**II**} and dimers {(*E*)−**II**} as the dominant species. Above 24 mM larger assemblies form: it was possible to switch between a more compact/shorter assembly for (*Z)−* **II** and larger/longer assembly with higher viscosity for (*E)−* **II** in response to visible light, highlighting the important role of conformation in the assembly of these supramolecular foldamers. We note that a distribution of *E* and *Z* isomers is present at the photostationary state, however this points to four dominant states: cyclic monomer or dimer at low concentration and compact or extended supramolecular foldamer at high concentration. **III**, incorporating a quadruply hydrogen‐bonded heterodimer represents the first photoswitchable *AB* type hydrogen‐bonded supramolecular polymer. Whilst the behavior for **III** is further complicated by competition between DAN ⋅ UPy heterodimerization and UPy ⋅ Upy homodimerization, four dominant states are observed as is the case for **II**, however longer supramolecular foldamers form with higher viscosity pointing to the importance of hydrogen‐bond strength in regulating assembly. Whilst we cannot unambiguously establish that the change in properties on photoswitching arises from oligomers with different folded conformations, this seems likely; steric effects could result in a differing degrees of polymerization for *Z* (**II** or **III**) and *E* (**II** or **III**), however our ^1^H NMR data indicate that both the UPy ⋅ UPy (II) and UPy ⋅ Dan (**III**) assemblies remain hydrogen‐bonded over the entire concentration range studied (see ^1^H projections in the DOSY spectra in SI), disfavoring depolymerization as the origin of the observed changes in viscosity. Taken together the observed visible light responsive concentration and photoswitching (Figure 5) offers opportunities for regulation of more complex functions (noting the sol‐gel transition observed for a ditopic synthon bearing UPy units observed at higher concentration (~500 mM) on photoswitching in the previous report by K.‐D. Zhang and co‐workers^[61]^); we will pursue such objectives in future research.

## Supporting Information

Synthetic methods, characterization, additional DOSY data, viscometry measurements, molecular modelling and cahracterization data. The authors have cited additional references within the Supporting Information[^53^, ^68^, ^85^, ^86^, ^87^, ^88^, ^89^, ^90^, ^91^, ^92^]

## Author Contributions

NJW and AJW conceived the studies; EH performed syntheses and characterization, photoswitching, DOSY and viscometry analyses. MJ performed additional synthesis, molecular modelling and molecular dynamics simulations. ADB performed molecular modelling and molecular dynamics simulations. EH and AJW wrote the manuscript, which was edited into its final form by AJW with contributions from all authors

## Conflict of interests

There are no conflicts to declare.

1

## Supporting information

As a service to our authors and readers, this journal provides supporting information supplied by the authors. Such materials are peer reviewed and may be re‐organized for online delivery, but are not copy‐edited or typeset. Technical support issues arising from supporting information (other than missing files) should be addressed to the authors.

Supporting Information

## Data Availability

The data that support the findings of this study are available in the supplementary material of this article.
