# Supplementary material for: Visible‐Light Driven Control Over Triply and Quadruply Hydrogen‐Bonded Supramolecular Assemblies
Source: Chemistry. 2024 Feb 19;30(20):e202304033. doi: 10.1002/chem.202304033 (PMC11497329; doi:10.1002/chem.202304033)
Supplement: Supplementary file 1 — Supporting Information [file CHEM-30-e202304033-s001.pdf]

Chemistry–A European Journal

Supporting Information

Visible-Light Driven Control Over Triply and Quadruply Hydrogen-Bonded Supramolecular Assemblies

Eleanor M. Hilton, Michael A. Jinks, Andrew D. Burnett, Nicholas J. Warren,* and Andrew J. Wilson*

Supporting Information

Visible-Light Driven Control Over Triply and Quadruply Hydrogen-Bonded Supramolecular Assemblies

Eleanor M. Hilton,[a,b] Michael A. Jinks,[a] Andrew D. Burnett,[a] Nicholas J. Warren*[b] and Andrew J. Wilson*[a,c,d]

a. School of Chemistry, University of Leeds, Woodhouse Lane, Leeds, LS2 9JT, UK. E-mail: a.j.wilson@leeds.ac.uk

b. School of Chemical and Process Engineering, University of Leeds, Woodhouse Lane, Leeds LS2 9JT, UK E-mail: N.Warren@leeds.ac.uk

c. Astbury Centre for Structural Molecular Biology, University of Leeds, Woodhouse Lane, Leeds, LS2 9JT, UK

d. School of Chemistry, University of Birmingham, Edgbaston, Birmingham B15 2TT, UK

Contents

1. Notes and Schemes for Foldamer Syntheses 1

2. General experimental considerations 4

2.2 Characterisation 4

3. Compound Synthesis 5

4. Photoisomerization studies 14

5. DOSY NMR 15

5.1 DOSY Spectra 15

5.2 DOSY Data 29

5.2.1 Pyr.NAP Foldamer I 29

5.2.2 UPy.UPy Foldamer II 30

5.2.3 UPy.DAN Foldamer III 31

6. Viscometry 33

6.1 Pyr.NAP Foldamer I 33

6.2 UPy.UPy Foldamer II 34

6.3 UPy.DAN Foldamer III 37

7. Molecular Modelling 39

8. NMR Spectra 42

8.1 ^{1}H NMR Spectra 42

8.2 ^{13}C NMR Spectra 49

8.3 ^{19}F NMR Spectra 55

9.0 References 57

1. Notes and Schemes for Foldamer Syntheses

The convergent synthesis of foldamer **I** (scheme 1) used literature methods[1-2] and was adapted from a pre-established synthetic procedure described by Opie and co-workers.[3]

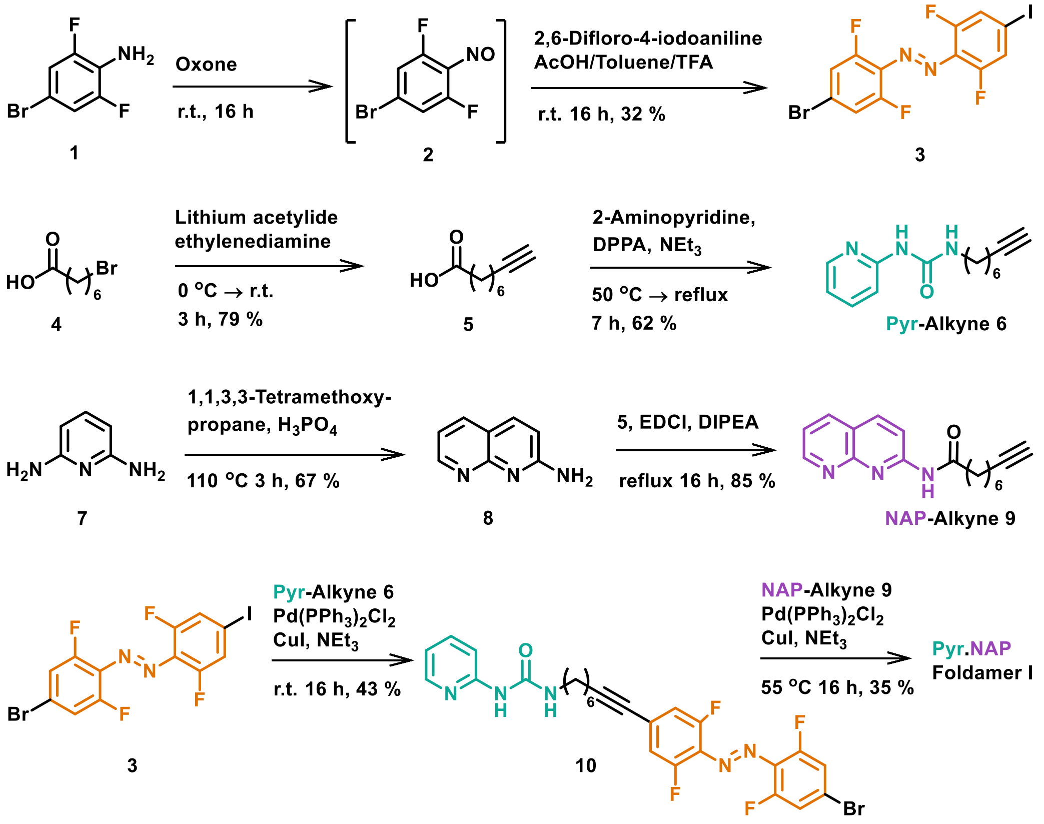

Scheme 1. *Synthesis of foldamer **I**, conitaing pyridylurea (Pyr) and amidonaphthyridine (NAP) triple hydrogen bonding motifs.*

Despite being symmetrical, foldamer **II** was synthetically more challenging. It was possible to synthesise the UPy-functionalised alkyne **12** *via* a similar route to that followed for pyridylurea urea linker **6** (Scheme 2). In an attempt to improve yields and reduce the number of synthetic steps, diiodo-o-tetrafluorinated azobenzene **14** was synthesised from aniline **13**. However, upon attempting a double Sonogashira coupling foldamer **II** was produced only in low yield and could not practicably be isolated.

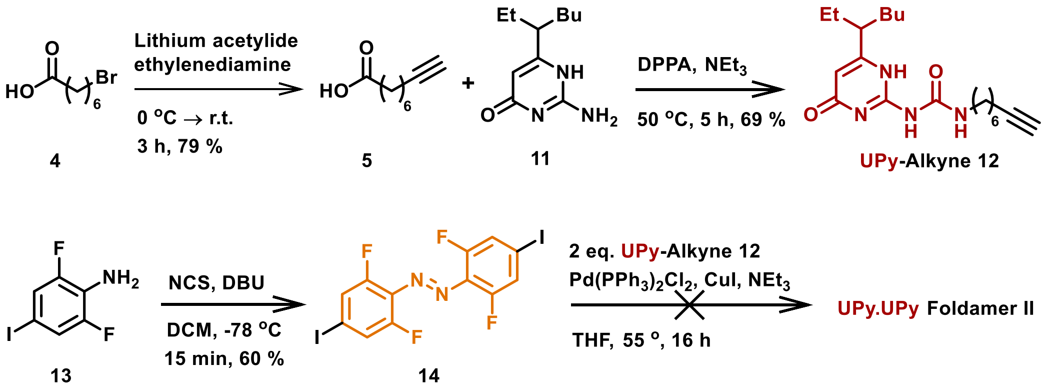

***Scheme 2**. Attempted synthesis of UPy·UPy foldamer **II** using diiodo-azobenzene **14**.*

To explore if the reactivity of the diiodo-*o*-fluorinatedazobenzene **14** was associated with low yield of foldamer **II**, the synthesis was carried out using the *o*-tetrafluorinated azobenzene **3** in multiple steps. The first coupling was successful, and yielded product **15** in 60% yield. The second Sonogashira reaction was, however, much less successful with very little conversion to product observed (~1 %, determined by ^{1}H NMR). Variation of catalyst and ligand had minimal effect upon the yield. We therefore hypothesized that the low conversion to product was associated with homo and heteroassociation between **12** and **15** and that such interactions might sterically impede appropriate orientations for coupling to occur. The more facile synthesis of foldamer **I** is congruent with this theory and the much weaker association of the pyridylurea·amidonaphthyridine interaction than the ureidopyrimidinone dimerization. The synthesis of foldamer **II** was therefore attempted using polar solvents to prevent hydrogen bonding interactions between the UPy functionalised molecules **12** and **15**. Reactions carried out in competitive solvents (dimethylformamide, triethylamine and diisopropylethylamine) yielded product, whereas those performed in tetrahydrofuran did not. Despite better conversion in competitive solvents, association of the unreacted **12** and **15** with product likely contribute to subsequent difficult purification of foldamer **II**; although the yield of 13 % was relatively low, it was sufficient for subsequent characterization.

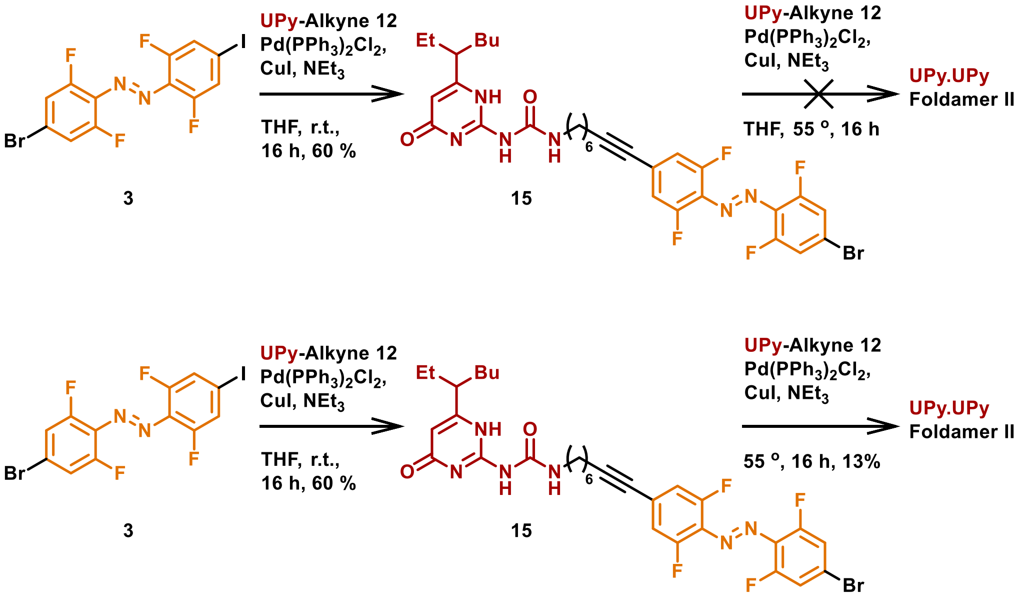

Scheme 3. *Syntheses of UPy·UPy foldamer **II** using iodobromo-azobenzene **3**.*

Synthesis of foldamer **III** was achieved through preparation of DAN appended alkyne **22** following established routes for preparation of unsymmetrical DAN motifs.[4-5] Starting with 2,6-diaminopyridine **6**, heterocycle formation to give **18** followed by acylation to give **19**, then chlorination to give **20** and amination to give **21**, amide bond formation with 8-noynoic acid **5** yielded the alkyne which underwent Sonogashira coupling with intermediate **15** to give the target foldamer III (scheme 4).

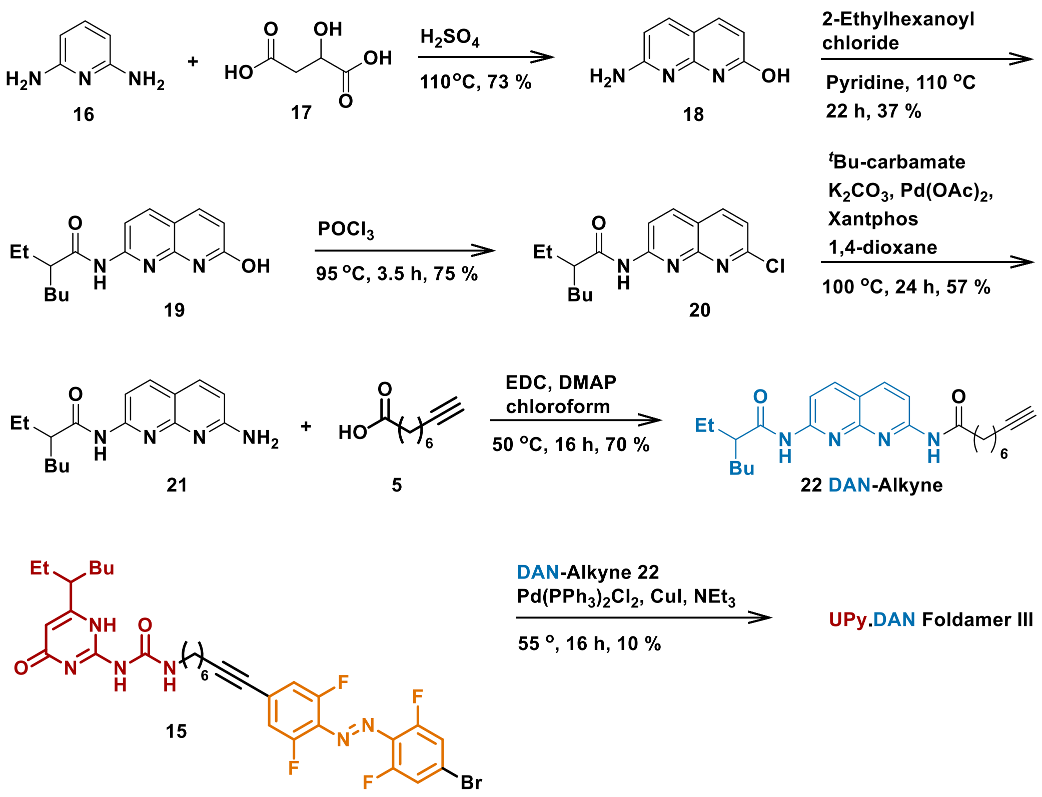

Scheme 4. *Syntheses of UPy·DAN foldamer **III** using iodobromo-azobenzene **15** and DAN alkyne **22**.*

2. General experimental considerations

2.1 Synthesis

Solvents and reagents were purchased from Sigma Aldrich, Fluorochem or Fisher Scientific and used without further purification unless otherwise stated. Where anhydrous solvents or reagents were required, tetrahydrofuran was obtained from the in-house solvent purification system Innovative Inc. PureSolv®, anhydrous triethylamine was purchased from Fluorochem and anhydrous dimethylsulfoxide from Sigma Aldrich. All non-aqueous reactions were carried out in oven-dried glassware under a nitrogen atmosphere with magnetic stirrer bars, unless otherwise stated. All work-up and purification procedures were carried out using reagent-grade solvents under ambient atmosphere. Analytical thin layer chromatography was performed on Merck Kieselgel 60 F_{254} 0.25 mm pre-coated aluminium plates and visualised by UV quenching (λ_{max}=254 nm). Flash chromatography was carried out using Merck Kieselgel 60 silica gel. HPLC was performed with an Agilent Technologies 1290 Infinity analytical preparative system equipped with a Kinetex EVO C18 reverse-phase column (ϕ 21.2 x 250 mm).

2.2 Characterisation

For NMR experiments anhydrous chloroform-*d* was purchased from Sigma Aldrich. All ^{1}H NMR spectra were acquired on Bruker AVANCE spectrometers, operating at 400 MHz or 500 MHz for ^{1}H, 100 MHz or 125 MHz for ^{13}C and 375 MHz for ^{19}F. Nuclear magnetic resonance spectra were obtained at 298 K and referenced using residual solvent signals as internal standards unless stated otherwise. The spectrometers used for 1D experiments were either a two-channel Bruker AV3HD NMR spectrometer operating at 9.4 T (400 MHz ^{1}H) equipped with a 5 mm BBO probe or a two-channel Bruker AV-NEO NMR spectrometer operating at 11.7 T (500 MHz ^{1}H) equipped with a 5 mm DCH cryoprobe. DOSY spectra were obtained using a four-channel Bruker AV-NEO NMR spectrometer operating at 11.7 T (500 MHz ^{1}H) and equipped with 5mm TXI probe (δ = 0.002 s, Δ = 0.0999). Chemical shifts are expressed in parts per million (ppm) and the following abbreviations are used: s (singlet), d (doublet), t (triplet), q (quartet), m (multiplet), br (broad), and app (apparent). Infra-red spectra were obtained using a PerkinElmer FTIR spectrometer in which absorption maxima (ν_{max}) are expressed in wavenumbers (cm^{-1}). High-resolution mass spectrometry (HRMS) was performed using a Bruker maXis Impact QTOF mass spectrometer, with an electrospray ionisation (ESI) source. UV-Vis absorption spectra were recorded on an Agilent Technologies Cary Series UV-Vis spectrophotometer.

3. Compound Synthesis

1-(4-Bromo-2,6-difluorophenyl)-2-(2,6-difluoro-4-iodophenyl)diazene 3

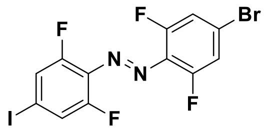

Procedure adapted from literature.[6] 2,6-Difluoro-4-bromoaniline **1** (0.85 g, 4.06 mmol, 1.0 eq) was dissolved in chloroform (10 mL) and stirred. Oxone (5.00 g, 8.13 mmol, 2.0 eq) was dissolved in water (40 mL) and added to the chloroform solution dropwise. The biphasic mixture was stirred for 16 h and the product extracted into chloroform (3 x 20 mL). The combined organic extracts were washed with 1 M hydrochloric acid solution (2 x 20 mL), saturated sodium bicarbonate solution (2 x 20 mL), water (2 x 20 mL) and brine (2 x 20 mL), then dried with magnesium sulfate, filtered and concentrated to give a brown solid. Without further purification the crude product was suspended in a mixture of toluene, acetic acid and trifluoroacetic acid (1:1:0.17), then 2,6-difluoro-4-iodo-analine (0.88 g, 3.45 mmol, 0.85 eq) was added. The reaction was stirred for 16 h then concentrated to give a brown solid. The product was isolated via column chromatography (SiO_2, 6:4 hexane: dichloromethane), then crystallised from ethanol, to give the title compound **3** as red needles (0.51 g, 1.10 mmol, 38 %). ^{1}H NMR (500 MHz, chloroform-*d*) δ 7.53 – 7.46 (m, 2H, 2 x ArI-*H*), 7.30 (dd, 2H, J 6.9, 4.9 Hz, 2 x ArBr-*H*); ^{13}C NMR (100 MHz, chloroform-*d*) δ 156.6 (d), 153.9 (d), 134.1 (t), 130.8 (t), 122.5 (dd), 116.8 (dd), 94.7 (t); ^{19}F NMR (375 MHz, chloroform-*d*) δ -118.6 (d), -119.5 (d); Rf 0.57 (60:40 hexane: dichloromethane); IR ν_{max} (solid state) = 3476.9, 3366.7, 2345.9, 1595.2 cm^{-1}; ESI-HRMS *m/z* found 458.8613 $[M + H]^+$ $C_{12}H_5BrF_4IN_2$ requires 458.8611.

8-Nonynoic acid 5

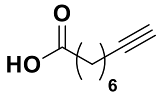

Procedure adapted from literature.[6] 7-Bromoheptanoic acid **4** (1.9 g, 9.1 mmol, 1 eq) in anhydrous dimethyl sulfoxide (3 mL) was added to suspension of lithium acetylide ethylenediamine complex (3.3 g, 36.4 mmol, 4 eq) in anhydrous dimethyl sulfoxide (30 mL) dropwise at 0 °C over 30 min. After stirring at 0 °C for 1 h, the solution was warmed to room temperature, and stirred for a further 2 h. The reaction mixture was quenched by pouring onto 10% sulfuric acid solution (225 mL) at 0 °C. The aqueous phase was extracted with hexane (3 x 100 mL) and the combined organic fractions were dried over sodium sulfate, filtered and concentrated to give the title compound as a light pink liquid (1.09 g, 7.1 mmol, 78%). ^{1}H NMR (400 MHz, chloroform-*d*) δ 11.45 (s, 1H, COO*H*), 2.31 (t, *J* 7.5 Hz, 2H, C*H*$_2$COOH), 2.14 (td, *J* 7.0, 2.6 Hz, 2H, C*H*$_2$CCH), 1.91 (t, *J* 2.6 Hz, 1H, CH$_2$CC*H*), 1.60 (p, *J* 7.5 Hz, 2H, C*H*$_2$), 1.53 – 1.21 (m, 6H C*H*$_2$C*H*$_2$C*H*$_2$); ^{13}C NMR (100 MHz, chloroform-*d*) δ 180.3, 84.3, 68.2, 33.9, 28.3, 28.1, 28.0, 24.3, 18.1; Rf 0.74 (1:1 hexane: ethyl acetate); IR ν_{max} (solid state) = 3300.7, 2935.7, 2116.9, 1703.6 cm^{-1};ESI-HRMS *m/z* found 153.0913 $[M - H]^-$ $C_9H_{13}O_2$ requires 153.0921.

1-(Oct-7-yn-1-yl)-3-(pyridin-2-yl)urea 6

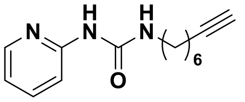

Procedure adapted from literature.[6] Triethylamine (0.18 mL, 1.31 mmol, 1 eq) and diphenylphosphoryl azide (0.28 mL, 1.31 mmol, 1 eq) were added to a solution of 8-nonynoic acid **5** (0.20 g. 1.31 mmol, 1 eq) in acetonitrile (5 mL). After stirring for 2 h at 50 °C, 2-aminopyridine (0.12 g, 1.31 mmol, 1 eq) was added, and the solution was heated to reflux for 5 h. The solution was concentrated, and the residue purified by column chromatography (SiO_2, 1:1 hexane: ethyl acetate) to give the title compound **6** as a white solid (0.20 g, 0.82 mmol, 62 %). ^{1}H NMR (500 MHz, chloroform-*d*) δ 9.36 (s, 1H, N*H*), 8.67 (s, 1H, N*H*), 8.18 (ddd, 1H, *J* 5.2, 1.9, 0.9 Hz, Pry-*H*), 7.76 – 7.48 (m, 1H, Pry-*H*), 6.97 – 6.76 (m, 2H, 2 x Pry-*H*), 3.41 (td, 2H, *J* 7.1, 5.7 Hz, NHCONH-C*H₂*), 2.22 (tdd, 2H, *J* 7.1, 2.7, 0.9 Hz, HCC-CH_2), 1.96 (td, 1H, *J* 2.7, 0.9Hz, CC*H*), 1.70 – 1.61 (m, 2H, CH_2), 1.61 – 1.54 (m, 2H, CH_2), 1.53 – 1.39 (m, 4H, 2 x CH_2); ^{13}C NMR (125 MHz, chloroform-*d*) δ 156.2, 153.5, 145.7, 138.4, 112.0, 84.6, 68.2, 39.8, 29.9, 28.4, 28.4, 26.5, 18.4; Rf 0.49 (1:1 hexane: ethyl acetate); IR ν_{max} (solid state) = 3244.4, 2919.0, 1678.9 cm^{-1}; ESI-HRMS *m/z* found 246.1610 $[M + H]^+$ $C_{14}H_{20}N_3O$ requires 246.1601.

1,8-Naphthyridin-2-amine 8

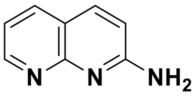

Procedure adapted from literature.[6] 2,6-Diaminopyridine **7** (5.0 g, 46.0 mmol, 1 eq) was suspended in polyphosphoric acid (30 g). After stirring at 90 °C for 1 h, 1,1,3,3-tetramethoxypropane (7.9 mL, 48.0, 1.05 eq) was added dropwise over 15 min. The solution was heated to 110 °C and stirred for 4 h. After cooling to room temperature, chloroform (180 mL) and water (90 mL) were added. A solution of 10 N sodium hydroxide (180 mL) was used to quench the reaction at 0 °C, the resulting brown precipitate was removed via filtration and washed with chloroform. The organic extracts were combined, dried with sodium sulfate, filtered, and concentrated. The residue was purified by column chromatography (SiO_2, 9:1 dichloromethane: methanol) to give the title compound **8** as a yellow powder (2.67 g, 18.4 mmol, 40 %). ^{1}H NMR (400 MHz, chloroform-*d*) δ 8.88 (dd, 1H, *J* 4.4, 2.0 Hz, Ar*H*), 7.97 (dd, 1H, *J* 7.9, 2.0 Hz, Ar*H*), 7.89 (d, 1H, *J* 8.7 Hz, Ar*H*), 7.22 (dd, 1H, *J* 7.9, 4.4 Hz, Ar*H*), 6.82 (d, 1H, *J* 8.7 Hz, NH_2-Ar*H*), 5.27 (br s, 2H, NH_2); ^{13}C NMR (125 MHz, chloroform-*d*) δ 159.4, 156.6, 152.9, 138.4, 136.3, 118.4, 117.6, 112.6; Rf 0.38 (9:1 dichloromethane: methanol); IR ν_{max} (solid state) = 3314.5, 3161.5, 1617.5 cm^{-1}; ESI-HRMS *m/z* found 146.0711 $[M + H]^+$ $C_8H_8N_3$ requires 146.0713.

N-(1,8-Naphthyridin-2-yl)non-8-ynamide 9

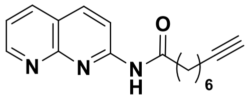

Procedure adapted from literature.[6] EDC (1.29 g. 8.3 mmol, 1.2 eq) was added to a solution of 8-nonynoic acid **5** (1.28 g, 8.3 mmol, 1.2 eq) and 4-dimethylaminopyridine (1.26 g, 10.3 mmol, 1.5 eq) in chloroform (50 mL). After stirring for 30 min, 1,8-naphthyridin-2-amine **8** (1.00 g, 6.9 mmol, 1 eq) was added, and the solution was stirred at 50 °C for 16 h. Chloroform was then removed by rotary evaporation, and the crude material redissolved in ethyl acetate (50 mL), and washed with 1M hydrochloric acid (2 x 20 mL), saturated sodium bicarbonate solution (2 x 20 mL) and brine (20 mL). After drying over sodium sulfate and concentration in vacuo, title compound **9** was afforded as a white solid (2.34 g, 7.9 mmol, 85 %). ^{1}H NMR (500 MHz, chloroform-d) δ 9.62 (s, 1H, N*H*), 9.07 (dd, 1H, J 4.3, 1.8 Hz, Ar*H*), 8.63 (d, 1H, J 8.9 Hz, Ar*H*), 8.30 (d, 1H, J 8.9 Hz, Ar*H*), 8.23 (d, 1H, J 8.0 Hz, Ar*H*), 7.51 (dt, 1H, J 11.2, 5.6 Hz, Ar*H*), 2.60 (t, 2H, J 7.4 Hz, HNCOC*H₂*), 2.22 (td, J 6.9, 2.6 Hz, 2H, HCC-C*H₂*), 1.97 (t, 1H, J 2.6 Hz, CC*H*), 1.82 (p, 2H, J 7.5 Hz, C*H₂*), 1.63 – 1.54 (m, 2H, C*H₂*), 1.49 (ddt, 4H, J 11.0, 8.5, 5.5 Hz, 2 x C*H₂*);^{13}C NMR (125 MHz, chloroform-*d*) δ 172.8, 154.0, 153.8, 153.6, 139.9, 136.9, 121.1, 120.6, 115.4, 84.5, 68.3, 37.9, 28.6, 28.4, 28.2, 25.0, 18.3; Rf 0.30 (ethyl acetate); IR v_{max} (solid state) = 3291.3, 2932.3, 1695.6 cm^{-1}; ESI-HRMS m/z found 282.1608 [M + H]+ $C_{17}H_{20}N_3O$ requires 282.1601.

1-(8-(4-((4-Bromo-2,6-difluorophenyl)diazenyl)-3,5-difluorophenyl)oct7-yn-1-yl)-3-(pyridin-2-yl)urea 10

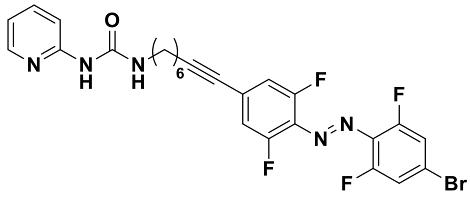

Procedure adapted from literature.[6] 1-(4-Bromo-2,6-difluorophenyl)-2-(2,6-difluoro-4-iodophenyl)diazene **3** (1.70, 3.71, 1.0 eq), copper iodide (5 mol%), bis(triphenylphosphine)palladium(II) dichloride (2 mol%), and 1-(oct-7-yn-1-yl)-3-(pyridin-2-yl)urea **6** (1.00 g, 4.08 mmol, 1.1 eq) were added to an oven dried flask. The flask was evacuated and back-filled with nitrogen three times, before anhydrous tetrahydrofuran (20 mL) was added. Anhydrous triethylamine (2.59 mL, 18.6 mmol, 5 eq) was prepared via three freeze, pump thaw cycles then added to the reaction mixture. The solution was stirred under nitrogen at room temperature for 16 h. The solvent was removed and the crude red solid purified via column chromatography (SiO_2, 1:1 hexane: ethyl acetate). The resulting red solid was crystallised from acetonitrile to give the title compound **10** as an orange powder (1.30 g, 2.26 mmol, 61 %). ^{1}H NMR (400 MHz, chloroform-*d*) δ 9.24 (s, 1H, N*H*), 8.17 (dd, 1H, *J* 5.4, 2.1 Hz, Pyr*H*), 7.74 – 7.57 (m, 1H, Pyr*H*), 7.32 – 7.27 (m, 2H, 2 x Br-AB-*H*), 7.13 – 7.02 (m, 2H, 2 x AB-*H*), 6.98 – 6.78 (m, 2H, 2 x Pyr*H*), 3.42 (td, 2H, *J* 7.0, 5.6 Hz, NHCONH-C*H₂*), 2.47 (t, 2H, *J* 7.0 Hz, CC-C*H₂*), 1.70 (dd, 4H, *J* 14.1, 7.1 Hz, 2 x C*H₂*), 1.59 – 1.41 (m, 4H, 2 x C*H₂*); ^{13}C NMR (125 MHz, chloroform-*d*) δ 156.5 (dd), 155.8, 154.4 (d), 153.1, 145.4, 138.8, 130.9 (q), 128.4 (t), 124.0 (t), 116.9 – 116.4 (m), 116.1 – 115.3 (m), 112.1, 96.2, 78.9 (t), 39.8, 29.8, 28.4 (d), 26.5, 19.5; ^{19}F NMR (375 MHz, chloroform-*d*) δ -118.9 (d), -120.6 (d);

Rf 0.29 (1:1 hexane: ethyl acetate); IR ν_{max} (solid state) = 3219.1, 3118.5, 2920.9, 2856.7, 2232.6, 1677.8 cm^{-1}; ESI-HRMS *m/z* found 576.1041 $[M + H]^+$ $C_{26}H_{23}BrF_4N_5O$ requires 576.1017.

9-(4-((2,6-Difluoro-4-(8-(3-(pyridin-2-yl)ureido)oct-1-yn-1-yl)phenyl)diazenyl)-3,5-difluorophenyl)-N-(1,8- naphthyridin-2-yl)non-8-ynamide Foldamer I

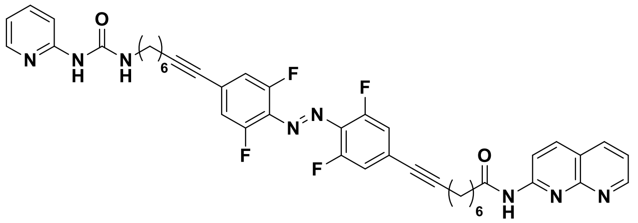

Procedure adapted from literature.[6] 1-(8-(4-((4-Bromo-2,6-difluorophenyl)diazenyl)-3,5-difluorophenyl)oct7-yn-1-yl)-3-(pyridin-2-yl)urea **10** (0.20 g, 0.35 mmol, 1.0 eq), bis(triphenylphosphine)palladium(II) dichloride (2 mol%), copper iodide (5 mol%) and n-(1,8-naphthyridin-2-yl)non-8-ynamide **9** (0.10 g 0.35 mmol, 1.0 eq) were added to an oven dried flask. The flask was evacuated and back-filled with nitrogen three times, before anhydrous tetrahydrofuran (100 mL) was added. Anhydrous triethylamine (0.24 mL, 1.75 mmol, 5.0 eq) was prepared via three freeze, pump thaw cycles then added to the reaction mixture. The solution was stirred under nitrogen at room temperature for 16 h. The solvent was removed and the crude red solid purified via column chromatography (SiO_2, ethyl acetate). The resulting red solid was further purified by reverse phase HPLC (50-60 % MeCN in H2O) the product was isolated by lyophilisation to afford foldamer **I** as an orange powder (0.10 g, 0.12 mmol, 35 %). *E* isomer - ^{1}H NMR (500 MHz, chloroform-*d*) δ 8.96 (s, 1H, N*H*), 8.83 (s, 1H, N*H*), 8.51 (d, 1H, *J* 8.9 Hz, Nap-*H*), 8.31 – 7.90 (m, 4H, 2 x Nap-*H,* 2 x Pyr-*H*), 7.82 – 7.70 (m, 1H, Nap-*H*), 7.47 – 7.23 (m, 2H, Nap-*H*, Pyr-*H*), 7.04 – 6.90 (m, 5H, 4 x AB-*H*, Pyr-*H*), 3.29 (q, 2H, *J* 6.4 Hz, NHCONH-C*H₂*), 2.47 (t, 2H, *J* 7.5 Hz, CONH-C*H₂*), 2.38 (td, 4H, *J* 7.0, 2.6 Hz, 2 x CC-C*H₂*), 1.75 (q, 2H, *J* 7.4 Hz, C*H₂*), 1.57 (q, 6H, *J* 7.2 Hz), 1.46 – 1.35 (m, 8H, 4 x C*H₂*); *Z* isomer - ^{1}H NMR (500 MHz, chloroform-*d*) δ 10.32 (s, 1H, N*H*), 9.10 (s, 1H, N*H*), 9.04 (s, 1H, N*H*), 8.61 (t, 1H, *J* 9.3 Hz, Nap-*H*), 8.33 – 8.08 (m,4H, 2 x Nap-*H,* 2 x Pyr-*H*), 7.76 – 7.64 (m, 1H, Nap-*H*), 7.46 (m, 2H, Nap-*H*, Pyr-*H*), 7.10 – 7.03 (m, 2H, 2 x AB-*H*), 6.95 (dt, 1H, *J* 13.6, 6.1 Hz, Pyr-*H*), 6.87 (dd, 2H, *J* 7.8, 2.5 Hz, 2 x AB-*H*), 3.40 (q, 2H, *J* 6.6 Hz, NHCONH-C*H₂*), 2.56 (q, 2H, *J* 7.5 Hz, CONH-C*H₂*), 2.46 (td, 2H, *J* 7.1, 2.3 Hz, (Pyr)CC-C*H₂*), 2.40 (t, 2H, *J* 6.8 Hz, (Nap)CC-C*H₂*), 1.82 (q, 2H, *J* 6.6, 5.9 Hz, C*H₂*), 1.76 – 1.57 (m, 6H, 3 x C*H₂*), 1.57 – 1.37 (m, 8H, 4 x C*H₂*); ^{13}C NMR (125 MHz, chloroform-d₃) δ 172.7, 156.5, 154.7, 154.6, 154.4, 154.4, 153.2, 152.8, 152.3, 150.4, 147.1, 139.6, 139.5, 136.9, 136.9, 136.8, 136.7, 126.3, 126.3, 126.1, 116.8, 116.8, 115.9, 115.8, 115.7, 115.7, 115.3, 115.3, 115.2, 95.9, 94.8, 78.9, 78.5, 39.8, 37.6, 29.8, 28.7, 28.7, 28.4, 28.1, 28.1, 26.5, 25.1, 19.5, 19.3; ^{19}F NMR (375 MHz, chloroform-*d*) δ -119.7 (dd), -120.8 (d); Rf 0.39 (ethyl acetate); IR ν_{max} (solid state) = 3271.2, 3140.0, 3050.6, 2928.1, 2855.0, 2229.5, 1662.0 cm^{-1}; ESI-HRMS *m/z* found 777.3398 $[M + H]^+$ $C_{43}H_{41}F_4N_8O_2$ requires 777.3283.

1-(Oct-7-yn-1-yl)-*N*-(4-(1-ethylpentyl)-1,6-dihydro-6-oxo-2-pyrimidinyl))urea 12

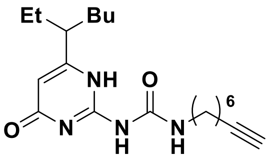

Triethylamine (0.17 mL, 1.20 mmol, 1 eq) and diphenylphosphoryl azide (0.24 mL, 1.20 mmol, 1 eq) were added to a solution of 8-nonynoic acid **5** (0.20 g. 1.30 mmol, 1 eq) in acetonitrile (6 mL). After stirring for 2 h at 50 °C, **11** (0.25 g, 1.20 mmol, 1 eq) was added, and the solution was heated to reflux for 5 h. The solution was concentrated, and the residue purified by column chromatography (SiO_2, 1:1 hexane: ethyl acetate) to give the title compound **12** as a white solid (0.30 g, 0.83 mmol, 69 %). ^{1}H NMR (500 MHz, chloroform-*d*) δ 13.29 (s, 1H, N*H*), 11.95 (s, 1H, N*H*), 10.25 (s, 1H, N*H*), 5.86 (s, 1H, Ar*H*), 3.30 (td, 2H, *J* 7.5, 5.4 Hz, HNCONH-C*H₂*), 2.34 (m, 1H, EtBu-C*H*), 2.22 (td, 2H, *J* 7.0, 2.7 Hz, HCC-C*H₂*), 1.96 (t, 1H, *J* 2.7 Hz, CC*H*), 1.76 – 1.61 (m, 6H, 3 x C*H₂*), 1.56 (d, 2H, *J* 7.0 Hz, C*H₂*), 1.52 – 1.45 (m, 2H, C*H₂*), 1.44 – 1.23 (m, 6H, 3 x C*H₂*), 0.93 (dt, 6H, *J* 11.5, 7.3 Hz, 2 x C*H₃*); ^{13}C NMR (100 MHz, chloroform-*d*) δ 167.2, 155.5, 147.2, 139.3, 106.2, 84.1, 68.1, 45.4, 40.0, 32.9, 29.3, 29.2, 28.4, 28.2, 26.6, 26.5, 22.5, 18.3, 13.9, 11.7; Rf (0.77, 1:1 hexane: ethyl acetate); IR ν_{max} (solid state) = 3246.7, 2935.6, 2496.7, 1693.6 cm^{-1}; ESI-HRMS *m/z* found 361.2598 $[M + H]^+$ $C_{20}H_{33}N_4O_2$ requires 361.2598.

1-(8-(4-((4-Bromo -2,6- difluorophenyl) diazenyl)- 3,5-difluorophenyl) oct7-yn-1-yl)-*N*-(4-(1-ethylpentyl)-1,6-dihydro-6-oxo-2-pyrimidinyl))urea) 15

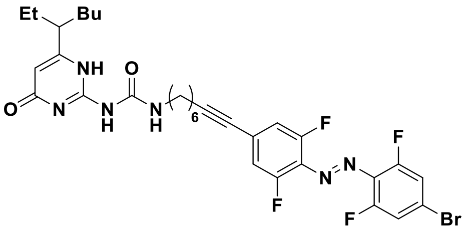

1-(4-Bromo-2,6-difluorophenyl)-2-(2,6-difluoro-4-iodophenyl)diazene **3** (0.60 g, 1.39 mmol, 1.0 eq), bis(triphenylphosphine)palladium(II) dichloride (2 mol %), copper iodide (5 mol %) and 1-(Oct-7-yn-1-yl)-*N*-(4-(1-ethylpentyl)-1,6-dihydro-6-oxo-2-pyrimidinyl))urea **12** (0.50 g, 1.39 mmol, 1.0 eq) were added to an oven dried flask. The flask was evacuated and back-filled with nitrogen three times, before anhydrous tetrahydrofuran (20 mL) was added. Anhydrous triethylamine (0.97 mL, 6.95 mmol, 5.0 eq) was prepared via three freeze, pump thaw cycles then added to the reaction mixture. The solution was stirred under nitrogen at room temperature for 16 h. The solvent was removed and the crude red solid purified via column chromatography (SiO_2, dichloromethane → dichloromethane: methanol (97:3)) to afford the title compound **15** as a red oil (0.58 g, 0.84 mmol, 60 %). ^{1}H NMR (500 MHz, chloroform-*d*) δ 13.24 (s, 1H, N*H*), 11.91 (s, 1H, N*H*), 10.22 (s, 1H, N*H*), 7.28 – 7.25 (m, 2H, 2 x AB-*H*), 7.21 – 6.89 (m, 2H, 2 x AB-*H*), 5.82 (s, 1H, Ar-*H*), 3.27 (m, 2H, NHCONH-C*H*₂), 2.43 (t, 2H, *J* 7.0 Hz, CC-C*H*₂), 2.30 (m, 1H, EtBu-C*H*), 1.64 (m, 6H, 3 x C*H*₂), 1.51 (m, 4H, 2 x C*H*₂), 1.47 – 1.37 (m, 2H, C*H*₂), 1.27 (m, 4H, 2 x C*H*₂), 0.88 (m, 6H, 3 x C*H*₃); ^{13}C NMR (125 MHz, chloroform-*d*) δ 173.1, 157.5 – 155.2 (m), 155.6, 154.8, 154.5 (d), 152.4, 148.8, 135.7, 131.0 (d), 124.0, 116.7 (d), 116.2 – 115.0 (m), 106.2, 96.3, 78.9, 45.4, 40.0, 32.9, 29.3, 28.6, 28.2, 27.2 – 25.3 (m), 22.5, 19.5, 13.9, 11.7; ^{19}F NMR (375 MHz,

chloroform-*d*) δ -118.9 (d), -120.6 (d); Rf 0.50 (95:5 dichloromethane: methanol); IR ν_{max} (solid state) = 3106.8, 2872.2, 2234.3, 1725.0, 1654.44, 1461.3 cm^{-1}; ESI-HRMS *m/z* found 693.2035 [M + H]$^+$ $C_{32}H_{36}BrF_4N_6O_2$ requires 693.1993.

1,2-Bis(2,6-difluoro-4-1-(Oct-7-yn-1-yl)-*N*-(4-(1-ethylpentyl)-1,6-dihydro-6-oxo-2-pyrimidinyl))urea)diazene Foldamer II

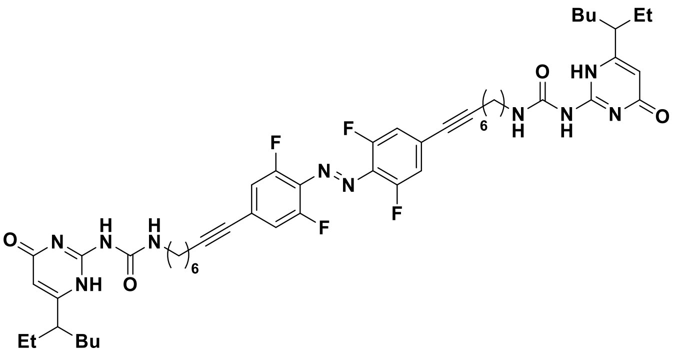

1-(8-(4-((4-Bromo -2,6- difluorophenyl) diazenyl)- 3,5-difluorophenyl) oct7-yn-1-yl)-*N*-(4-(1-ethylpentyl)-1,6-dihydro-6-oxo-2-pyrimidinyl))urea) **15** (0.05 g, 0.07 mmol, 1.0 eq), bis(triphenylphosphine)palladium(II) dichloride (10 mol%), copper iodide (25 mol%) and 1-(oct-7-yn-1-yl)-*N*-(4-(1-ethylpentyl)-1,6-dihydro-6-oxo-2-pyrimidinyl))urea **12** (0.04 g, 0.11 mmol, 1.5 eq) were added to an oven dried flask. The flask was evacuated and back-filled with nitrogen three times and anhydrous triethylamine (5 mL), prepared via three freeze, pump thaw cycles, was added to the reaction mixture. The solution was stirred under nitrogen at 60 °C for 16 h. Upon cooling to room temperature, the solvent was removed and the crude red solid suspended in a 1:1 mixture of dichloromethane and ethyl acetate. The reaction mixture was filtered through a plug of silica then concentrated. The residue was further purified via column chromatography (SiO_2, 97:2:1 dichloromethane: methanol: acetic acid) to provide the foldamer **II** as red oil (0.01 g, 0.01 mmol, 13 %). (*E*-isomer) - ^{1}H NMR (500 MHz, chloroform-*d*) δ 13.17 (s, 2H, 2 x N*H*), 11.84 (s, 2H, 2 x N*H*), 10.15 (s, 2H, 2 x N*H*), 7.06 – 6.89 (m, 3H, 3 x AB-*H*), 6.85 – 6.67 (m, 1H, AB-*H*), 5.75 (s, 2H, 2 x Ar-*H*), 3.19 (q, *J* 6.6 Hz, 4H, 2 x NHCONH-C*H₂*), 2.40 – 2.26 (m, 4H, 2 x CC-C*H₂*), 2.16 (q, *J* 7.7, 7.2 Hz, 2H, 2 x EtBu-C*H*), 1.35 (m, 4H, 2 x C*H₂*), 1.20 (m, 12H, 6 x C*H₂*), 0.80 (dd, *J* 8.5, 5.1 Hz, 12H, 4 x C*H₃*); (*Z*-isomer) - ^{1}H NMR (500 MHz, chloroform-*d*) δ 13.14 (s, 2H, 2 x N*H*), 11.85 (s, 2H, 2 x N*H*), 10.11 (s, 2H, 2 x N*H*), 7.02 – 6.92 (m, 3H. 3 x AB-*H*), 6.76 (m, 2H, AB-*H*), 5.74 (s, 2H, 2 x Ar-*H*), 3.26 – 3.13 (m, 4H, 2 x NHCONH-C*H₂*), 2.40 – 2.25 (m, 4H, 2 x CC-C*H₂*), 2.22 – 2.14 (m, 2H, 2 x EtBu-C*H*), 1.37 – 1.34 (m, 4H, 2 x C*H₂*), 1.18 (m, 12H, 6 x C*H₂*), 0.83 – 0.77 (m, 12H, 4 x C*H₃*); ^{13}C NMR (125 MHz, chloroform-*d*) δ 173.2, 156.7, 156.5, 156.5, 155.5, 154.9, 154.5, 154.4, 131.2, 131.2, 131.1, 128.0, 127.9, 116.8, 116.6, 115.8, 115.8, 115.6, 115.2, 114.5, 106.2, 106.1, 96.6, 95.9, 94.9, 78.9, 45.4, 40.0, 32.9, 29.7, 29.3, 28.6, 28.3, 26.6, 26.5, 22.5, 19.5, 19.3, 13.9, 11.7; ^{19}F NMR (375 MHz, chloroform-*d*) δ -119.3 (d), -127.0 (d). Rf 0.59 (95:5 DCM: MeOH); IR ν_{max} (solid state) = 3218.1, 3031.9, 2858.7, 2234.1, 1694.9 cm cm^{-1}; ESI-HRMS *m/z* found 971.5277 [M + H]$^+$ $C_{52}H_{67}F_4N_{10}O_4$ requires 971.5238.

7-Hydroxy-1,8-naphthyridin-2-amine 18

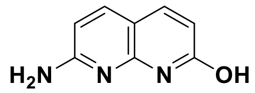

Procedure adapted from literature.[7] To, 2,6-diaminopyridine (3.34g, 30.6 mmol, 1.0 eq) and D,L-malic acid (4.51 g, 33.7 mmol, 1.1 eq), concentrated sulphuric acid (20 mL) was added dropwise at 0 °C. The reaction mixture was heated to 110 °C for 4 h. Upon cooling to 0 °C, aqueous ammonia solution was added dropwise until pH 9 was reached. The resulting brown precipitate was collected by vacuum filtration then washed with water and diethyl ether to afford the title compound as a brown powder (3.58 g, 22.4 mmol, 73 %). ^{1}H NMR (500 MHz, dimethyl sulfoxide-*d*$_6$) δ 11.51 (br s, 1H, O*H*), 7.54 (dd, 2H, *J* 8.5, 9.3 Hz, 2 x Nap-*H*), 6.71 (s, 2H, N*H$_2$*), 6.24 (d, 1H, *J* 8.5 Hz, Nap-*H*), 6.01 (d, 1H, *J* 9.3 Hz, Nap-*H*);^{13}C NMR (125 MHz, dimethyl sulfoxide-*d*$_6$) δ 164.0, 160.9, 150.9, 139.9, 137.6, 115.4, 105.4, 105.4, 40.6, 40.5, 40.4, 40.3, 40.3, 40.2, 40.0, 39.8, 39.7, 39.5; Rf 0.59 (9:1 dichloromethane: methanol); IR ν_{max} (solid state) = 3353.3, 3154.6, 3044.2, 1618.7 cm^{-1}; ESI-HRMS *m/z* found 162.0651 [M + H]$^+$ $C_8H_8N_3O$ requires 162.0662.

7-(2-ethyl-hexanoyl)-amino-8H-(7-oxo-[1,8]-naphthyridine-2-yl) 19

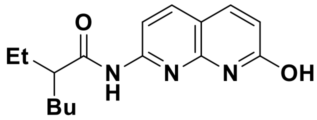

Procedure adapted from literature.[7] 2-Ethylhexnoyl chloride (2.6 mL, 15 mmol, 1.2 eq) was slowly added to a solution of **18** (2.0 g, 12.5 mmol, 1 eq) in dry pyridine (40 mL). The reaction mixture was heated to 110 °C for 22 h. Upon cooling to room temperature, the solvent was removed then the residue dissolved in dichloromethane (50 mL) then washed with 1M hydrochloric acid (2x 25 mL), water (2x 25 mL), saturated sodium bicarbonate solution (2 x 25 mL). The organic layer was dried with sodium sulfate, filtered and concentrated. The title compound was crystallised from acetone to give a cream solid (1.3 g, 4.6 mmol, 37 %). ^{1}H NMR (400 MHz, chloroform-*d*) δ 12.88 (br s, 1H, O*H*), 11.75 (s, 1H, N*H)*, 8.46 (d, 1H, *J* 8.7 Hz, Nap-*H*), 7.94 (d, *J* 8.7 Hz, 1H, Nap-*H*), 7.76 (d, 1H, *J* 9.4 Hz, Nap-*H*), 6.65 (d, 1H, *J* 9.4 Hz, Nap-*H*), 2.83 (tt, 1H, *J* 9.1, 5.1 Hz, EtBu-C*H*), 1.82 – 1.68 (m, 2H, C*H$_2$*), 1.66 – 1.47 (m, 2H, C*H$_2$*), 1.34 (m, 4H, 2 x C*H$_2$*), 0.97 (t, 3H, *J* 7.4 Hz, C*H$_3$*), 0.86 (t, 3H, *J* 7.1 Hz, C*H$_3$*); ^{13}C NMR (100 MHz, chloroform-*d*) δ 177.7, 165.2, 154.2, 148.7, 139.7, 139.0, 119.9, 111.3, 110.9, 48.6, 32.5, 29.7, 26.2, 22.9, 14.0, 11.9; Rf 0.74 (9:1 dichloromethane: methanol); IR ν_{max} (solid state) = 3171.5, 2998.9, 2871.5, 1700.3, 1662.3 cm^{-1}; ESI-HRMS *m/z* found 288.1708 [M + H]$^+$ $C_{16}H_{22}N_3O_2$ requires 288.1707.

7-(2-ethyl-hexanoyl)-amino-7-chloro-[1,8]-naphthyridine-2-yl 20

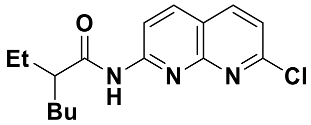

Procedure adapted from literature.[7] **19** (0.4 g, 1.4 mmol, 1.0 eq) was dissolved in Phosphorus oxychloride (10 mL) and the solution was heated to 95 °C for 4 h. Upon cooling to room temperature, the reaction mixture was slowly poured onto ice cold water. The resulting mixture was extracted into dichloromethane (4 x 20 mL), the extracts were combined and washed with saturated sodium

bicarbonate solution (3 x 20 mL), water (3 x 20 mL) and brine (20 mL). The organic extracts were dried with sodium sulfate, filtered and concentrated to afford the title compound as a yellow crystalline solid (0.32 g, 1.05 mmol, 75%). ^{1}H NMR (500 MHz, chloroform-*d*) δ 8.61 (d, 1H, J 8.8 Hz, Nap-*H*), 8.31 (s, 1H, N*H*), 8.21 (d, 1H, J 8.8 Hz, Nap-*H*), 8.08 (d, 1H, J 8.4 Hz, Nap-*H*), 7.41 (d, 1H, J 7.7 Hz, Nap-*H*), 2.33 – 2.17 (m, 1H, EtBu-C*H*), 1.81 – 1.68 (m, 2H, C*H₂*), 1.66 – 1.54 (m, 2H, C*H₂*), 1.33 (s, 4H, 2 x C*H₂*), 0.98 (t, 3H, J 7.4 Hz, C*H₃*), 0.87 (m, 3H, C*H₃*); ^{13}C NMR (125 MHz, chloroform-*d*) δ 175.6, 154.5 154.1, 153.8, 139.2, 138.8, 122.1, 119.2, 115.3, 51.1, 32.4, 29.7, 26.0, 22.8, 13.9, 12.0; Rf 0.70 (1:1 ethyl acetate: Hexane); IR ν_{max} (solid state) = 3176.2, 3128.2, 2958.5, 2857.9, 1693.9 cm^{-1}; ESI-HRMS *m/z* found 306.1362 $[M + H]^+$ $C_{16}H_{21}ClN_3O$ requires 306.1368.

N-(7-Amino-1,8-naphthyridin-2-yl)-2-ethylhexanamide 21

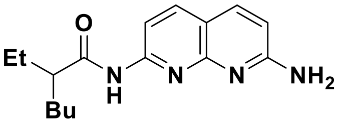

20 (0.5 g, 1.64 mmol, 1.0 eq), tert-butylcarbamate (0.30 g, 2.62 mmol, 1.6 eq), potassium carbonate (0.45 g, 3.27 mmol, 2.0 eq), palladium(II) acetate (10 mg, 0.05 mmol, 0.03 eq) and xantphos (60 mg, 0.10 mmol, 0.06 eq) were suspended in 1,4-dioxane (10 mL). The reaction mixture was degassed via three freeze, pump, thaw cycles, then heated to 100 °C for 48 h. Upon cooling to room temperature, the reaction mixture was diluted in ethyl acetate and filtered through celite. The solvent was evaporated in vacuo and the crude product was purified via column chromatography (SiO_2, ethyl acetate → ethyl acetate: methanol (9:1)) to afford the title compound as a white solid (0.26 g, 0.93 mmol, 57 %). ^{1}H NMR (500 MHz, chloroform-*d*) δ 8.25 (d, 1H, *J* 8.6 Hz, Nap-*H*), 8.06 (s, 1H, N*H*), 7.96 (d, 1H, *J* 8.6 Hz, Nap-*H*), 7.82 (d, 1H, *J* 8.6 Hz, Nap-*H*), 6.67 (d, 1H, *J* 8.6 Hz, Nap-*H*), 4.92 (s, 2H, NH_2), 2.19 (ddd, 1H, *J* 14.2, 9.1, 5.2 Hz, EtBu-*H*), 1.78 – 1.66 (m, 2H, C*H₂*), 1.64 – 1.57 (m, 2H, C*H₂*), 1.36 – 1.29 (m, 4H, 2 x C*H₂*), 0.97 (t, 3H, *J* 7.4 Hz, C*H₃*), 0.89 – 0.86 (m, 3H, C*H₃*); ^{13}C NMR (125 MHz, chloroform-*d*) δ 175.2, 164.8, 159.8, 153.0, 138.7, 137.9, 115.3, 110.6, 110.4, 51.2, 32.6, 29.8, 26.2, 22.8, 13.9, 12.1; Rf 0.31 (ethyl acetate); IR ν_{max} (solid state) = 3444.7, 3292.2, 3122.6, 2868.7, 1698.4 cm^{-1}; ESI-HRMS *m/z* found 287.1850 $[M + H]^+$ $C_{16}H_{23}N_4O$ requires 287.1866.

N-((1,8-Naphthyridin-2-yl)-2-ethylhexanamide)non-8-ynamide 22

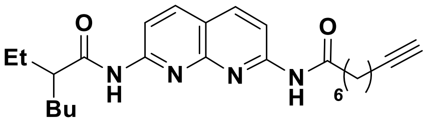

EDC (14 mg. 0.09 mmol, 1.2 eq) was added to a solution of **5** (12 mg, 0.08 mmol, 1.0 eq) and DMAP (15 mg, 0.12 mmol, 1.5 eq) in chloroform (5 mL). After stirring for 30 min, **21** (22 mg, 0.08 mmol, 1.0 eq) was added, and the solution was stirred at 60 °C for 16 h. Upon cooling to room temperature, the reaction mixture diluted with chloroform (5 mL) and washed with 1M hydrochloric acid (2 x 5 mL), saturated sodium bicarbonate solution (2 x 5 mL) and brine (5 mL). The organic layer was collected, dried with sodium sulfate, filtered and concentrated. The residue was suspended in hexane and filtered through silica and eluted with ethyl acetate: hexane (1:1). The solvent was removed to afford the title compound as a colourless solid (23 mg, 0.06 mmol, 70 %). ^{1}H NMR (500 MHz, chloroform-*d*) δ 8.47 (d, 1H, *J* 8.8 Hz, Nap-*H*), 8.43 (d, 1H, *J* 8.8 Hz, Nap-*H*), 8.17 (br s, 2H, 2 x N*H*), 8.14 (dt, 2H, *J* 8.8, 0.6 Hz, 2 x Nap-*H*), 2.47 (t, 2H, *J* 7.5 Hz, NHCO-C*H₂*), 2.28 – 2.21 (m, 1H, EtBu-C*H*), 2.21 – 2.17 (m, 2H,

HCC-CH_2), 1.94 (t, 1H, *J* 2.7 Hz, CC*H*), 1.81 – 1.72 (m, 4H, 2 x CH_2), 1.66 – 1.57 (m, 4H, 2 x CH_2), 1.45 (m, 2H, CH_2), 1.39 – 1.27 (m, 6H, 3 x CH_2), 0.98 (t, *J* 7.5 Hz, 3H, CH_3), 0.90 – 0.87 (m, 3H, CH_3); ^{13}C NMR (125 MHz, chloroform-*d*) δ 175.21, 171.9, 153.6, 153.6, 152.2, 138.9, 138.9, 118.3, 113.4, 113.2, 84.3, 68.2, 51.0, 37.8, 32.4, 31.5, 29.7, 29.6, 28.5, 28.3, 28.1, 26.0, 25.0, 22.6, 18.2, 13.8, 11.9; Rf 0.28 (1:1 ethyl acetate: hexane); IR ν_{max} (solid state) = 3311.0, 3136.7, 2929.9, 2857.2, 2117.1, 1773.3, 1694.1 cm^{-1}; ESI-HRMS *m/z* found 423.2756 [M + H] $^+$ $C_{25}H_{35}N_4O_2$ requires 423.2755.

9- (4- ((2,6-Difluoro - 4 - (oct-7-yn-1-yl) -*N*- (4-(1-ethylpentyl) - 1,6 - dihydro-6-oxo -2-pyrimidinyl)) urea) phenyl) diazenyl) - 3,5 - difluorophenyl) -*N*- ((1,8-naphthyridin-2-yl)-2-ethylhexanamide)non-8-ynamide Foldamer III

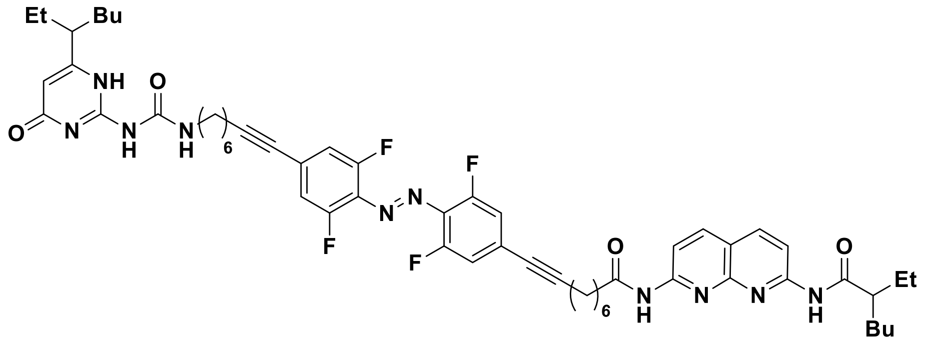

15 (0.23 g, 0.33 mmol, 1.0 eq), bis(triphenylphosphine)palladium(II) dichloride (2 mol%), copper iodide (5 mol%) and **22** (0.14 g, 0.33 mmol, 1.0 eq) were added to an oven dried flask. The flask was evacuated and back-filled with nitrogen three times, before anhydrous triethylamine (10 mL), prepared *via* three freeze, pump thaw cycles, was added to the reaction mixture. The solution was stirred under nitrogen at 60 °C for 16 h. The solvent was removed, the resultant crude solid was resuspended in dichloromethane then filtered through celite. The reaction mixture was concentrated, then triturated with methanol. The solids were collected by vacuum filtration the purified by column chromatography (dichloromethane: methanol: triethyl amine mixtures). The title compound was afforded as a dark red oil (30 mg, 0.03 mmol, 10 %). (*E*-isomer) - ^{1}H NMR (500 MHz, chloroform-*d*) δ 13.84 (s, 1H, N*H*), 11.84 (br s, 2H, 2 x N*H*), 11.31 (s, 1H, N*H*), 9.81 (s, 1H, N*H*), 8.46 (dd, 2H, *J* 14.5, 8.8 Hz, 2 x Nap-*H*), 8.04 (d, 2H, , *J* 8.8 Hz, 2 x Nap-*H*), 6.98 -6.78 (m, 4H, 4 x AB-*H*), 5.90 (s, 1H, Ar-*H*), 3.42-3.35 (m, 2H, NHCONH-CH_2), 3.19 -3.11 (m, 1H, EtBu-C*H*), 2.55 -2.49 (m, 3H, EtBu-C*H*, NHCO-CH_2), 2.37 -2.33 (m, 6H, 2 x CC-CH_2, CH_2), 2.24 -2.17 (m, 6H, 3 x CH_2), 1.67-2.46 (m, 12 H, 6 x CH_2), 1.26 -1.18 (m, 10 H, 5 x CH_2), 0.90 (t, 3H, *J* 7.3, CH_3), 0.80 (dt, 9H, *J* 18.0, 9.1 Hz, 3 x CH_3); (*Z*-isomer) - ^{1}H NMR (500 MHz, chloroform-*d*) δ 13.85 (s, 1H, N*H*), 11.60 (br s, 2H, 2 x N*H*), 11.27 (s, 1H, N*H*), 9.77 (s, 1H, N*H*), 8.47 (dd, 2H, *J* 14.5, 8.8 Hz, 2 x Nap-*H*), 8.05 (d, 2H, , *J* 8.8 Hz, 2 x Nap-*H*), 7.07-6.76 (m, 4H, 4 x AB-*H*), 5.90 (s, 1H, Ar-*H*), 3.36 (m, 2H, NHCONH-CH_2), 3.20-3.14 (m, 1H, EtBu-C*H*), 2.66-2.54 (m, 3H, EtBu-C*H*, NHCO-CH_2), 2.55-2.51 (m, 6H, 2 x CC-CH_2, CH_2), 2.38-2.34 (m, 6H, 3 x CH_2), 2.28-2.09 (m, 12 H, 6 x CH_2), 1.75-1.62 (m, 10 H, 5 x CH_2), 0.90 (t, 3H, *J* 7.3, CH_3), 0.80 (dt, 9H, *J* 18.0, 9.1 Hz, 3 x CH_3); ^{13}C NMR (125 MHz, chloroform-*d*) δ ^{13}C NMR (100 MHz, $CDCl_3$) δ 170.9, 170.6, 165.1, 157.1, 156.7, 155.4, 155.3, 155.0, 150.8, 142.8, 139.4, 133.9 (d), 131.2, 129.7, 126.6 (d), 115.8, 115.6, 115.3, 115.1 - 114.9 (m), 114.2, 106.3, 87.0, 86.9, 79.7, 48.8, 41.8, 40.0, 37.2, 33.8, 32.4, 30.8, 29.8 - 29.5 (m), 28.7, 28.6 (d), 28.2 (d), 27.4, 26.7, 26.1, 25.3, 22.8 (d), 19.5, 19.2, 14.0 (d), 12.0; ^{19}F NMR (375 MHz, chloroform-*d*) δ -119.3 (d), -127.0 (d); Rf 0.50 (95:5 DCM: MeOH); IR ν_{max} (solid state) = 3191.1, 3053.0, 2857.3, 2229.9, 1697.2 cm^{-1}; ESI-HRMS *m/z* found 1033.5455 [M + H]$^+$ $C_{57}H_{69}F_4N_{10}O_4$ requires 1033.5434.

4. Photoisomerization studies

For irradiation of foldamer samples, high-power LED system was used. Green 530 nm light was used for *E* to *Z* isomerisation and blue 405 nm light was used for *Z* to *E* isomerisation. Irradiation was carried out at room temperature, with stirring, for 10 minutes. Samples were stirred magnetically in a glass vial covered with foil. After irradiation samples were transferred to NMR tubes and immediately transferred to the NMR sample changer.

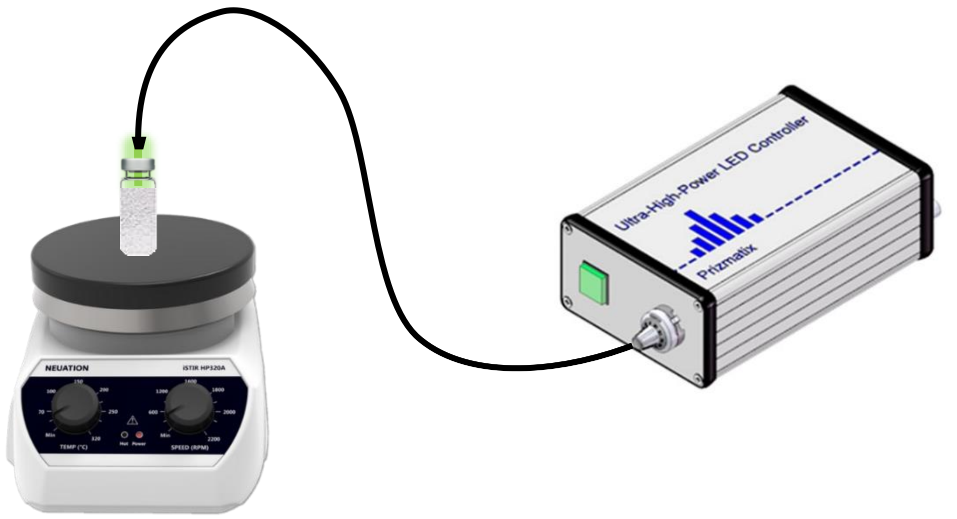

Figure S1. Representation of the set up used for irradiation of samples

5. DOSY NMR

DOSY spectra were obtained using a four-channel Bruker AV-NEO NMR spectrometer operating at 11.7 T (500 MHz ^{1}H) and equipped with 5mm TXI probe (δ = 0.002 s, Δ = 0.0999). Sample were prepared in Wilmad 500 MHz 5mm 528-PP-7 tubes using anhydrous deuterated chloroform purchased from Sigma Aldrich.

5.1 DOSY Spectra

Pyr.NAP 4 mM 405 nm

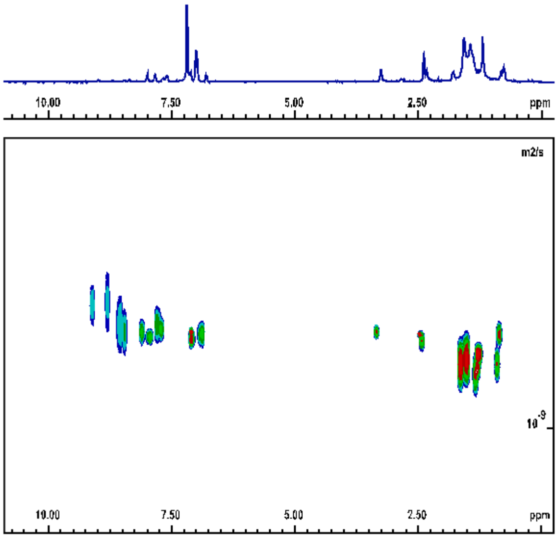

Pyr.NAP 4 mM 530 nm

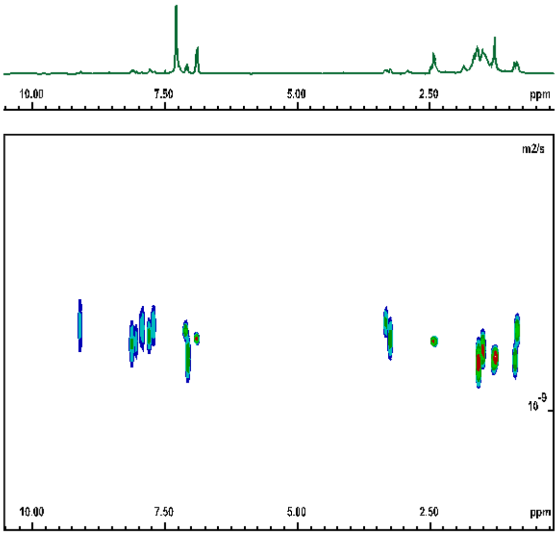

Pyr.NAP 8 mM 405 nm

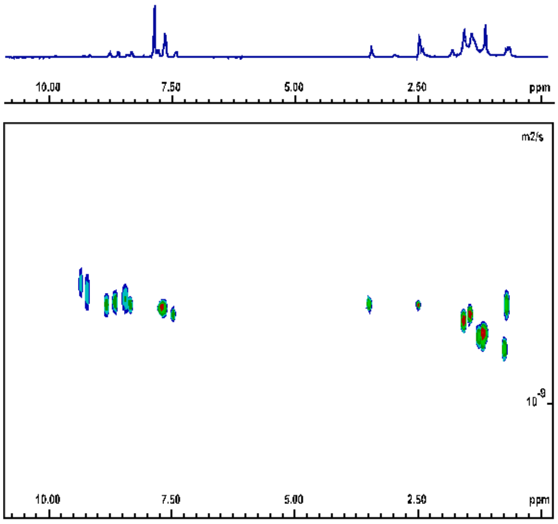

Pyr.NAP 8 mM 530 nm

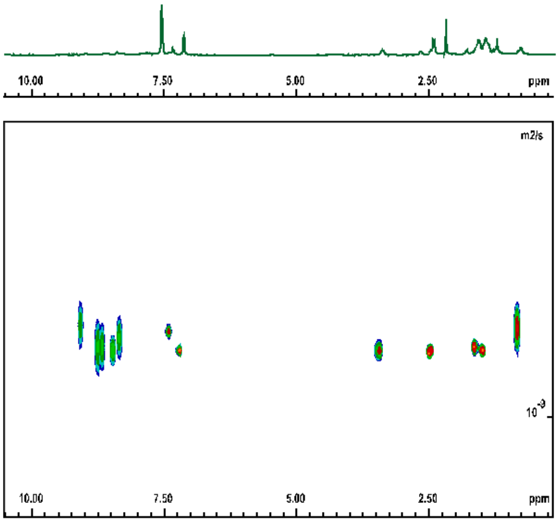

Pyr.NAP 12 mM 405 nm

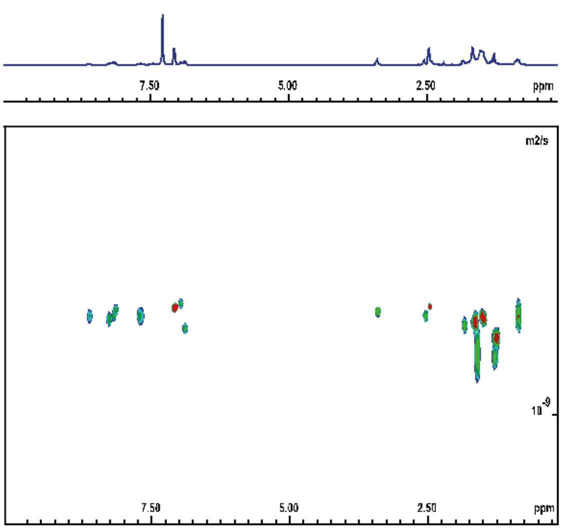

Pyr.NAP 12 mM 530 nm

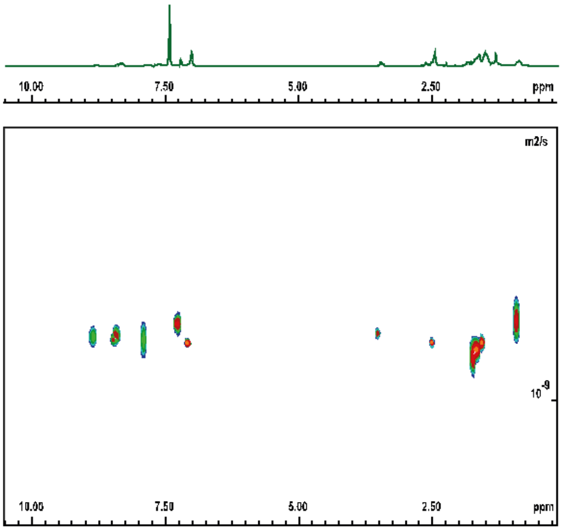

Pyr.NAP 16 mM 405 nm

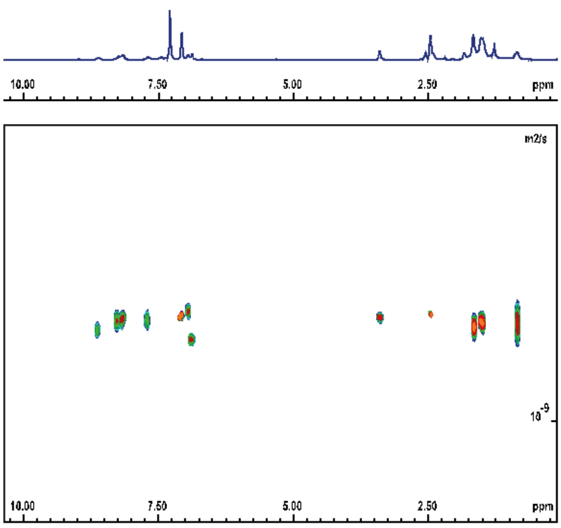

Pyr.NAP 16 mM 530 nm

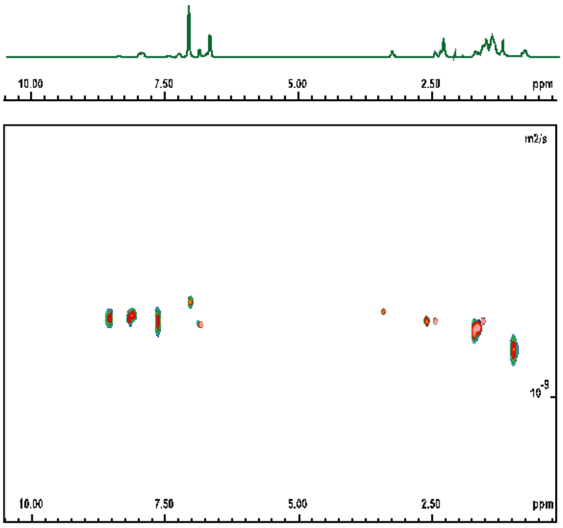

Pyr.NAP 20 mM 405 nm

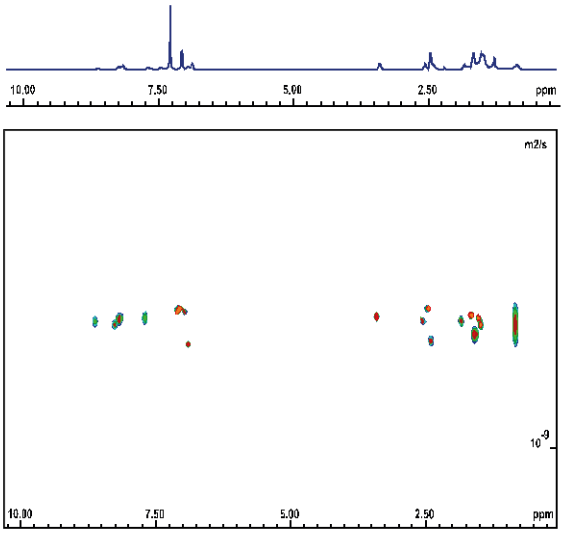

Pyr.NAP 20 mM 530 nm

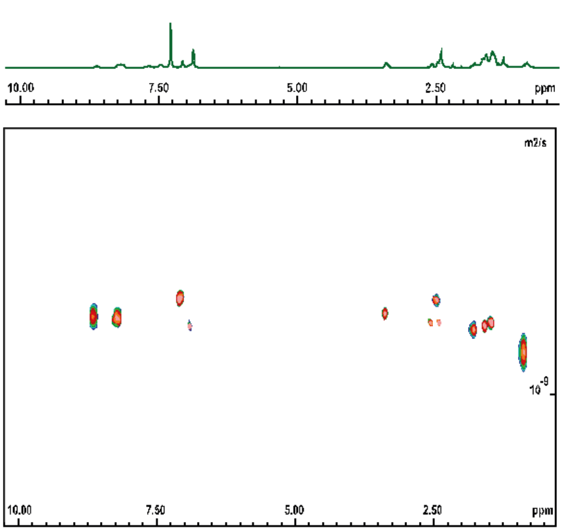

Pyr.NAP 24 mM 405 nm

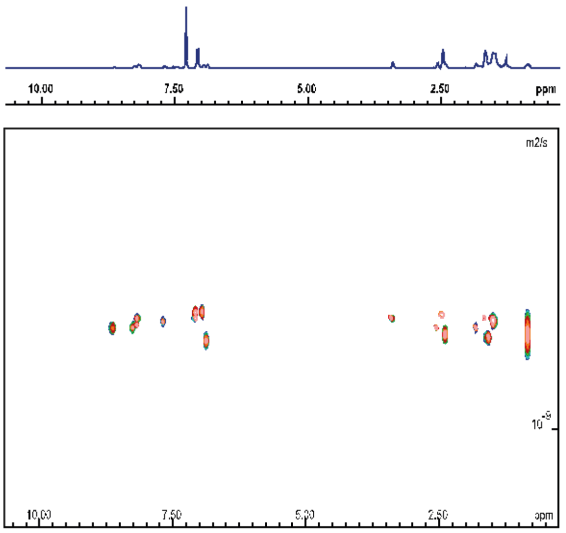

Pyr.NAP 24 mM 530 nm

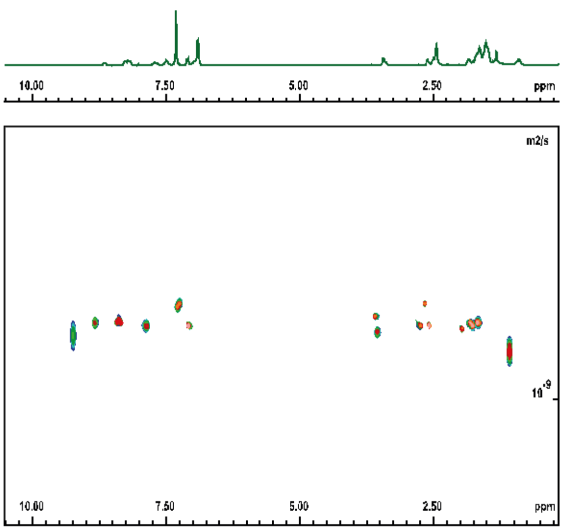

Pyr.NAP 28 mM 405 nm

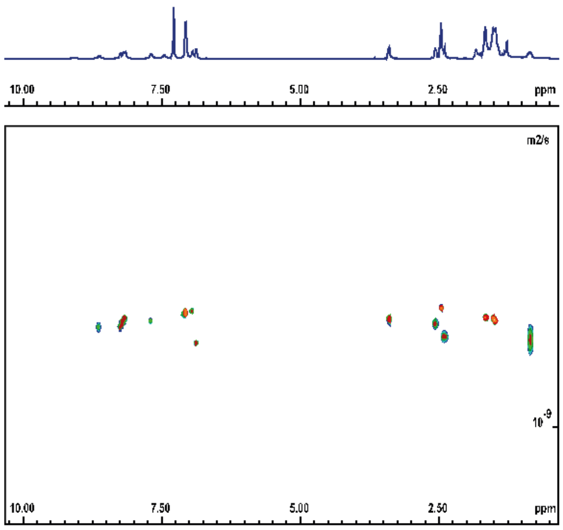

Pyr.NAP 28 mM 530 nm

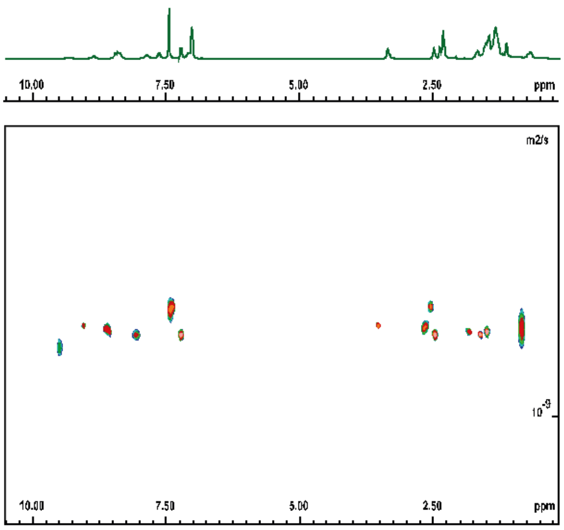

Pyr.NAP 32 mM 405 nm

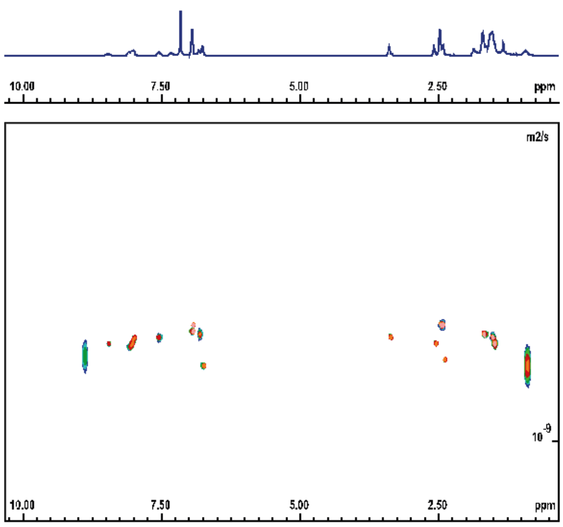

Pyr.NAP 32 mM 530 nm

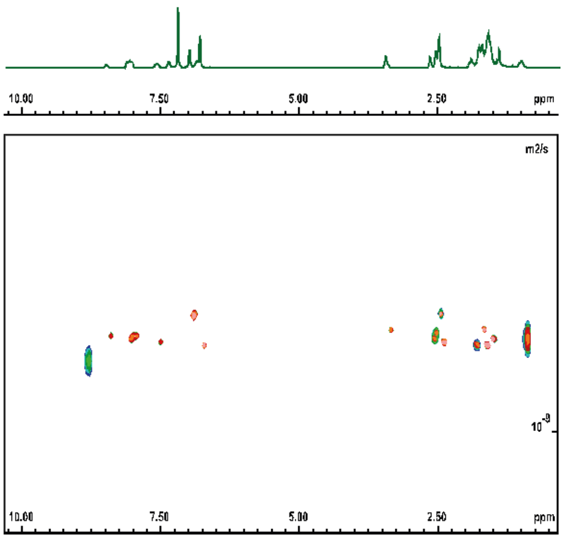

Pyr.NAP 36 mM 405 nm

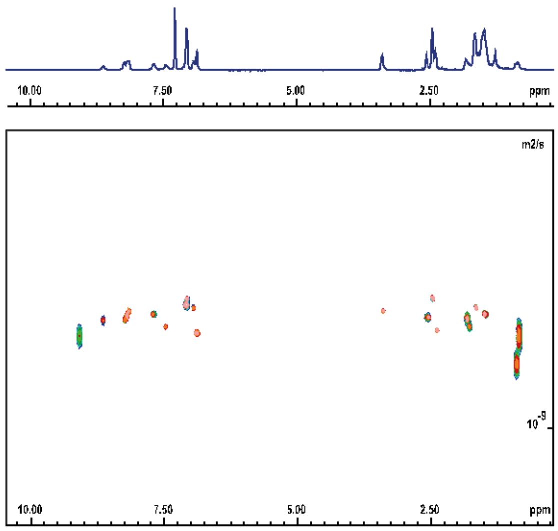

Pyr.NAP 36 mM 530 nm

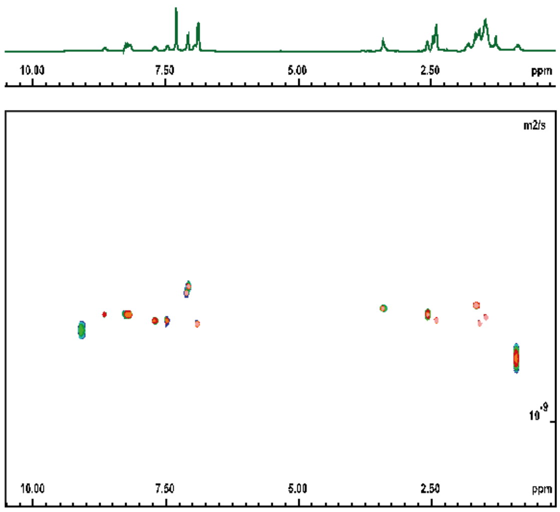

Pyr.NAP 40 mM 405 nm

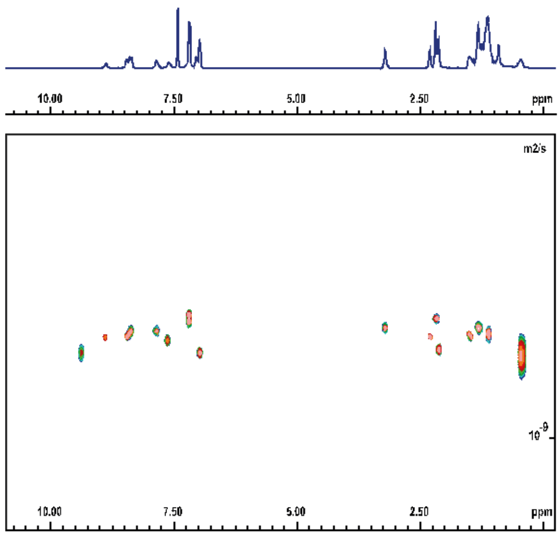

Pyr.NAP 40 mM 530 nm

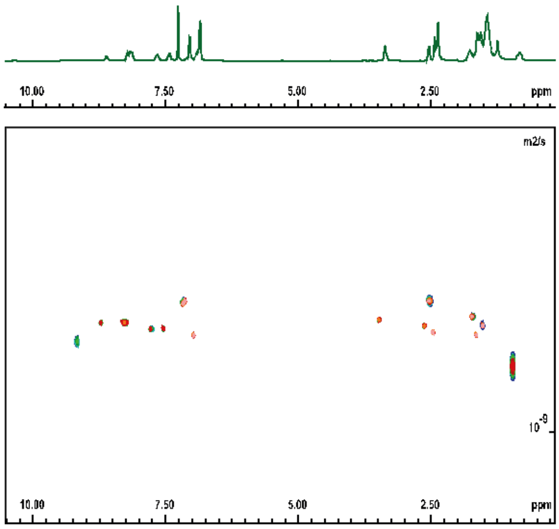

Pyr.NAP 44 mM 405 nm

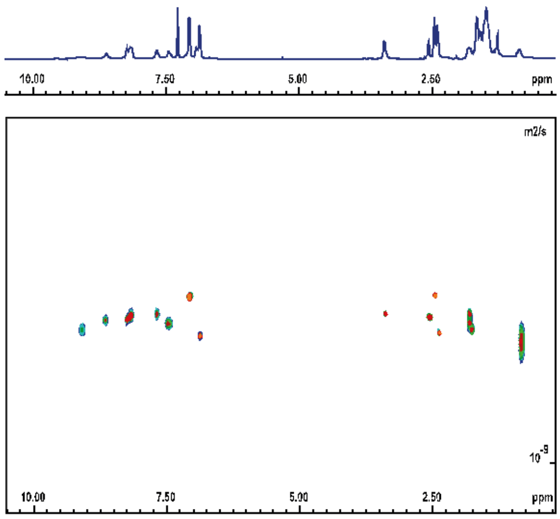

Pyr.NAP 44 mM 530 nm

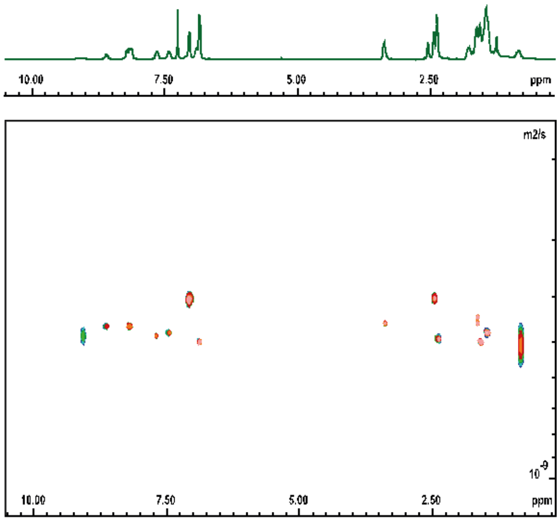

Pyr.NAP 48 mM 405 nm

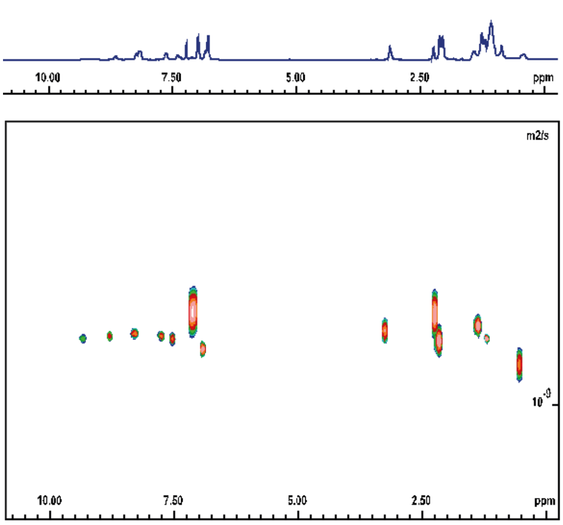

Pyr.NAP 48 mM 530 nm

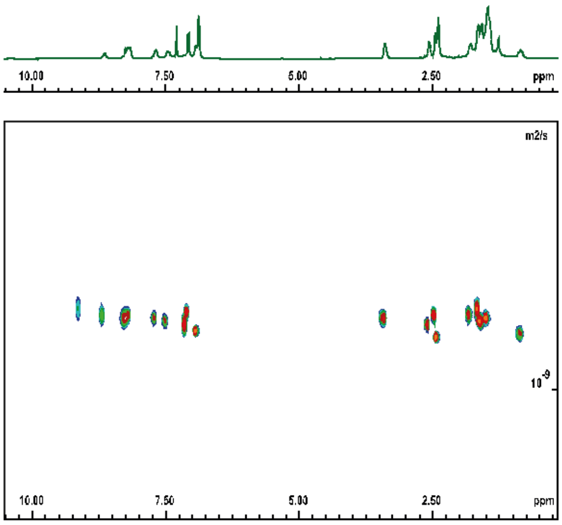

Pyr.NAP 52 mM 405 nm

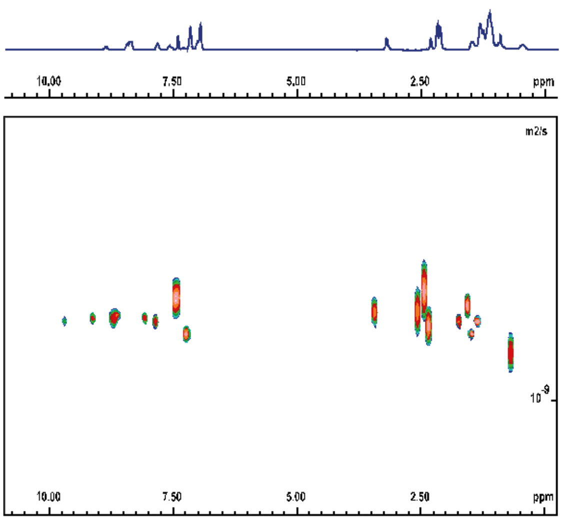

Pyr.NAP 52 mM 530 nm

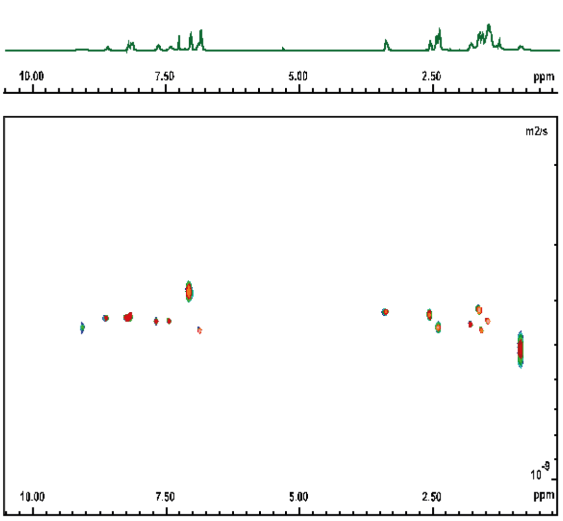

Pyr.NAP 56 mM 405 nm

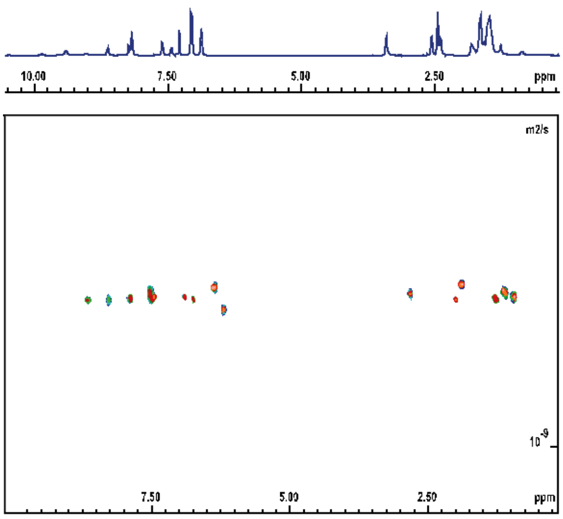

Pyr.NAP 56 mM 530 nm

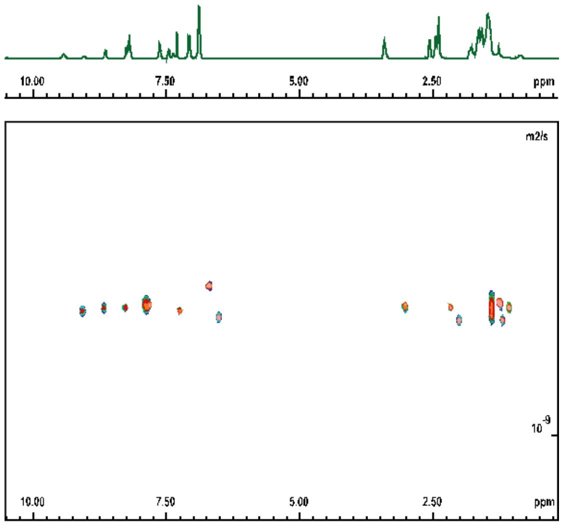

UPy.UPy 16 mM 405 nm

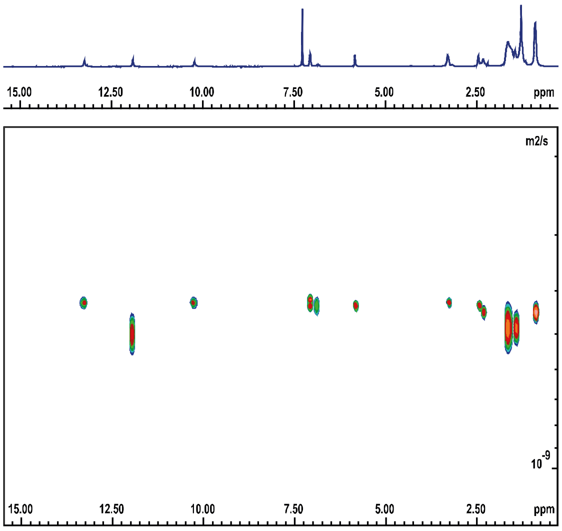

UPy.UPy 16 mM 530 nm

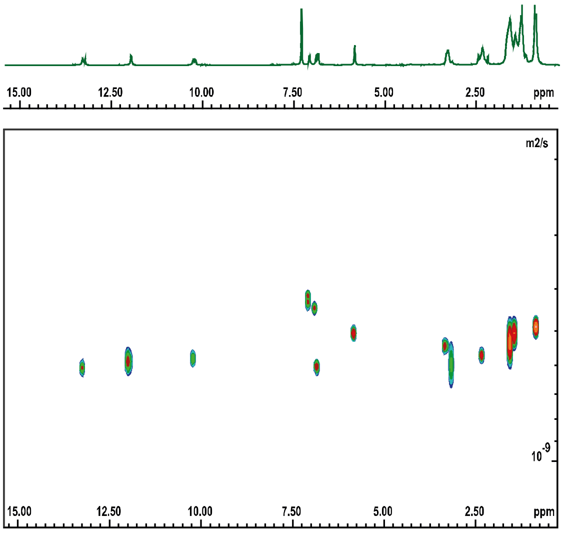

UPy.UPy 20 mM 405 nm

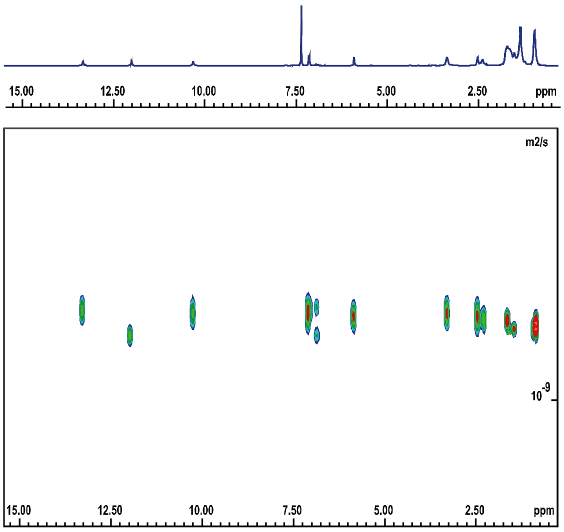

UPy.UPy 20 mM 530 nm

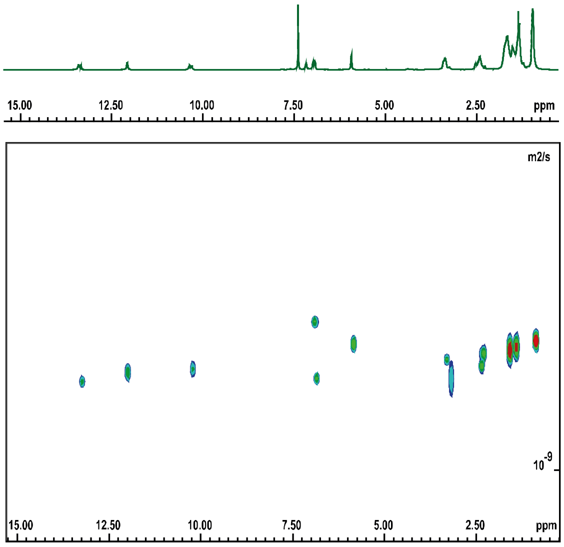

UPy.UPy 24 mM 405 nm

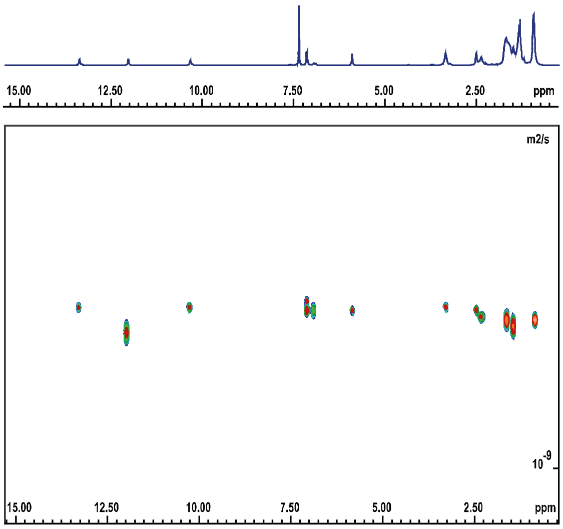

UPy.UPy 24 mM 530 nm

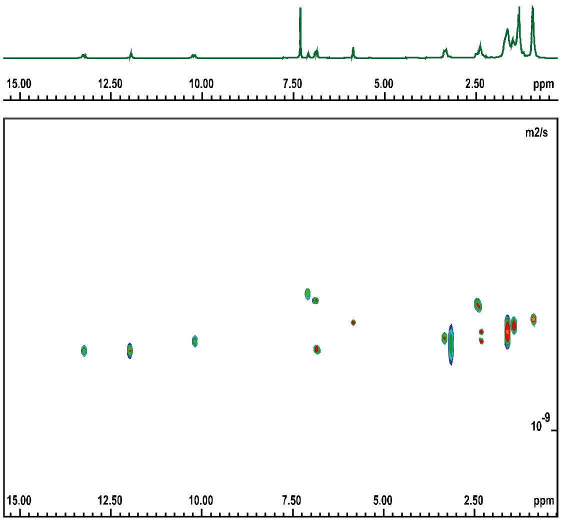

UPy.UPy 28 mM 405 nm

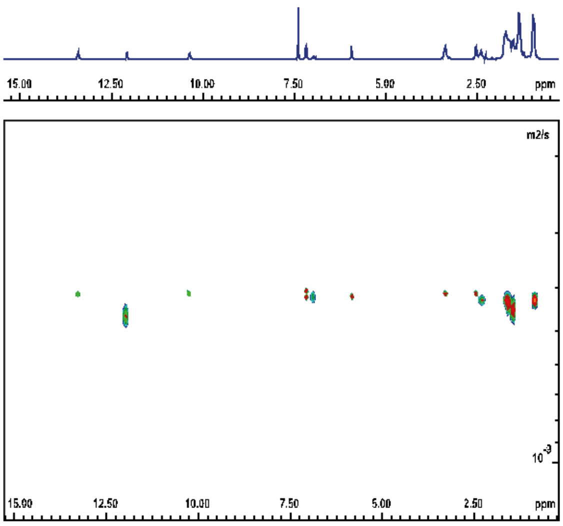

UPy.UPy 28 mM 530 nm

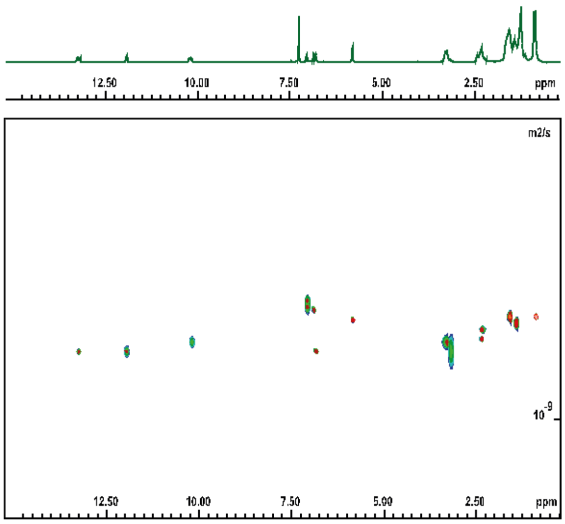

UPy.UPy 32 mM 405 nm

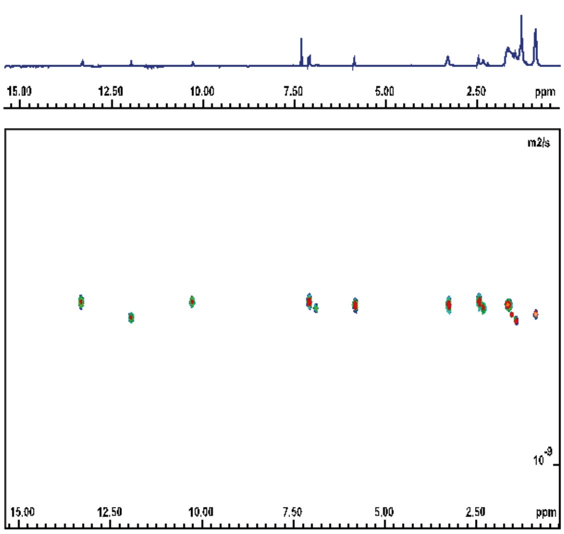

UPy.UPy 32 mM 530 nm

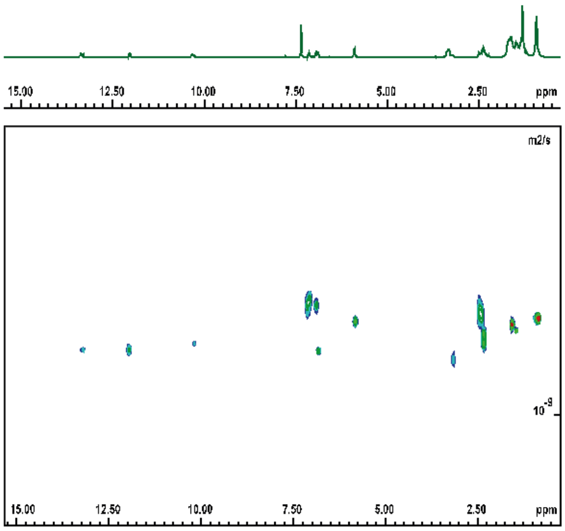

UPy.UPy 36 mM 405 nm

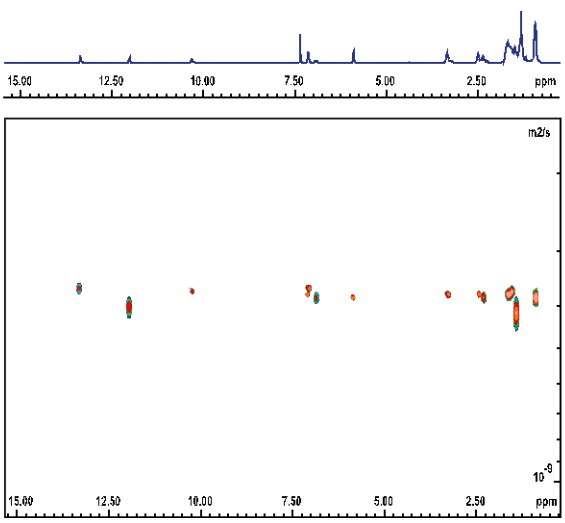

UPy.UPy 36 mM 530 nm

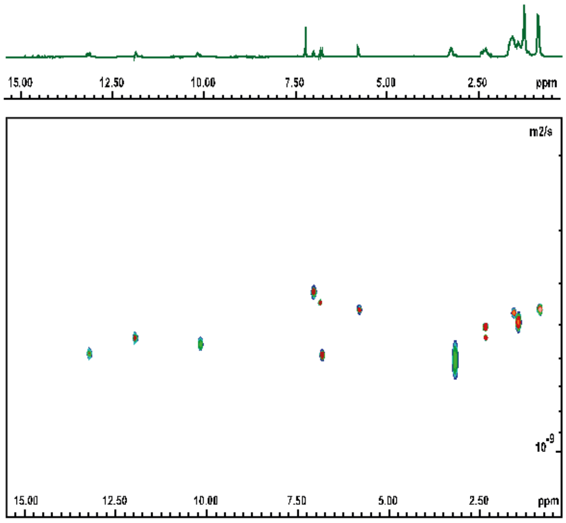

UPy.UPy 40 mM 405 nm

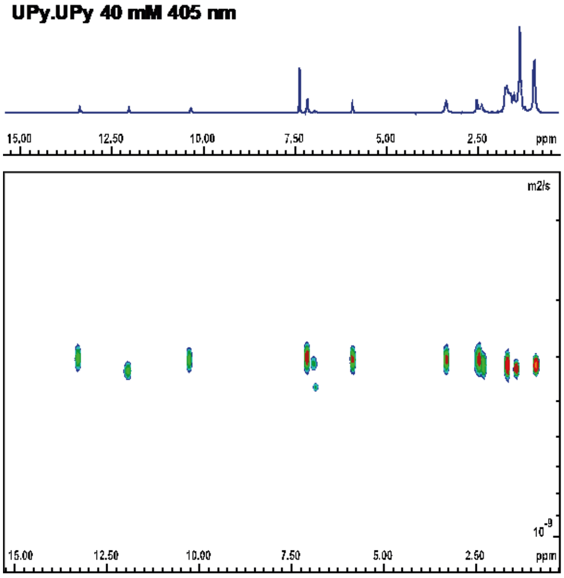

UPy.UPy 40 mM 530 nm

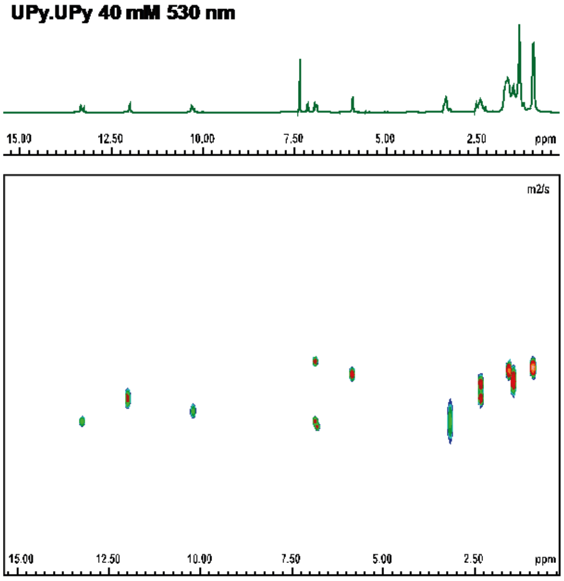

UPy.UPy 44 mM 405 nm

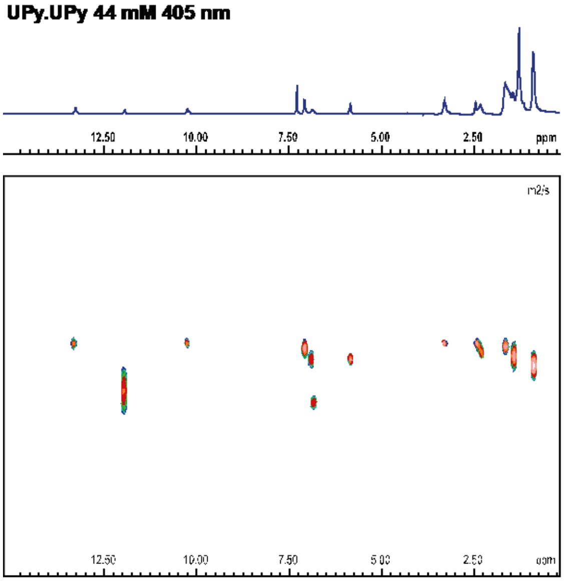

UPy.UPy 44 mM 530 nm

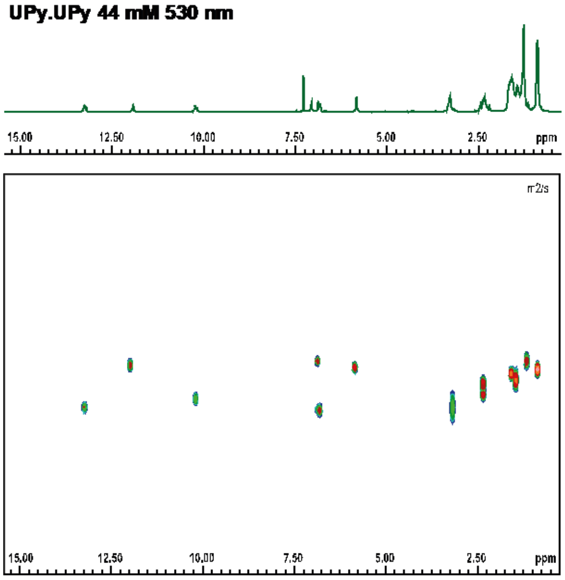

UPy.UPy 48 mM 405 nm

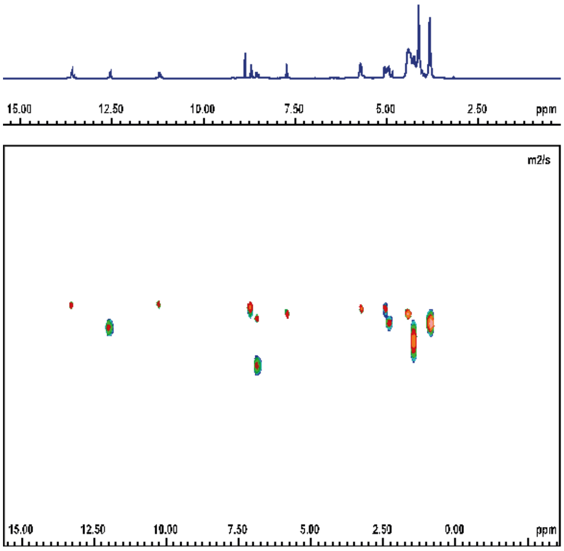

UPy.UPy 48 mM 530 nm

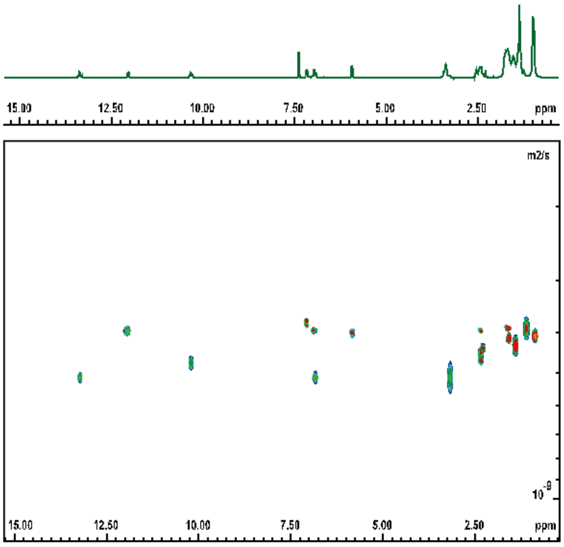

UPy.UPy 52 mM 405 nm

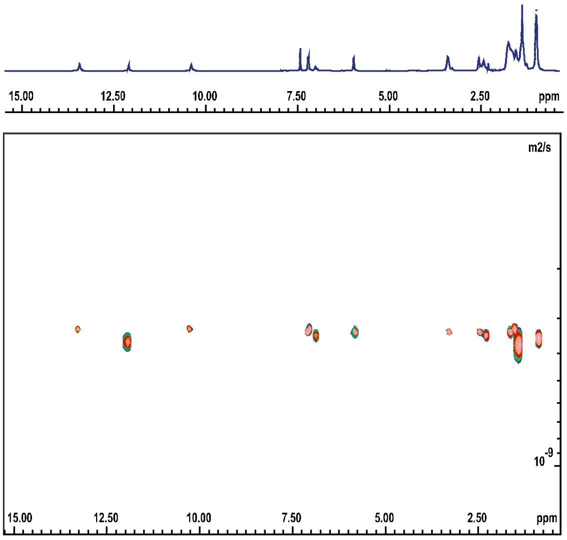

UPy.UPy 52 mM 530 nm

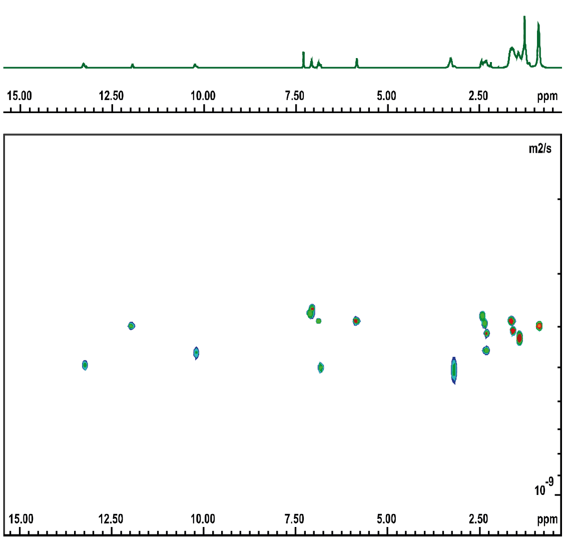

UPy.UPy 56 mM 405 nm

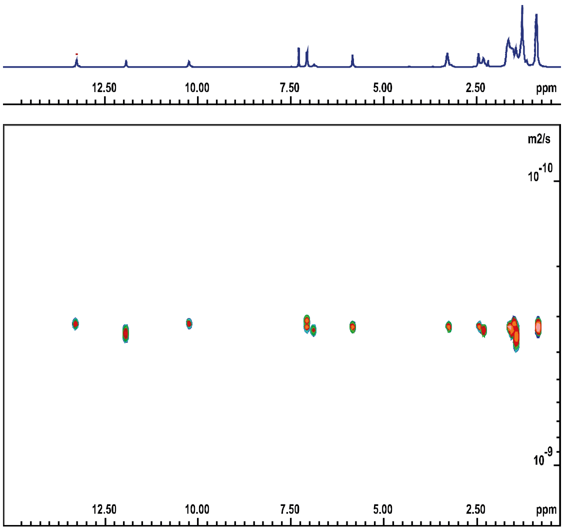

UPy.UPy 56 mM 530 nm

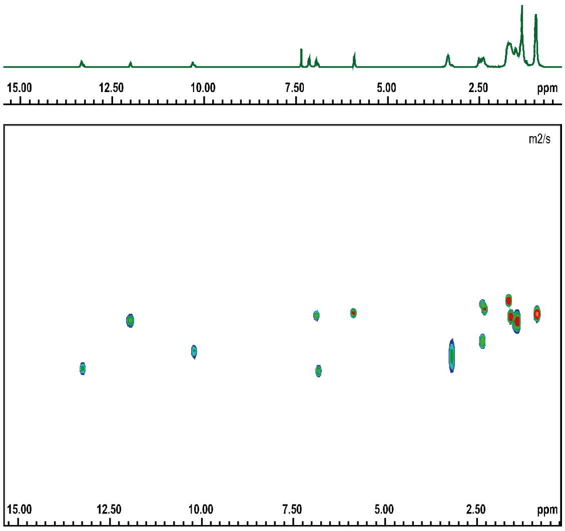

UPy.DAN 4 mM 405 nm

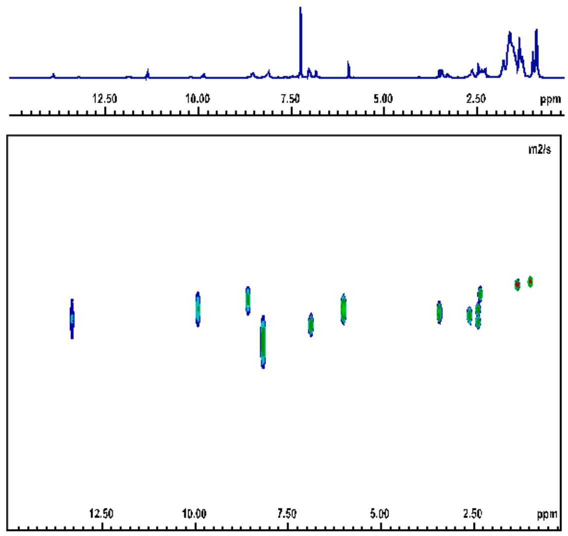

UPy.DAN 4 mM 530 nm

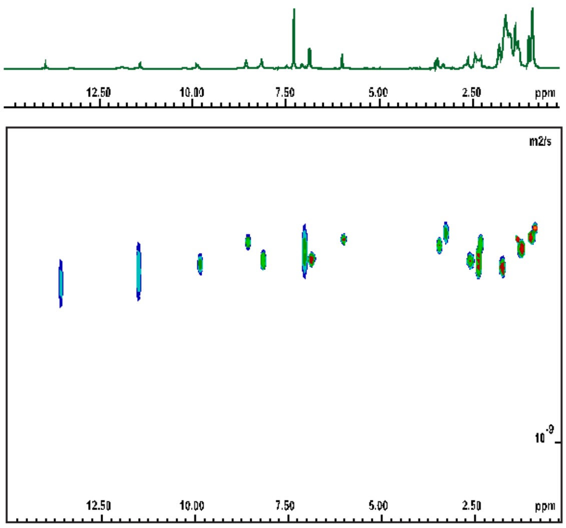

UPy.DAN 8 mM 405 nm

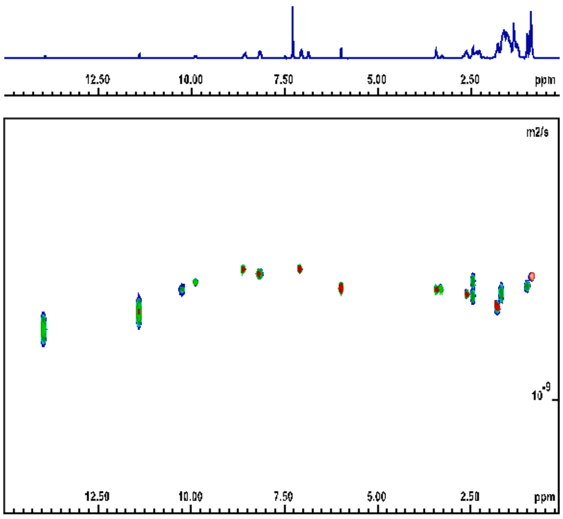

UPy.DAN 8 mM 530 nm

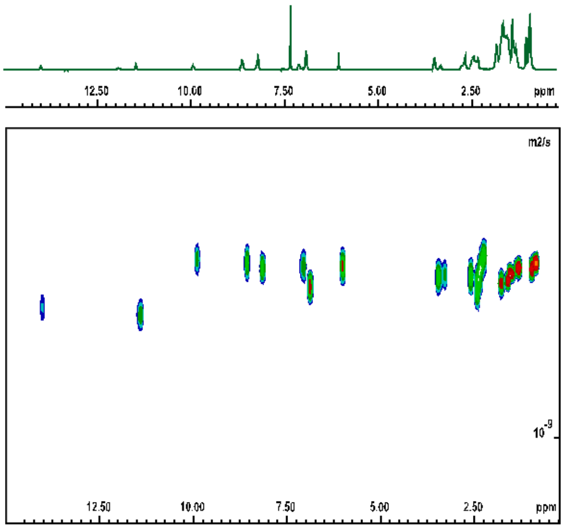

UPy.DAN 12 mM 405 nm

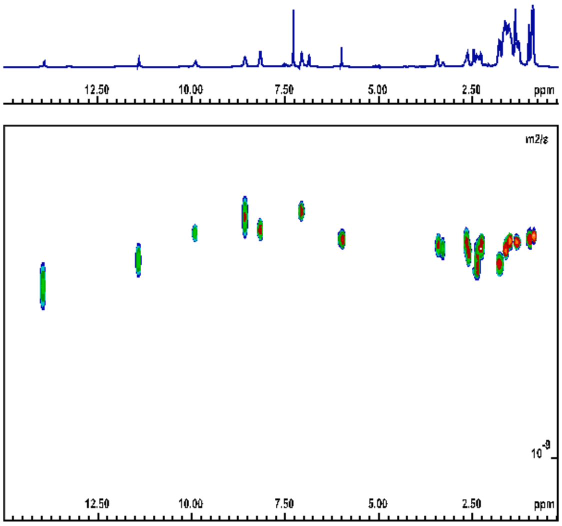

UPy.DAN 12 mM 530 nm

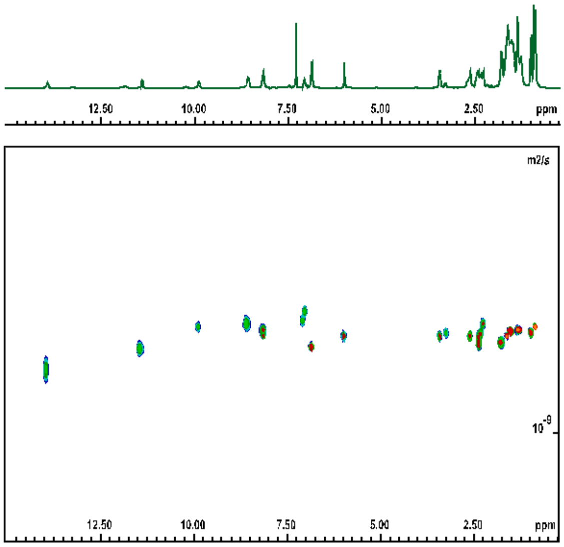

UPy.DAN 16 mM 405 nm

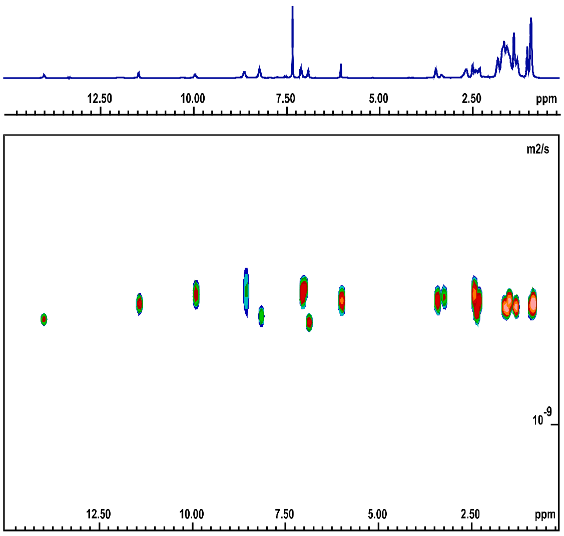

UPy.DAN 16 mM 530 nm

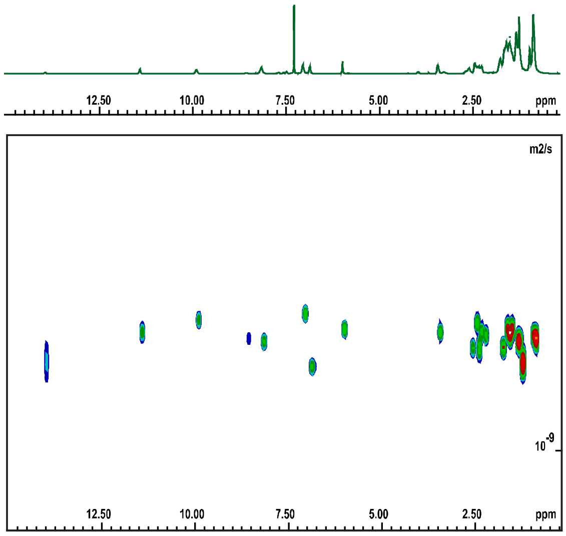

UPy.DAN 20 mM 405 nm

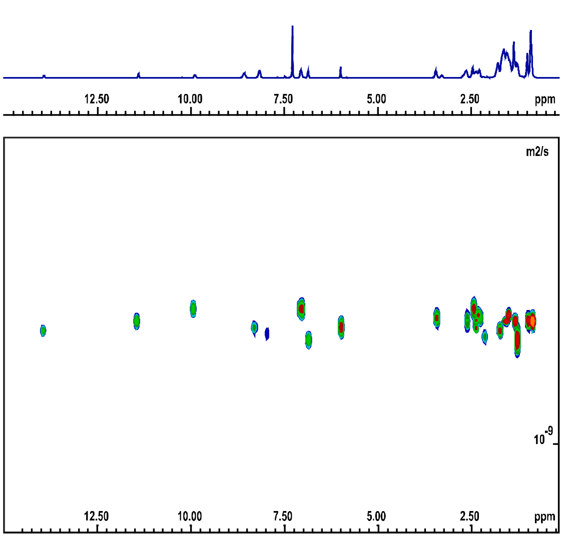

UPy.DAN 20 mM 530 nm

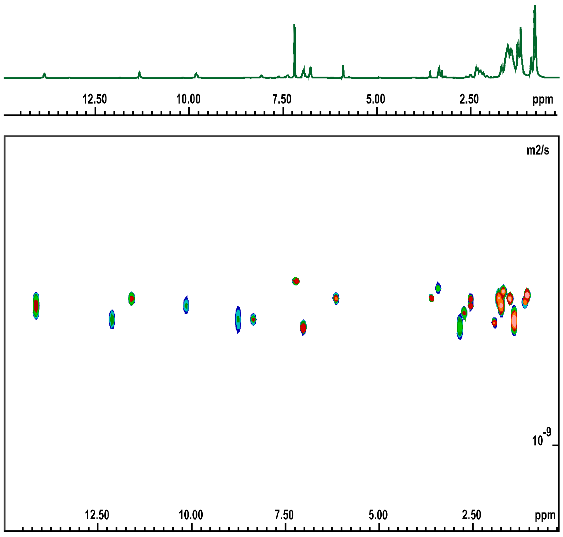

UPy.DAN 24 mM 405 nm

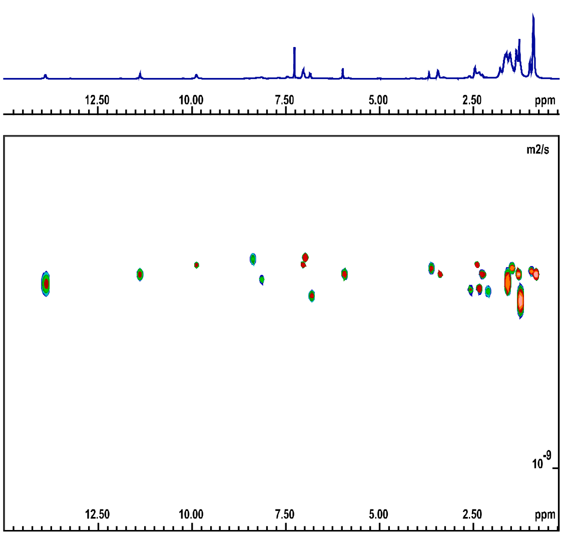

UPy.DAN 24 mM 530 nm

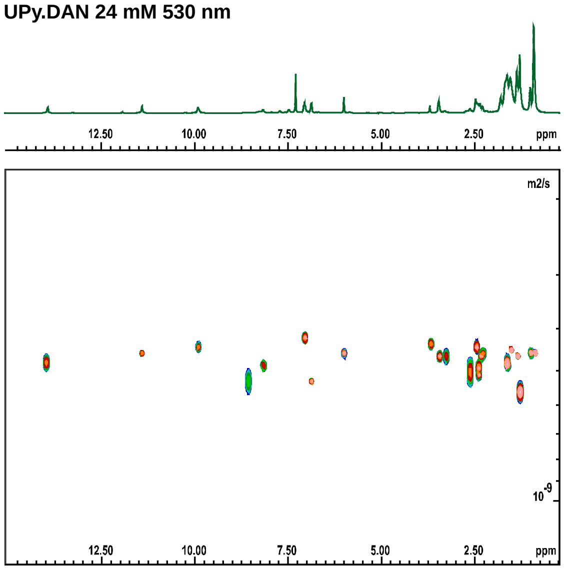

UPy.DAN 28 mM 405 nm

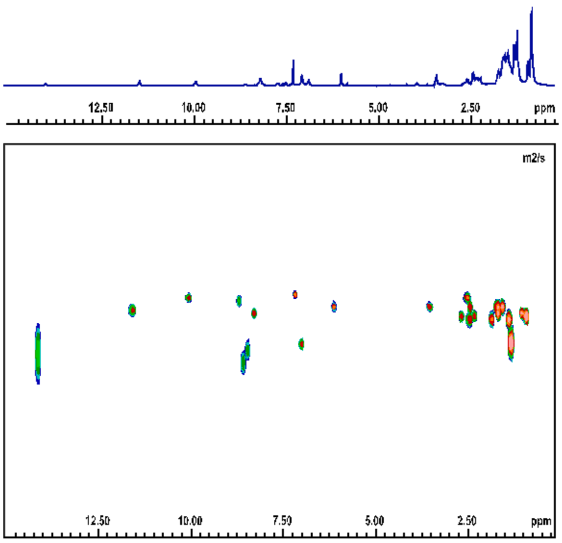

UPy.DAN 28 mM 530 nm

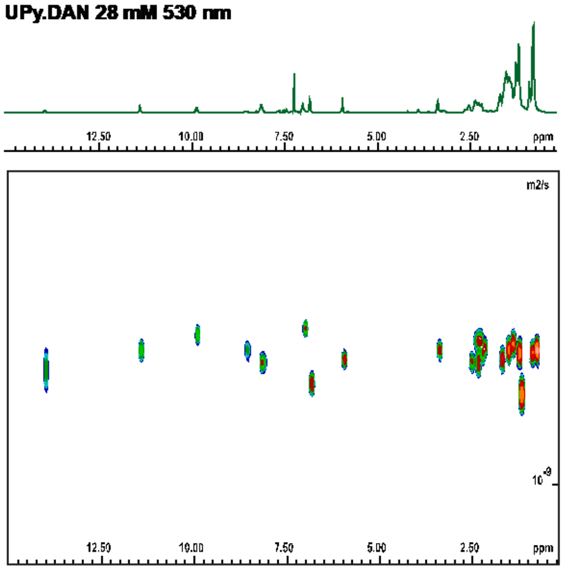

UPy.DAN 32 mM 405 nm

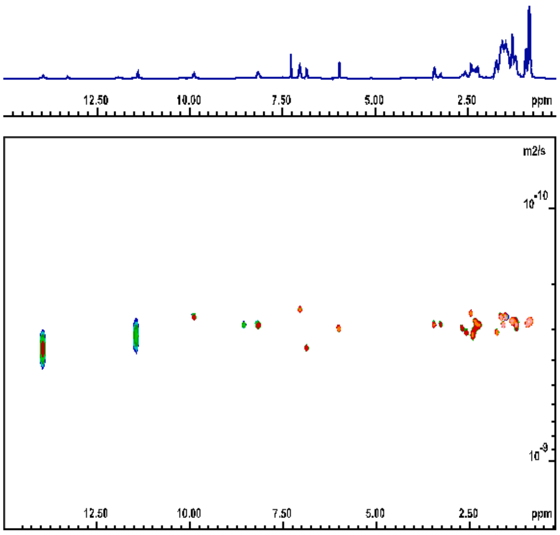

UPy.DAN 32 mM 530 nm

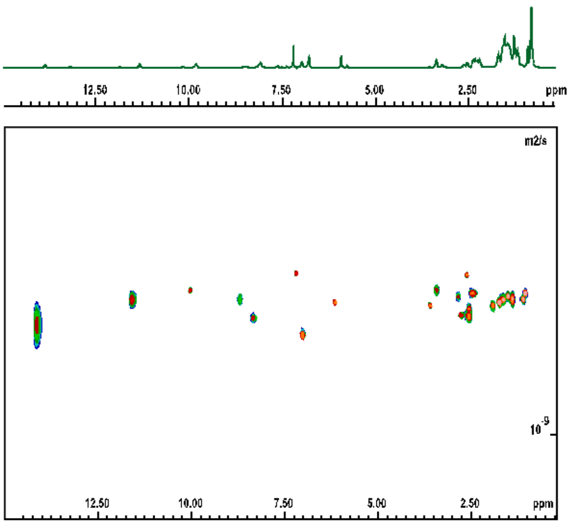

UPy.DAN 36 mM 405 nm

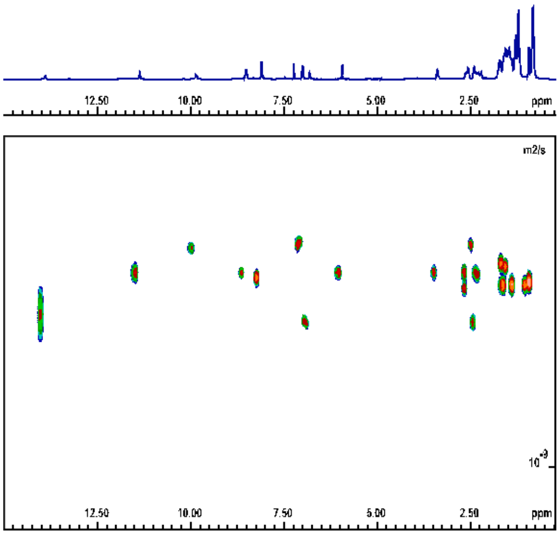

UPy.DAN 36 mM 530 nm

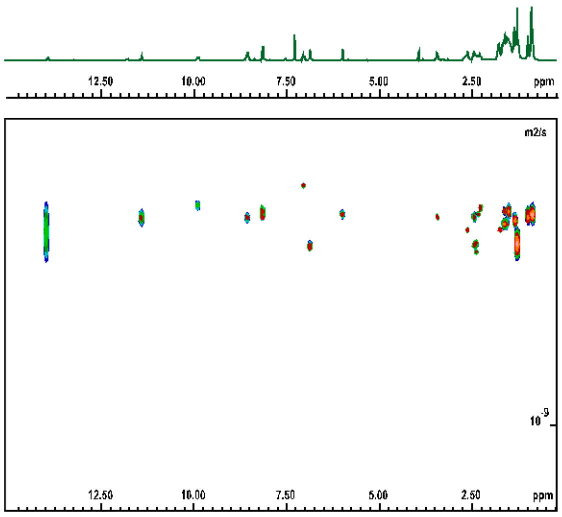

UPy.DAN 40 mM 405 nm

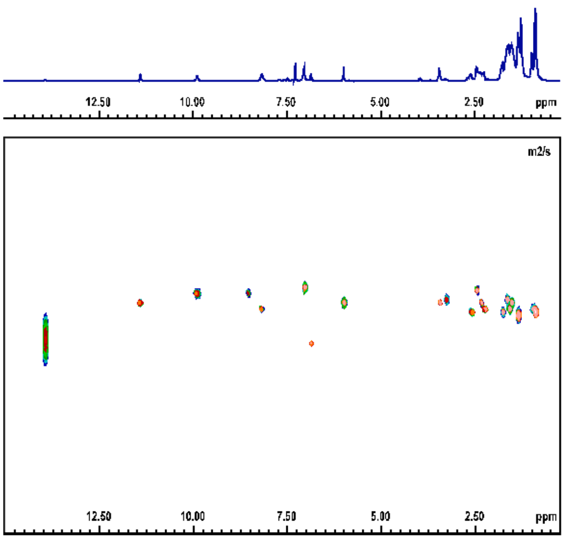

UPy.DAN 40 mM 530 nm

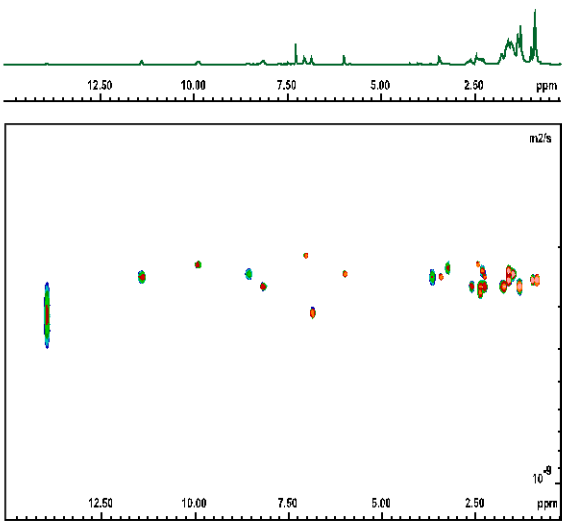

UPy.DAN 44 mM 405 nm

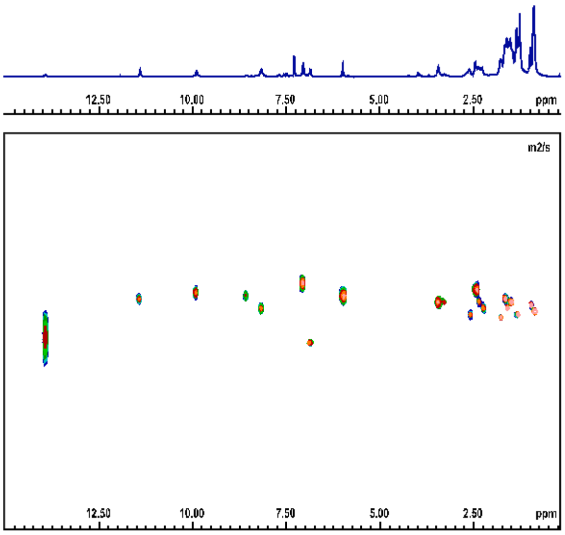

UPy.DAN 44 mM 530 nm

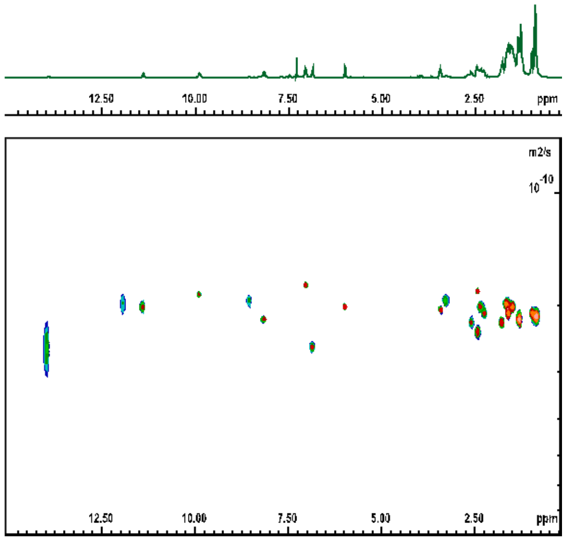

UPy.DAN 48 mM 405 nm

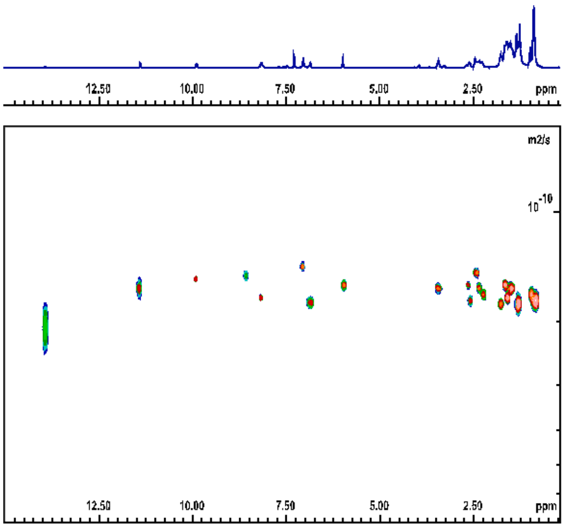

UPy.DAN 48 mM 530 nm

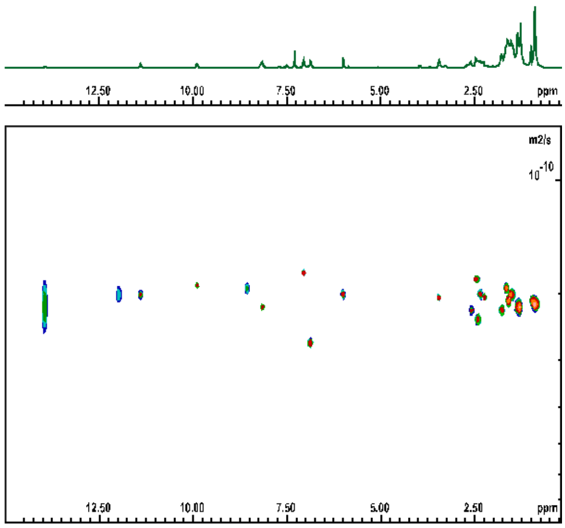

UPy.DAN 52 mM 405 nm

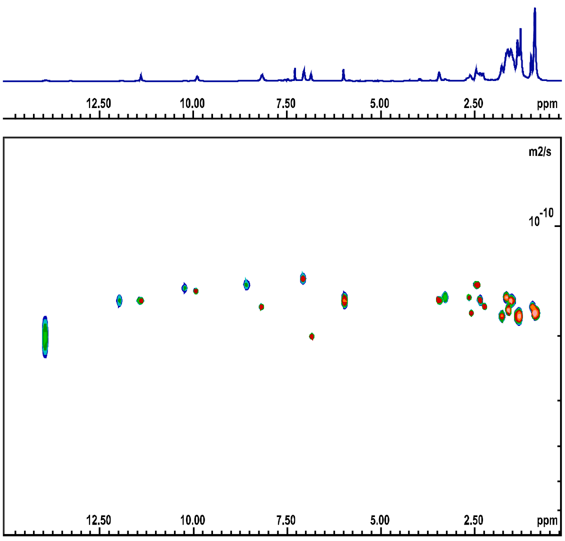

UPy.DAN 52 mM 530 nm

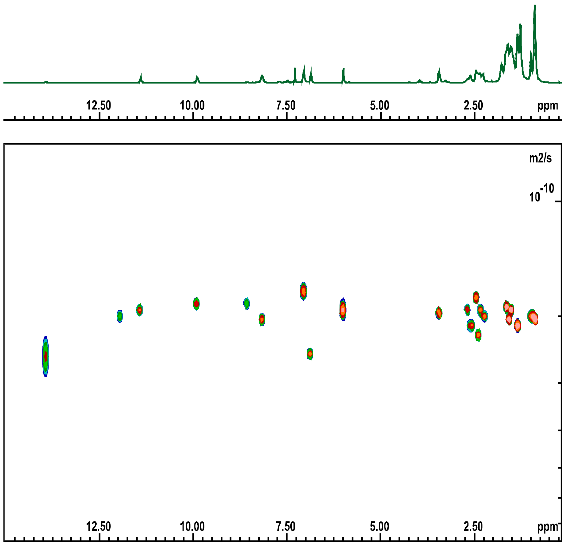

UPy.DAN 56 mM 405 nm

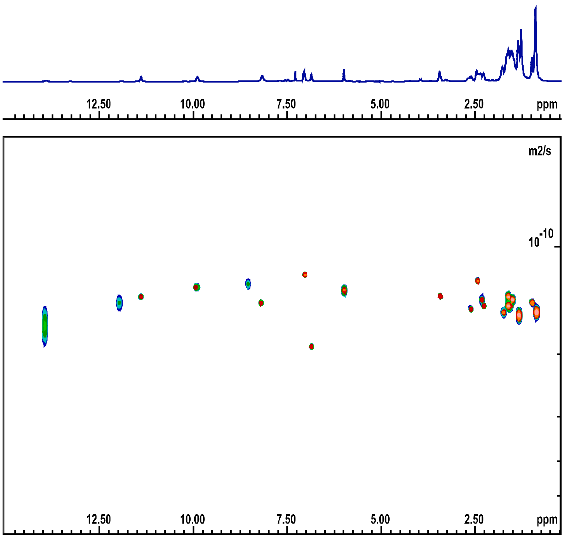

UPy.DAN 56 mM 530 nm

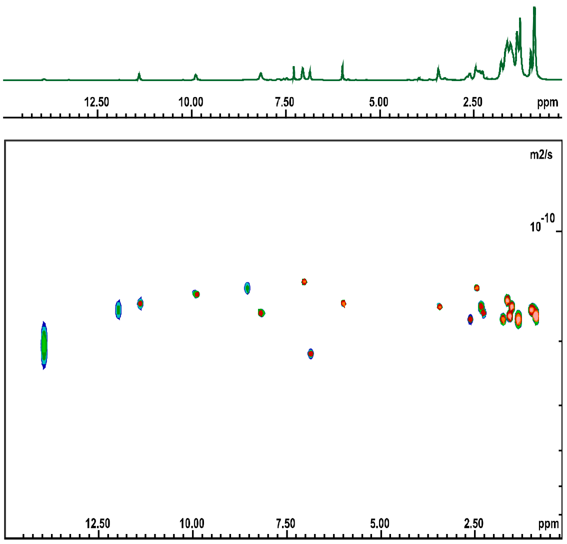

5.2 DOSY Data

From the DOSY spectra obtained reports were generated using the Bruker TopSpin Dynamics Centre software. Diffusion coefficient values were selected from the azobenzene proton peaks in the spectra. These peaks were selected as they showed clear evidence of switching after irradiation and were isolated from other proton environments in the spectra.

5.2.1 Pyr.NAP Foldamer I

Concentration (mM)	*NAP.Pyr 405 nm*		*NAP.Pyr 530 nm*	
	D (m^2/s)	Error (m^2/s)	D (m^2/s)	Error (m^2/s)
4	6.00E-10	1.96E-11	6.99E-10	1.38E-11
8	6.30E-10	5.65E-12	6.61E-10	6.06E-12
12	5.86E-10	3.07E-12	6.99E-10	1.32E-11
16	6.04E-10	3.81E-12	7.01E-10	2.70E-12
20	5.30E-10	2.36E-12	7.13E-10	4.16E-12
24	5.99E-10	1.40E-12	7.15E-10	3.11E-12
28	5.21E-10	2.59E-12	6.54E-10	6.39E-12
32	4.94E-10	1.80E-12	6.35E-10	1.92E-12
36	4.97E-10	2.27E-12	5.55E-10	2.07E-12
40	5.31E-10	4.31E-12	5.81E-10	1.65E-12
44	4.39E-10	2.10E-12	5.01E-10	1.44E-12
48	4.32E-10	7.07E-12	4.83E-10	2.90E-12
52	3.72E-10	1.29E-12	4.66E-10	2.37E-12

Table 1 Diffusion coefficients of foldamer **I** over concentration range 4 mM - 52 mM.

Concentration (mM)	*NAP.Pyr 405 nm*		*NAP.Pyr 530 nm*	
	DP	Mw (g/mol^{-1})	DP	Mw (g/mol^{-1})
4	1.58	1228.31	1.00	776.84
8	1.37	1061.06	1.18	918.67
12	1.70	1318.47	1.00	776.84
16	1.55	1204.07	0.99	770.21
20	2.29	1782.11	0.94	731.97
24	1.59	1234.48	0.93	725.85
28	2.42	1876.07	1.22	948.48
32	2.83	2200.81	1.33	1036.20
36	2.78	2161.19	2.00	1551.97
40	2.28	1772.06	1.74	1352.80
44	4.04	3135.95	2.72	2109.84
48	4.24	3290.88	3.03	2354.62
52	6.63	5153.88	3.38	2621.84

Table 2. Degrees of polymerisation and molecular weight of each isomer of foldamer **I** over concentration range 52 – 4 mM

5.2.2 UPy.UPy Foldamer II

Concentration (mM)	*UPy.UPy 405 nm*		*UPy.UPy 530 nm*	
	D (m^2/s)	Error (m^2/s)	D (m^2/s)	Error (m^2/s)
4	4.77E-10	7.34E-12	6.37E-10	1.40E-11
8	4.72E-10	6.31E-12	6.15E-10	8.71E-12
12	4.50E-10	1.50E-11	6.00E-10	9.79E-12
16	4.33E-10	5.06E-12	6.04E-10	1.19E-11
20	4.25E-10	3.49E-12	6.17E-10	1.18E-11
24	4.48E-10	6.62E-12	6.52E-10	4.19E-12
28	4.22E-10	1.52E-12	5.94E-10	8.27E-12
32	4.30E-10	6.98E-12	6.23E-10	4.96E-12
36	3.83E-10	4.70E-12	5.89E-10	5.40E-12
40	3.80E-10	3.46E-12	5.48E-10	1.35E-11
44	3.46E-10	4.81E-12	5.10E-10	1.06E-11
48	3.34E-10	1.37E-12	5.31E-10	1.15E-11
52	3.27E-10	2.21E-12	5.21E-10	9.39E-12

Table 3 Diffusion coefficients of foldamer **II** over concentration range 4 mM - 52 mM

Concentration (mM)	*UPy.UPy 405 nm*		*UPy.UPy 530 nm*	
	DP	Mw (g/mol^{-1})	DP	Mw (g/mol^{-1})
4	2.38	2312.88	1.00	971.16
8	2.46	2387.17	1.11	1079.15
12	2.84	2754.68	1.20	1162.13
16	3.18	3092.04	1.17	1139.20
20	3.37	3269.96	1.10	1068.69
24	2.87	2791.74	0.93	905.66
28	3.44	3340.19	1.23	1197.70
32	3.25	3157.21	1.07	1038.11
36	4.60	4468.00	1.26	1228.47
40	4.71	4574.65	1.57	1525.34
44	6.24	6060.11	1.95	1892.34
48	6.94	6737.05	1.73	1676.58
52	7.39	7179.03	1.83	1774.99

Table 4 . Degrees of polymerisation and molecular weight of each isomer of foldamer **II** over concentration range 52 – 4 mM

5.2.3 UPy.DAN Foldamer III

Concentration (mM)	*UPy.DAN 405 nm*		*UPy.DAN 530 nm*	
	**D (m^2/s)**	**Error (m^2/s)**	**D (m^2/s)**	**Error (m^2/s)**
4	5.33E-10	1.02E-11	6.60E-10	4.26E-12
8	4.98E-10	2.65E-12	5.46E-10	1.66E-11
12	5.02E-10	6.64E-12	5.84E-10	3.92E-12
16	3.92E-10	8.62E-12	5.45E-10	1.62E-11
20	3.66E-10	2.96E-12	5.63E-10	4.41E-12
24	2.98E-10	2.51E-12	5.32E-10	2.95E-12
28	2.62E-10	2.16E-12	4.39E-10	1.04E-11
32	2.57E-10	7.50E-13	3.26E-10	2.08E-12
36	2.35E-10	2.98E-12	3.13E-10	3.96E-12
40	2.03E-10	2.06E-12	3.16E-10	2.81E-12
44	1.87E-10	4.03E-12	2.71E-10	3.31E-12
48	1.42E-10	1.33E-12	2.67E-10	2.50E-12
52	1.40E-10	1.47E-12	2.52E-10	2.91E-12

Table 5 Diffusion coefficients of foldamer **III** over concentration range 4 mM - 52 mM

Concentration (mM)	*UPy.DAN 405 nm*		*UPy.DAN 530 nm*	
	**DP**	**Mw (g/mol^{-1})**	**DP**	**Mw (g/mol^{-1})**
4	1.90	1961.77	1.00	1033.23
8	2.33	2405.14	1.77	1824.95
12	2.27	2348.11	1.44	1491.39
16	4.77	4931.40	1.78	1835.01
20	5.86	6058.79	1.61	1664.58
24	10.86	11224.84	1.91	1972.85
28	15.99	16516.76	3.40	3511.04
32	16.94	17499.65	8.30	8573.85
36	22.15	22888.92	9.38	9687.15
40	34.37	35509.18	9.11	9413.86
44	43.96	45425.95	14.45	14925.23
48	100.41	103744.19	15.10	15606.12
52	104.77	108254.19	17.97	18562.10

Table 6 . Degrees of polymerisation and molecular weight of each isomer of foldamer **III** over concentration range 52 – 4 mM

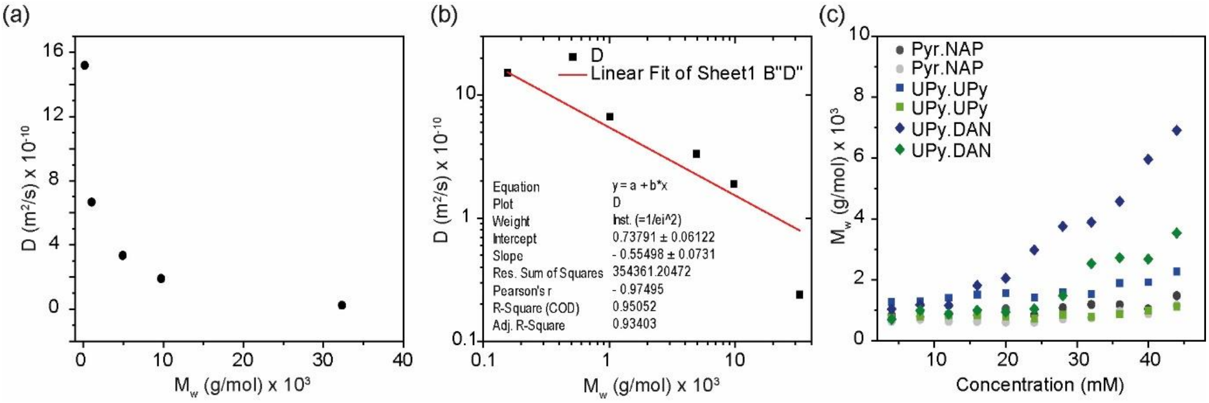

Figure S2. A DOSY calibration curve was created by measuring the ^{1}H DOSY spectra for a series of standards (2,2'-Dipyridyl (M_w = 156) and PMMA GPC standards (M_p = 1010, 4920, 9720, 32340)) and used to determined M_w values for hydrogen-bond assembled supramolecular polymers (a) diffusion values for GPC standards (b) calibration plot (c) recalculated M_w values based on calibration plot, the values are lower than those determined using the equation $M_w = (D_{monomer}/D)^3$, which we consider to be unrealistic given the association constants for the UPy·Upy and DAN·UPy dimers and can be attributed to a difference in viscosity between the standards and the hydrogen-bond assembled polymers.

6. Viscometry

Viscosity measurements were carried out using a micro-Ostwald capillary viscometer purchased from VWR international. Solutions were prepared in anhydrous deuterated chloroform, irradiated, and then allowed to equilibrate to 298 K in a thermostatic water bath for 5 minutes. For each concentration, 4 readings were taken and an average time was used to calculate the specific viscosity.

6.1 Pyr.NAP Foldamer I

Conc. (mM)	*λ (nm)*	*Isomer*	*T1 (s)*	*T2 (s)*	*T3 (s)*	*T4 (s)*	*Avg T(s)*	$^{T1}\eta_{sp}$ *(N/m²)*	$^{T2}\eta_{sp}$ *(N/m²)*	$^{T3}\eta_{sp}$ *(N/m²)*	$^{T4}\eta_{sp}$ *(N/m²)*	$^{Avg}\eta_{sp}$ *(N/m²)*
56	405	*E*	0.70	0.72	0.72	0.71	0.71	0.09	0.12	0.12	0.10	0.10
	530	*Z*	0.71	0.71	0.69	0.71	0.71	0.10	0.10	0.07	0.10	0.09
52	405	*E*	0.70	0.71	0.70	0.72	0.71	0.09	0.10	0.09	0.12	0.10
	530	*Z*	0.70	0.69	0.71	0.69	0.70	0.09	0.07	0.10	0.07	0.08
48	405	*E*	0.71	0.68	0.70	0.69	0.70	0.10	0.05	0.09	0.07	0.08
	530	*Z*	0.69	0.68	0.69	0.70	0.69	0.07	0.05	0.07	0.09	0.07
44	405	*E*	0.70	0.69	0.68	0.70	0.69	0.09	0.07	0.05	0.09	0.07
	530	*Z*	0.68	0.70	0.68	0.69	0.69	0.05	0.09	0.05	0.07	0.07
40	405	*E*	0.69	0.67	0.68	0.70	0.69	0.07	0.04	0.05	0.09	0.06
	530	*Z*	0.67	0.69	0.68	0.69	0.68	0.04	0.07	0.05	0.07	0.06
36	405	*E*	0.70	0.67	0.68	0.68	0.68	0.09	0.04	0.05	0.05	0.06
	530	*Z*	0.67	0.67	0.69	0.68	0.68	0.04	0.04	0.07	0.05	0.05
32	405	*E*	0.68	0.69	0.67	0.67	0.68	0.05	0.07	0.04	0.04	0.05
	530	*Z*	0.67	0.66	0.69	0.68	0.68	0.04	0.02	0.07	0.05	0.05
28	405	*E*	0.66	0.68	0.67	0.67	0.67	0.02	0.05	0.04	0.04	0.04
	530	*Z*	0.68	0.66	0.67	0.66	0.67	0.05	0.02	0.04	0.02	0.03
24	405	*E*	0.65	0.67	0.68	0.68	0.67	0.01	0.04	0.05	0.05	0.04
	530	*Z*	0.66	0.67	0.65	0.66	0.66	0.02	0.04	0.01	0.02	0.02
20	405	*E*	0.66	0.65	0.68	0.66	0.66	0.02	0.01	0.05	0.02	0.03
	530	*Z*	0.65	0.67	0.66	0.65	0.66	0.01	0.04	0.02	0.01	0.02
16	405	*E*	0.66	0.67	0.65	0.66	0.66	0.02	0.04	0.01	0.02	0.02
	530	*Z*	0.66	0.65	0.67	0.64	0.66	0.02	0.01	0.04	-0.01	0.02
12	405	*E*	0.67	0.65	0.65	0.66	0.66	0.04	0.01	0.01	0.02	0.02
	530	*Z*	0.65	0.67	0.65	0.64	0.65	0.01	0.04	0.01	-0.01	0.01
8	405	*E*	0.66	0.65	0.64	0.66	0.65	0.02	0.01	-0.01	0.02	0.01
	530	*Z*	0.64	0.65	0.66	0.65	0.65	-0.01	0.01	0.02	0.01	0.01
4	405	*E*	0.65	0.66	0.63	0.65	0.65	0.01	0.02	-0.02	0.01	0.00
	530	*Z*	0.64	0.65	0.65	0.65	0.65	-0.01	0.01	0.01	0.01	0.00
$CDCl_3$	N/A	N/A	0.64	0.64	0.65	0.65	0.65	N/A	N/A	N/A	N/A	N/A

Table 7. Viscosity measurements for foldamer **I** over concentration range 56 – 4 mM.

6.2 UPy.UPy Foldamer II

Conc. (mM)	*λ (nm)*	*Isomer*	*T1 (s)*	*T2 (s)*	*T3 (s)*	*T4 (s)*	*Avg T (s)*	*$^{T1}\eta_{sp}$ (N/m²)*	*$^{T2}\eta_{sp}$ (N/m²)*	*$^{T3}\eta_{sp}$ (N/m²)*	*$^{T4}\eta_{sp}$ (N/m²)*	*$^{Avg}\eta_{sp}$ (N/m²)*
56	405	*E*	5.32	5.31	5.35	5.34	5.33	7.18	3.17	3.15	3.14	7.20
	530	*Z*	4.42	4.41	4.43	4.44	4.43	5.80	2.32	2.29	2.34	5.81
52	405	*E*	4.30	4.33	4.30	4.31	4.31	5.62	2.35	2.38	2.34	5.63
	530	*Z*	3.39	3.37	3.38	3.39	3.38	4.22	1.54	1.52	1.58	4.20
48	405	*E*	3.47	3.48	3.48	3.49	3.48	4.34	1.72	1.74	1.62	4.35
	530	*Z*	2.62	2.65	2.62	2.61	2.63	3.03	0.86	0.89	0.92	3.04
44	405	*E*	2.68	2.71	2.70	2.69	2.70	3.12	1.34	1.32	1.31	3.15
	530	*Z*	2.15	2.16	2.14	2.17	2.16	2.31	0.57	0.63	0.62	2.32
40	405	*E*	2.19	2.18	2.20	2.17	2.19	2.37	0.91	0.89	0.91	2.36
	530	*Z*	1.67	1.65	1.64	1.68	1.66	1.57	0.34	0.35	0.32	1.55
36	405	*E*	1.74	1.77	1.78	1.70	1.75	1.68	0.55	0.62	0.60	1.69
	530	*Z*	1.24	1.21	1.23	1.25	1.23	0.91	0.22	0.17	0.18	0.90
32	405	*E*	1.53	1.52	1.51	1.50	1.52	1.35	0.43	0.35	0.38	1.33
	530	*Z*	1.04	1.02	1.06	1.05	1.04	0.60	0.14	0.14	0.12	0.60
28	405	*E*	1.22	1.24	1.23	1.24	1.23	0.88	0.18	0.17	0.22	0.90
	530	*Z*	0.89	0.87	0.88	0.86	0.88	0.37	0.12	0.11	0.08	0.35
24	405	*E*	1.02	1.01	1.05	1.04	1.03	0.57	0.08	0.11	0.11	0.58
	530	*Z*	0.78	0.79	0.76	0.77	0.78	0.20	0.06	0.05	0.08	0.19
20	405	*E*	0.89	0.93	0.88	0.90	0.90	0.37	0.02	0.03	0.05	0.38
	530	*Z*	0.75	0.74	0.74	0.73	0.74	0.15	0.03	0.03	0.05	0.14
16	405	*E*	0.78	0.77	0.76	0.79	0.78	0.20	0.18	0.00	-0.02	0.19
	530	*Z*	0.73	0.73	0.72	0.70	0.72	0.12	0.12	0.02	0.02	0.11
12	405	*E*	0.71	0.70	0.72	0.72	0.71	0.09	0.08	0.11	0.11	0.10
	530	*Z*	0.69	0.69	0.68	0.70	0.69	0.06	0.06	0.05	0.08	0.06
8	405	*E*	0.69	0.66	0.67	0.68	0.68	0.06	0.02	0.03	0.05	0.04
	530	*Z*	0.67	0.67	0.67	0.68	0.67	0.03	0.03	0.03	0.05	0.03
4	405	*E*	0.66	0.67	0.65	0.64	0.66	0.02	0.03	0.00	-0.02	0.01
	530	*Z*	0.65	0.65	0.66	0.66	0.66	0.00	0.00	0.02	0.02	0.01
$CDCl_3$	N/A	N/A	0.65	0.63	0.66	0.66	0.65	N/A	N/A	N/A	N/A	N/A

Table 8. Viscosity measurements for foldamer **II** over concentration range 56 – 4 mM.

Cycle 56 mM	*λ (nm)*	*Isomer*	*T1 (s)*	*T2 (s)*	*T3 (s)*	*T4 (s)*	*Avg T (s)*	*$^{T1}\eta_{sp}$ (N/m^2)*	*$^{T2}\eta_{sp}$ (N/m^2)*	*$^{T3}\eta_{sp}$ (N/m^2)*	*$^{T4}\eta_{sp}$ (N/m^2)*	*$^{Avg}\eta_{sp}$ (N/m^2)*
0.5	405	*E*	5.32	5.31	5.35	5.34	5.33	7.18	7.17	7.23	7.22	7.20
1.0	530	*Z*	4.42	4.41	4.43	4.44	4.43	5.80	5.78	5.82	5.83	5.81
1.5	405	*E*	5.29	5.34	5.29	5.36	5.32	7.14	7.22	7.14	7.25	7.18
2.0	530	*Z*	4.43	4.45	4.41	4.37	4.42	5.82	5.85	5.78	5.72	5.79
2.5	405	*E*	5.31	5.27	5.29	5.36	5.31	7.17	7.11	7.14	7.25	7.17
3.0	530	*Z*	4.45	4.40	4.39	4.41	4.41	5.85	5.77	5.75	5.78	5.79
3.5	405	*E*	5.34	5.27	5.32	5.37	5.33	7.22	7.11	7.18	7.26	7.19
4.0	530	*Z*	4.39	4.48	4.40	4.41	4.42	5.75	5.89	5.77	5.78	5.80
4.5	405	*E*	5.36	5.29	5.34	5.34	5.33	7.25	7.14	7.22	7.22	7.20
5.0	530	*Z*	4.47	4.40	4.48	4.40	4.44	5.88	5.77	5.89	5.77	5.83
5.5	405	*E*	5.36	5.39	5.38	5.37	5.38	7.25	7.29	7.28	7.26	7.27
6.0	530	*Z*	4.46	4.42	4.45	4.50	4.46	5.86	5.80	5.85	5.92	5.86
6.5	405	*E*	5.42	5.39	5.37	5.35	5.38	7.34	7.29	7.26	7.23	7.28
7.0	530	*Z*	4.44	4.42	4.38	4.43	4.42	5.83	5.80	5.74	5.82	5.80
7.5	405	*E*	5.38	5.40	5.44	5.37	5.40	7.28	7.31	7.37	7.26	7.30
8.0	530	*Z*	4.50	4.51	4.49	4.43	4.48	5.92	5.94	5.91	5.82	5.90
8.5	405	*E*	5.36	5.48	5.39	5.44	5.42	7.25	7.43	7.29	7.37	7.33
9.0	530	*Z*	4.51	4.47	4.53	4.52	4.51	5.94	5.88	5.97	5.95	5.93
9.5	405	*E*	5.40	5.37	5.43	5.39	5.40	7.31	7.26	7.35	7.29	7.30
10.0	530	*Z*	4.47	4.47	4.50	4.53	4.49	5.88	5.88	5.92	5.97	5.91

Table 9. Viscosity measurements for foldamer **II** (56 mM) after 10 cycles of irradiation at 405 nm and 530 nm.

Cycle 32 mM	*λ (nm)*	*Isomer*	*T1 (s)*	*T2 (s)*	*T3 (s)*	*T4 (s)*	*Avg T (s)*	*$^{T1}\eta_{sp}$ (N/m^2)*	*$^{T2}\eta_{sp}$ (N/m^2)*	*$^{T3}\eta_{sp}$ (N/m^2)*	*$^{T4}\eta_{sp}$ (N/m^2)*	*$^{Avg}\eta_{sp}$ (N/m^2)*
0.5	405	*E*	1.53	1.52	1.51	1.50	1.52	1.35	1.34	1.35	1.35	1.33
1.0	530	*Z*	1.04	1.02	1.06	1.05	1.04	0.60	0.65	0.60	0.68	0.60
1.5	405	*E*	1.53	1.54	1.50	1.59	1.54	1.35	1.42	1.32	1.31	1.37
2.0	530	*Z*	1.06	1.07	1.09	1.06	1.07	0.63	0.68	0.66	0.66	0.65
2.5	405	*E*	1.50	1.51	1.49	1.52	1.51	1.31	1.32	1.29	1.34	1.32
3.0	530	*Z*	1.09	1.08	1.05	1.04	1.07	0.68	0.66	0.62	0.60	0.64
3.5	405	*E*	1.56	1.52	1.53	1.53	1.54	1.40	1.34	1.35	1.35	1.36
4.0	530	*Z*	1.10	1.07	1.04	1.09	1.08	0.69	0.65	0.60	0.68	0.65
4.5	405	*E*	1.58	1.57	1.51	1.50	1.54	1.43	1.42	1.32	1.31	1.37
5.0	530	*Z*	1.07	1.09	1.08	1.08	1.08	0.65	0.68	0.66	0.66	0.66
5.5	405	*E*	1.54	1.56	1.55	1.56	1.55	1.37	1.40	1.38	1.40	1.39
6.0	530	*Z*	1.12	1.09	1.10	1.11	1.11	0.72	0.68	0.69	0.71	0.70
6.5	405	*E*	1.53	1.50	1.52	1.59	1.54	1.35	1.31	1.34	1.45	1.36
7.0	530	*Z*	1.10	1.07	1.12	1.09	1.10	0.69	0.65	0.72	0.68	0.68
7.5	405	*E*	1.56	1.55	1.53	1.56	1.55	1.40	1.38	1.35	1.40	1.38
8.0	530	*Z*	1.12	1.11	1.09	1.08	1.10	0.72	0.71	0.68	0.66	0.69
8.5	405	*E*	1.54	1.55	1.55	1.57	1.55	1.37	1.38	1.38	1.42	1.39
9.0	530	*Z*	1.11	1.14	1.10	1.12	1.12	0.71	0.75	0.69	0.72	0.72
9.5	405	*E*	1.57	1.58	1.57	1.60	1.58	1.42	1.43	1.42	1.46	1.43
10.0	530	*Z*	1.13	1.11	1.15	1.09	1.12	0.74	0.71	0.77	0.68	0.72

Table 10. Viscosity measurements for foldamer **II** (32 mM) after 10 cycles of irradiation at 405 nm and 530 nm

Cycle 4 mM	*λ (nm)*	*Isomer*	*T1 (s)*	*T2 (s)*	*T3 (s)*	*T4 (s)*	*Avg T (s)*	*$^{T1}\eta_{sp}$ (N/m^2)*	*$^{T2}\eta_{sp}$ (N/m^2)*	*$^{T3}\eta_{sp}$ (N/m^2)*	*$^{T4}\eta_{sp}$ (N/m^2)*	*$^{Avg}\eta_{sp}$ (N/m^2)*
0.5	405	*E*	0.66	0.67	0.65	0.64	0.66	0.02	0.03	0.00	-0.02	0.01
1.0	530	*Z*	0.65	0.65	0.66	0.66	0.66	0.00	0.00	0.02	0.02	0.01
1.5	405	*E*	0.65	0.65	0.67	0.66	0.66	0.00	0.00	0.03	0.02	0.01
2.0	530	*Z*	0.63	0.62	0.69	0.69	0.66	-0.03	-0.05	0.06	0.06	0.01
2.5	405	*E*	0.65	0.66	0.67	0.69	0.67	0.00	0.02	0.03	0.06	0.03
3.0	530	*Z*	0.68	0.61	0.67	0.68	0.66	0.05	-0.06	0.03	0.05	0.02
3.5	405	*E*	0.67	0.65	0.70	0.65	0.67	0.03	0.00	0.08	0.00	0.03
4.0	530	*Z*	0.66	0.66	0.68	0.67	0.67	0.02	0.02	0.05	0.03	0.03
4.5	405	*E*	0.64	0.67	0.65	0.69	0.66	-0.02	0.03	0.00	0.06	0.02
5.0	530	*Z*	0.69	0.65	0.66	0.66	0.67	0.06	0.00	0.02	0.02	0.02
5.5	405	*E*	0.65	0.70	0.65	0.63	0.66	0.00	0.08	0.00	-0.03	0.01
6.0	530	*Z*	0.68	0.65	0.63	0.67	0.66	0.05	0.00	-0.03	0.03	0.01
6.5	405	*E*	0.69	0.67	0.61	0.68	0.66	0.06	0.03	-0.06	0.05	0.02
7.0	530	*Z*	0.65	0.65	0.68	0.66	0.66	0.00	0.00	0.05	0.02	0.02
7.5	405	*E*	0.68	0.67	0.66	0.69	0.68	0.05	0.03	0.02	0.06	0.04
8.0	530	*Z*	0.65	0.68	0.70	0.66	0.67	0.00	0.05	0.08	0.02	0.03
8.5	405	*E*	0.69	0.67	0.63	0.66	0.66	0.06	0.03	-0.03	0.02	0.02
9.0	530	*Z*	0.67	0.66	0.69	0.67	0.67	0.03	0.02	0.06	0.03	0.03
9.5	405	*E*	0.66	0.65	0.66	0.69	0.67	0.02	0.00	0.02	0.06	0.02
10.0	530	*Z*	0.68	0.66	0.70	0.65	0.67	0.05	0.02	0.08	0.00	0.03

Table 11. Viscosity measurements for foldamer **II** (4 mM) after 10 cycles of irradiation at 405 nm and 530 nm.

6.3 UPy.DAN Foldamer III

Conc. (mM)	*λ (nm)*	*Isomer*	*T1 (s)*	*T2 (s)*	*T3 (s)*	*T4 (s)*	*Avg T (s)*	$^{T1}\eta_{sp}$ *(N/m²)*	$^{T2}\eta_{sp}$ *(N/m²)*	$^{T3}\eta_{sp}$ *(N/m²)*	$^{T4}\eta_{sp}$ *(N/m²)*	$^{Avg}\eta_{sp}$ *(N/m²)*
56	405	*E*	16.61	16.59	16.60	16.57	16.59	22.73	8.47	8.44	8.44	22.70
	530	*Z*	7.91	7.92	7.89	7.87	7.90	10.30	4.16	4.11	4.10	10.28
52	405	*E*	12.75	12.70	12.75	12.73	12.73	17.21	6.51	6.53	6.56	17.19
	530	*Z*	6.27	6.26	6.23	6.28	6.26	7.96	3.00	2.99	2.97	7.94
48	405	*E*	9.84	9.86	9.89	9.92	9.88	13.06	4.97	5.04	4.96	13.11
	530	*Z*	5.00	5.03	5.01	5.04	5.02	6.14	1.87	1.86	1.87	6.17
44	405	*E*	6.59	6.63	6.61	6.61	6.61	8.41	3.39	3.40	3.34	8.44
	530	*Z*	3.62	3.61	3.58	3.57	3.60	4.17	1.14	1.19	1.16	4.14
40	405	*E*	5.28	5.26	5.27	5.29	5.28	6.54	2.26	2.29	2.24	6.54
	530	*Z*	2.77	2.80	2.79	2.78	2.79	2.96	0.64	0.57	0.56	2.98
36	405	*E*	4.16	4.18	4.23	4.17	4.19	4.94	1.36	1.33	1.29	4.98
	530	*Z*	2.03	2.01	2.00	2.01	2.01	1.90	0.31	0.30	0.27	1.88
32	405	*E*	3.01	3.07	3.08	3.04	3.05	3.30	0.76	0.71	0.73	3.36
	530	*Z*	1.52	1.50	1.53	1.51	1.52	1.17	0.20	0.20	0.19	1.16
28	405	*E*	2.23	2.28	2.30	2.27	2.27	2.19	0.36	0.33	0.31	2.24
	530	*Z*	1.11	1.15	1.10	1.09	1.11	0.59	0.10	0.13	0.13	0.59
24	405	*E*	1.59	1.65	1.63	1.60	1.62	1.27	0.17	0.13	0.19	1.31
	530	*Z*	0.90	0.92	0.91	0.89	0.91	0.29	0.09	0.07	-0.03	0.29
20	405	*E*	1.19	1.23	1.20	1.21	1.21	0.70	0.00	0.11	0.03	0.73
	530	*Z*	0.85	0.84	0.84	0.83	0.84	0.21	0.01	0.03	0.03	0.20
16	405	*E*	0.94	0.95	0.93	0.92	0.94	0.34	0.36	0.00	0.01	0.34
	530	*Z*	0.78	0.77	0.79	0.79	0.78	0.11	0.10	0.00	-0.01	0.12
12	405	*E*	0.81	0.82	0.79	0.83	0.81	0.16	0.17	0.13	0.19	0.16
	530	*Z*	0.77	0.76	0.75	0.68	0.74	0.10	0.09	0.07	-0.03	0.06
8	405	*E*	0.70	0.70	0.78	0.72	0.73	0.00	0.00	0.11	0.03	0.04
	530	*Z*	0.72	0.71	0.72	0.72	0.72	0.03	0.01	0.03	0.03	0.03
4	405	*E*	0.73	0.69	0.70	0.71	0.71	0.04	-0.01	0.00	0.01	0.01
	530	*Z*	0.71	0.70	0.70	0.69	0.70	0.01	0.00	0.00	-0.01	0.00
$CDCl_3$	N/A	N/A	0.70	0.69	0.71	0.70	0.70	N/A	N/A	N/A	N/A	N/A

Table 12. Viscosity measurements for foldamer **III** over concentration range 56 – 4 mM.

Cycle 56 mM	*λ (nm)*	*Isomer*	*T1 (s)*	*T2 (s)*	*T3 (s)*	*T4 (s)*	*Avg T (s)*	*$^{T1}\eta_{sp}$ (N/m²)*	*$^{T2}\eta_{sp}$ (N/m²)*	*$^{T3}\eta_{sp}$ (N/m²)*	*$^{T4}\eta_{sp}$ (N/m²)*	*$^{Avg}\eta_{sp}$ (N/m²)*
0.5	405	*E*	16.61	16.59	16.60	16.57	16.59	22.73	22.70	22.71	22.67	22.70
1.0	530	*Z*	7.91	7.92	7.89	7.87	7.90	10.30	10.31	10.27	10.24	10.28
1.5	405	*E*	16.53	16.49	16.48	16.51	16.50	22.61	22.56	22.54	22.59	22.58
2.0	530	*Z*	7.98	7.88	7.97	7.85	7.92	10.40	10.26	10.39	10.21	10.31
2.5	405	*E*	16.59	16.61	16.63	16.58	16.60	22.70	22.73	22.76	22.69	22.72
3.0	530	*Z*	8.03	8.00	8.01	7.99	8.01	10.47	10.43	10.44	10.41	10.44
3.5	405	*E*	16.45	16.49	16.44	16.47	16.46	22.50	22.56	22.49	22.53	22.52
4.0	530	*Z*	8.15	8.14	8.12	8.16	8.14	10.64	10.63	10.60	10.66	10.63
4.5	405	*E*	16.50	16.51	16.49	16.47	16.49	22.57	22.59	22.56	22.53	22.56
5.0	530	*Z*	8.07	8.08	8.05	8.05	8.06	10.53	10.54	10.50	10.50	10.52
5.5	405	*E*	16.41	16.42	16.40	16.41	16.41	22.44	22.46	22.43	22.44	22.44
6.0	530	*Z*	8.05	8.09	8.10	8.08	8.08	10.50	10.56	10.57	10.54	10.54
6.5	405	*E*	16.41	16.38	16.37	16.40	16.39	22.44	22.40	22.39	22.43	22.41
7.0	530	*Z*	8.36	8.38	8.40	8.39	8.38	10.94	10.97	11.00	10.99	10.98
7.5	405	*E*	16.15	16.18	16.19	16.20	16.18	22.07	22.11	22.13	22.14	22.11
8.0	530	*Z*	7.69	7.73	7.70	7.65	7.69	9.99	10.04	10.00	9.93	9.99
8.5	405	*E*	16.10	16.16	16.13	16.15	16.14	22.00	22.09	22.04	22.07	22.05
9.0	530	*Z*	8.24	8.23	8.25	8.31	8.26	10.77	10.76	10.79	10.87	10.80
9.5	405	*E*	16.15	16.11	16.04	16.10	16.10	22.07	22.01	21.91	22.00	22.00
10.0	530	*Z*	8.47	8.51	8.62	8.49	8.52	11.10	11.16	11.31	11.13	11.18

Table 13. Viscosity measurements for foldamer **III** (56 mM) after 10 cycles of irradiation at 405 nm and 530 nm.

Cycle 32 mM	*λ (nm)*	*Isomer*	*T1 (s)*	*T2 (s)*	*T3 (s)*	*T4 (s)*	*Avg T (s)*	*$^{T1}\eta_{sp}$ (N/m²)*	*$^{T2}\eta_{sp}$ (N/m²)*	*$^{T3}\eta_{sp}$ (N/m²)*	*$^{T4}\eta_{sp}$ (N/m²)*	*$^{Avg}\eta_{sp}$ (N/m²)*
0.5	405	*E*	3.01	3.07	3.08	3.04	3.05	3.30	3.39	3.40	3.34	3.36
1.0	530	*Z*	1.52	1.50	1.53	1.51	1.52	1.17	1.14	1.19	1.16	1.16
1.5	405	*E*	3.10	3.12	3.08	3.07	3.09	3.43	3.46	3.40	3.39	3.42
2.0	530	*Z*	1.47	1.44	1.49	1.48	1.47	1.10	1.06	1.13	1.11	1.10
2.5	405	*E*	3.02	3.01	3.00	3.02	3.01	3.31	3.30	3.29	3.31	3.30
3.0	530	*Z*	1.56	1.54	1.58	1.55	1.56	1.23	1.20	1.26	1.21	1.23
3.5	405	*E*	2.99	3.02	2.98	3.00	3.00	3.27	3.31	3.26	3.29	3.28
4.0	530	*Z*	1.47	1.47	1.42	1.45	1.45	1.10	1.10	1.03	1.07	1.08
4.5	405	*E*	3.01	3.02	3.03	3.04	3.03	3.30	3.31	3.33	3.34	3.32
5.0	530	*Z*	1.52	1.58	1.53	1.52	1.54	1.17	1.26	1.19	1.17	1.20
5.5	405	*E*	2.95	2.95	2.90	2.99	2.95	3.21	3.21	3.14	3.27	3.21
6.0	530	*Z*	1.47	1.49	1.50	1.44	1.48	1.10	1.13	1.14	1.06	1.11
6.5	405	*E*	3.01	3.00	3.02	3.03	3.02	3.30	3.29	3.31	3.33	3.31
7.0	530	*Z*	1.47	1.49	1.44	1.45	1.46	1.10	1.13	1.06	1.07	1.09
7.5	405	*E*	2.97	2.93	2.89	3.00	2.95	3.24	3.19	3.13	3.29	3.21
8.0	530	*Z*	1.55	1.53	1.49	1.51	1.52	1.21	1.19	1.13	1.16	1.17
8.5	405	*E*	2.92	2.90	2.93	2.91	2.92	3.17	3.14	3.19	3.16	3.16
9.0	530	*Z*	1.56	1.55	1.53	1.51	1.54	1.23	1.21	1.19	1.16	1.20
9.5	405	*E*	2.96	2.97	2.93	2.94	2.95	3.23	3.24	3.19	3.20	3.21
10.0	530	*Z*	1.58	1.60	1.61	1.62	1.60	1.26	1.29	1.30	1.31	1.29

Table 14. Viscosity measurements for foldamer **III** (32 mM) after 10 cycles of irradiation at 405 nm and 530 nm.

Cycle 4 mM	*λ (nm)*	*Isomer*	*T1 (s)*	*T2 (s)*	*T3 (s)*	*T4 (s)*	*Avg T (s)*	*$^{T1}\eta_{sp}$ (N/m^2)*	*$^{T2}\eta_{sp}$ (N/m^2)*	*$^{T3}\eta_{sp}$ (N/m^2)*	*$^{T4}\eta_{sp}$ (N/m^2)*	*$^{Avg}\eta_{sp}$ (N/m^2)*
0.5	405	*E*	0.73	0.69	0.70	0.71	0.71	0.04	-0.01	0.00	0.01	0.01
1.0	530	*Z*	0.71	0.70	0.70	0.69	0.70	0.01	0.00	0.00	-0.01	0.00
1.5	405	*E*	0.69	0.75	0.69	0.68	0.70	-0.87	0.07	-0.01	-0.03	0.00
2.0	530	*Z*	0.76	0.73	0.76	0.73	0.75	-0.86	0.04	0.09	0.04	0.06
2.5	405	*E*	0.72	0.76	0.74	0.71	0.73	-0.86	0.09	0.06	0.01	0.05
3.0	530	*Z*	0.74	0.69	0.68	0.73	0.71	-0.86	-0.01	-0.03	0.04	0.01
3.5	405	*E*	0.72	0.68	0.69	0.70	0.70	-0.86	-0.03	-0.01	0.00	0.00
4.0	530	*Z*	0.78	0.72	0.79	0.79	0.77	-0.85	0.03	0.13	0.13	0.10
4.5	405	*E*	0.76	0.73	0.76	0.74	0.75	-0.86	0.04	0.09	0.06	0.07
5.0	530	*Z*	0.77	0.79	0.78	0.77	0.78	-0.85	0.13	0.11	0.10	0.11
5.5	405	*E*	0.81	0.82	0.77	0.75	0.79	-0.85	0.17	0.10	0.07	0.13
6.0	530	*Z*	0.78	0.80	0.79	0.71	0.77	-0.85	0.14	0.13	0.01	0.10
6.5	405	*E*	0.79	0.82	0.77	0.85	0.81	-0.85	0.17	0.10	0.21	0.15
7.0	530	*Z*	0.75	0.83	0.75	0.77	0.78	-0.86	0.19	0.07	0.10	0.11
7.5	405	*E*	0.73	0.79	0.76	0.81	0.77	-0.86	0.13	0.09	0.16	0.10
8.0	530	*Z*	0.80	0.79	0.82	0.83	0.81	-0.85	0.13	0.17	0.19	0.16
8.5	405	*E*	0.76	0.78	0.73	0.68	0.74	-0.86	0.11	0.04	-0.03	0.05
9.0	530	*Z*	0.77	0.81	0.80	0.83	0.80	-0.85	0.16	0.14	0.19	0.15
9.5	405	*E*	0.78	0.86	0.82	0.80	0.82	-0.85	0.23	0.17	0.14	0.16
10.0	530	*Z*	0.81	0.80	0.77	0.83	0.80	-0.85	0.14	0.10	0.19	0.15

Table 15. Viscosity measurements for foldamer **III** (4 mM) after 10 cycles of irradiation at 405 nm and 530 nm.

7. Molecular Modelling

Calculations were performed for both the *Z* and *E* forms of foldamer **II** (with 2-ethylhexylgroups substituted for methyl groups) using the xTB 6.6.0 package[8] with the GFN2-xTB extended semi-empirical tight-binding model[9] and the ALPB implicit solvation[10] using chloroform as the solvent. Initial monomer and dimer structures were input as starting structures for molecular dynamics simulations without constraints although no conversion between *Z* and *E* conformations was observed. Molecular dynamics simulations were performed using NVT ensembles for a minimum of 1000 ps with a step size of 4 fs and a temperature of 298.5 K with all other parameters set as default.

After a sensible monomer or dimer had formed, and was stable during the MD trajectory, a sample structure was taken from each of the trajectories and optimised using the tight optimization level before a Hessian matrix was determined by invoking the --hess command line argument. For both the monomer and dimer for both the *Z* and *E* forms, the structures determined were stable with 0 imaginary frequencies. The information from this vibrational calculation was then used to determine the total free energy of each monomer and dimer at 298.5 K.

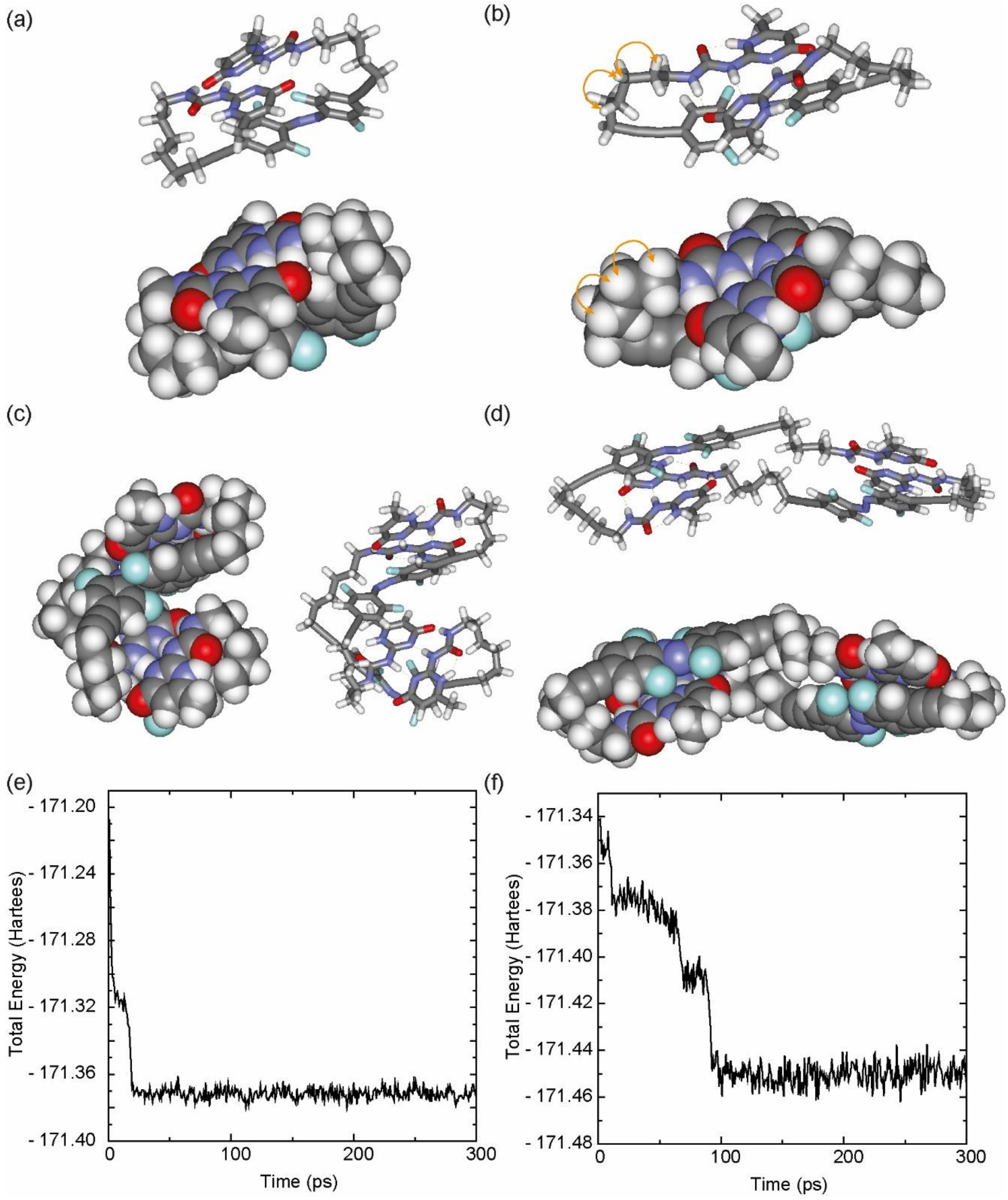

Figure S3. Molecular Modelling on *Z* and *E* monomers and dimers of UPy·UPy foldamer **II** lacking 2-ethylhexyl chains: (a) *Z* UPy·UPy foldamer **II** monomer (top: stick, bottom: CPK); (b) *E* UPy·UPy foldamer **II** monomer (top: stick, bottom: CPK, orange arrows denote gauche interactions); (c) *Z* UPy·UPy foldamer **II** dimer (left: stick, right: CPK); (d) *E* UPy·UPy foldamer **II** dimer (top: stick, bottom: CPK, orange arrows denote gauche interactions); (e) plot of total electronic energy vs time for the MD trajectories for both the *Z* monomer and (f) plot of total electronic energy vs time for the MD trajectories for both the *E* monomer.

The free energy determined from the vibrational calculations indicates that the *Z* monomer is the most stable and that the corresponding *Z* dimer is ~ 17 kJ mol^{-1} less stable, whilst the *E* monomer is ~ 18 kJ mol^{-1} less stable and the *E* dimer ~ 21 kJ mol^{-1} less stable. The data qualitatively agree with the observations from DOSY NMR analyses in that the intramolecular folding in *Z* UPy·UPy foldamer **II** is strongly favoured, whereas the difference in stability between intramolecular folding and intermolecular dimerization in *E* UPy·UPy foldamer **II** is less pronounced. A more quantitative analyses to account for conformational entropic differences is impractical, although the clear presence of gauche interactions for *E* UPy·UPy foldamer **II** monomer imply significant strain for intramolecular folding and hydrogen-bonding to occur. Finally, whilst the exact starting energy would be decided by the initial configuration, the trajectories in panels (e) and (f) emphasise the stability of the *Z* monomer which self-associates almost four times quicker than the *E* form which appears to get stuck at several intermediate steps before eventually finding its most stable monomeric conformation.

8. NMR Spectra

8.1 ^{1}H NMR Spectra

1-(4-Bromo-2,6-difluorophenyl)-2-(2,6-difluoro-4-iodophenyl)diazene 3

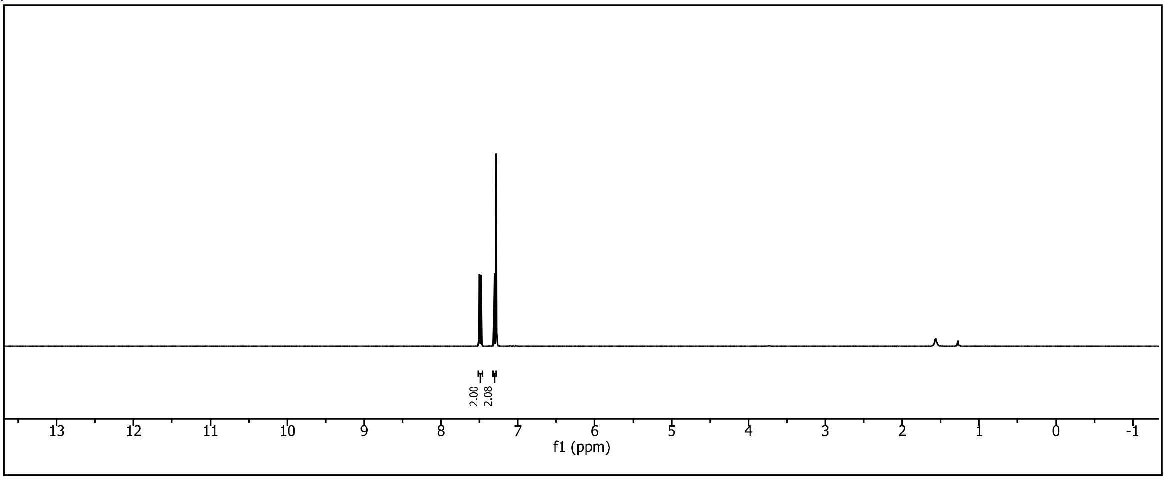

8-Noynoic acid 5

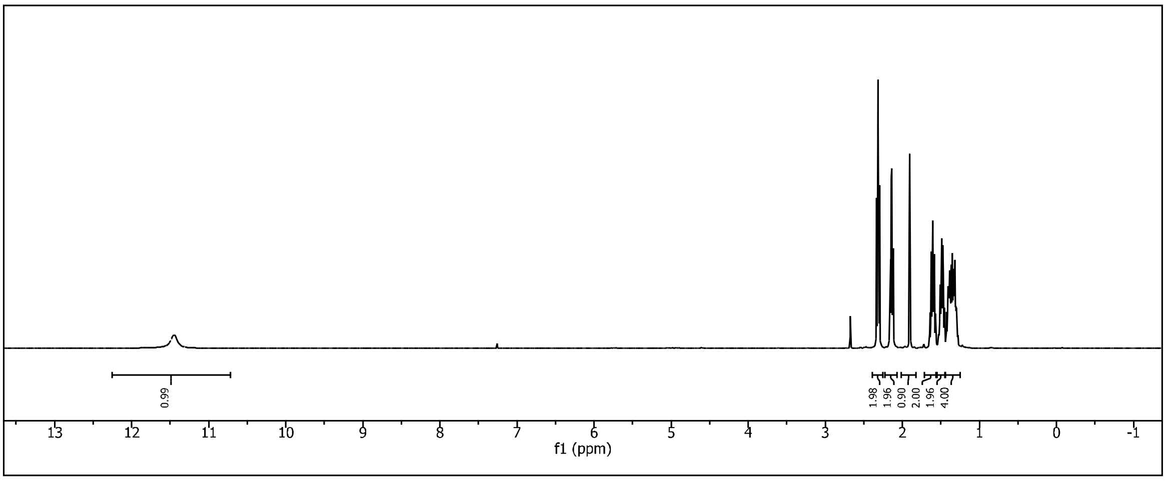

1-(Oct-7-yn-1-yl)-3-(pyridin-2-yl)urea 6

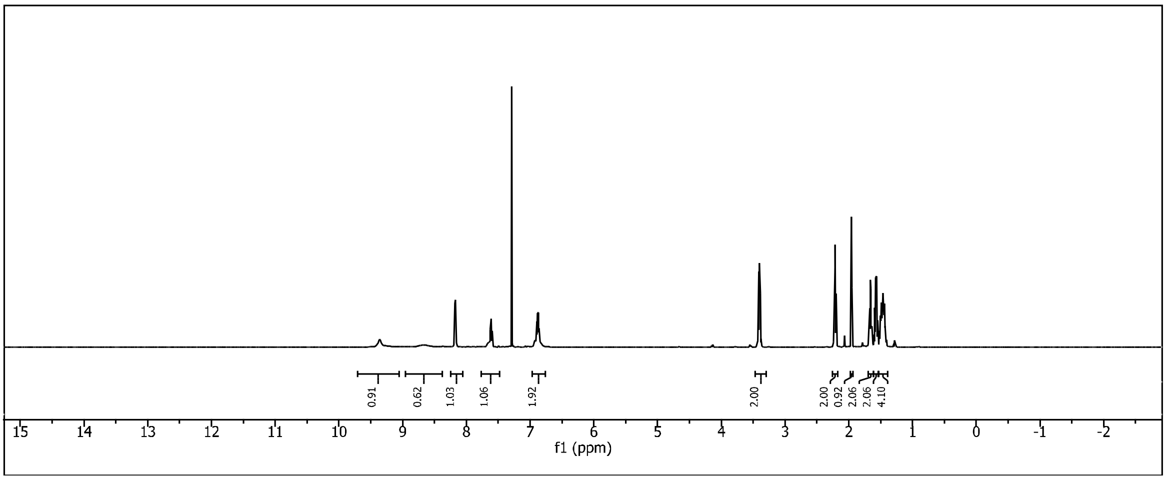

1,8-Naphthyridin-2-amine 8

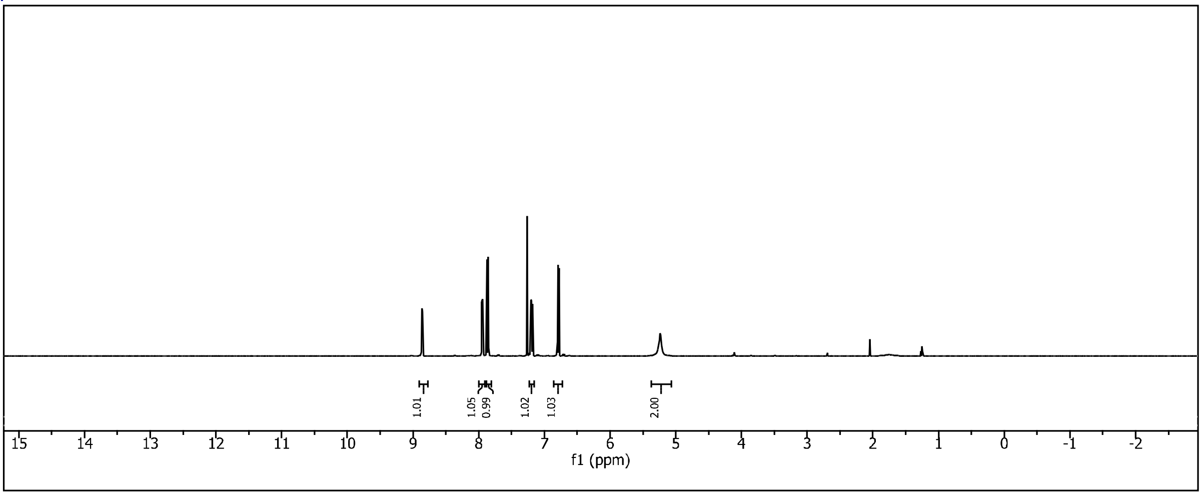

N-(1,8-Naphthyridin-2-yl)non-8-ynamide 9

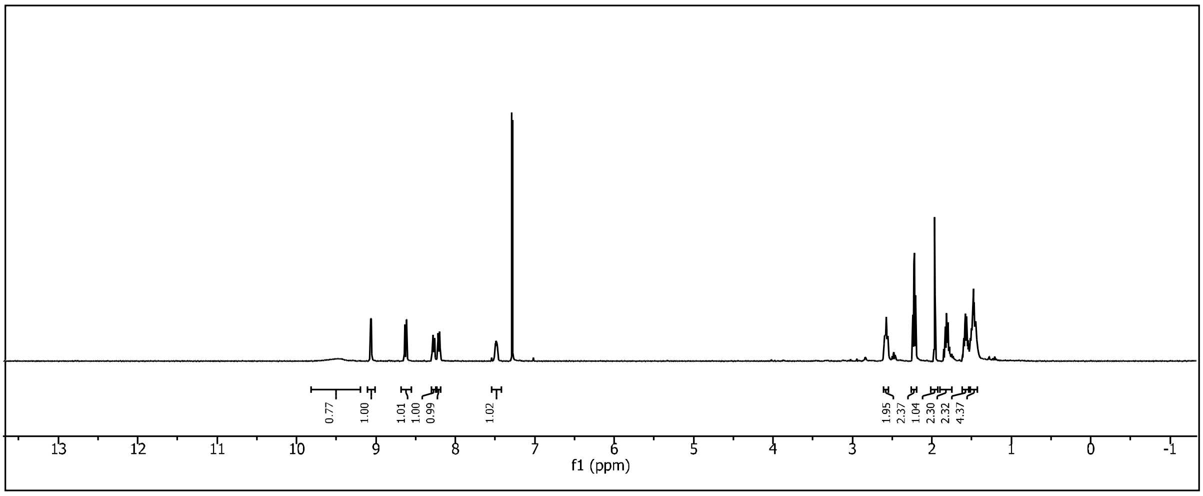

(E)-1- (8- (4-((4-Bromo -2,6- difluorophenyl)diazenyl)-3,5-difluorophenyl)oct7-yn-1-yl)-3-(pyridin-2-yl)urea 10

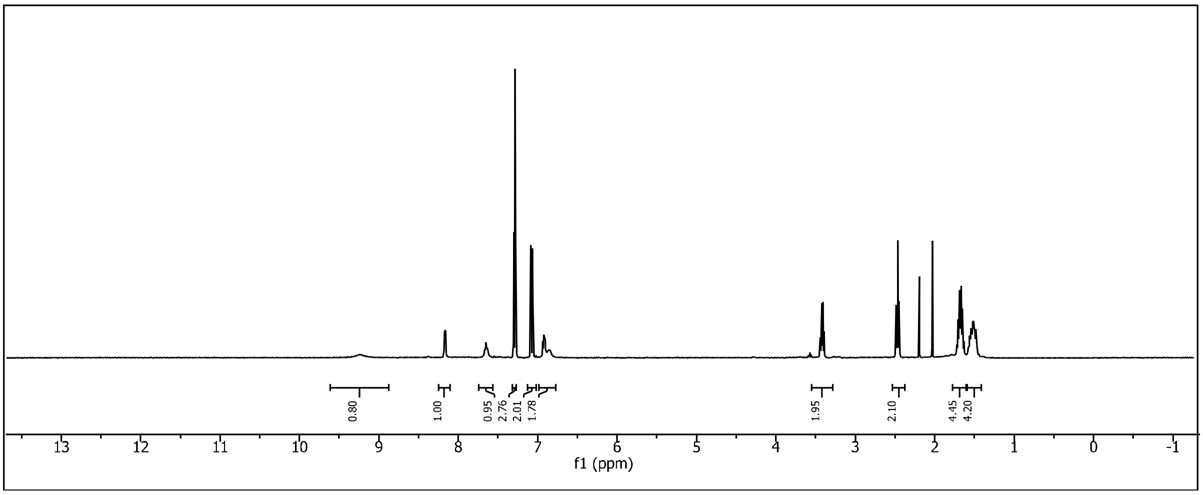

1-(Oct-7-yn-1-yl)-N-(4-(1-ethylpentyl)-1,6-dihydro-6-oxo-2-pyrimidinyl))urea 12

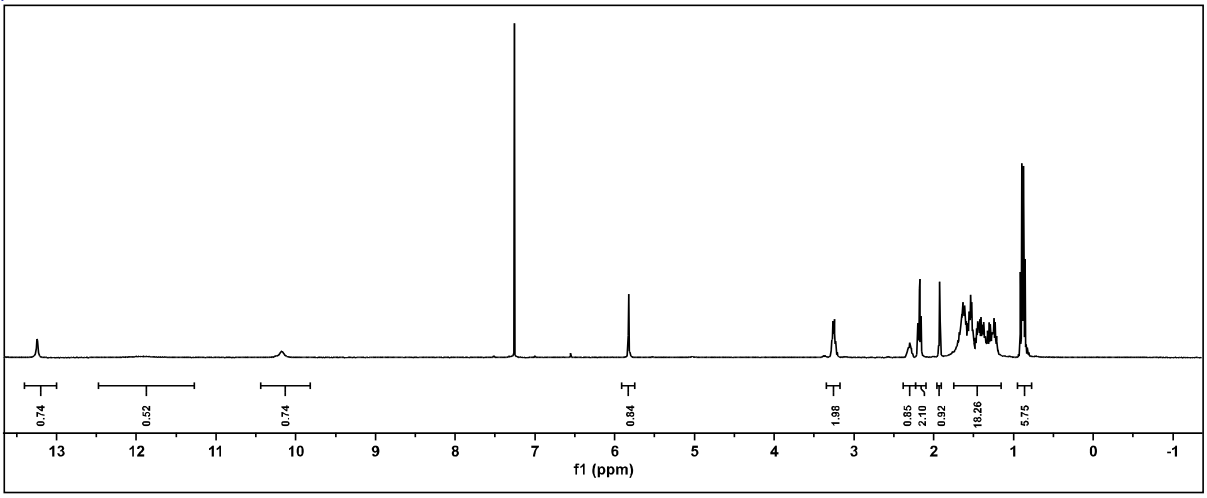

1-(8-(4-((4-Bromo -2,6- difluorophenyl) diazenyl)- 3,5-difluorophenyl) oct7-yn-1-yl)-N-(4-(1-ethylpentyl)-1,6-dihydro-6-oxo-2-pyrimidinyl))urea) 15

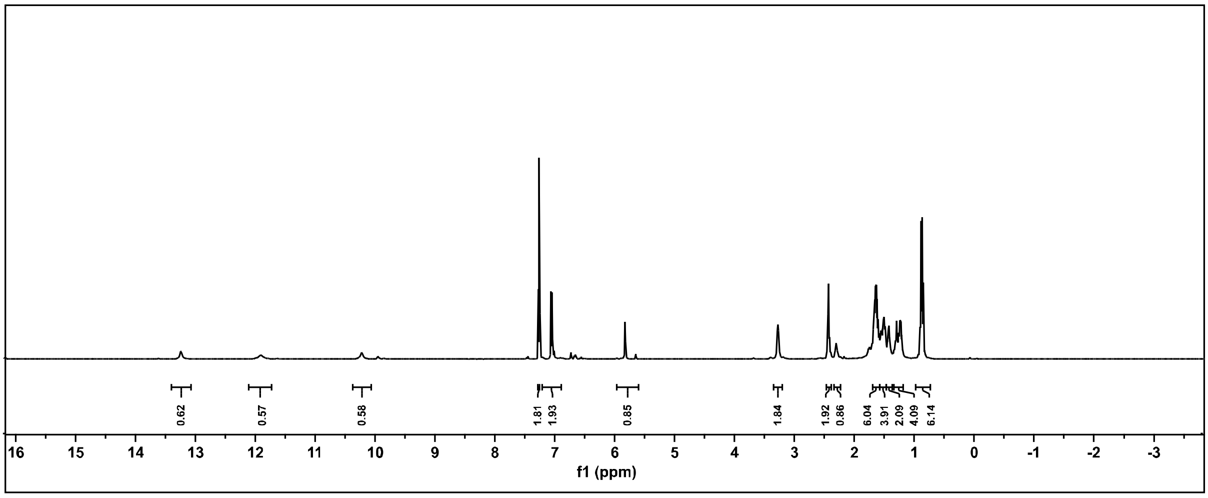

7-Hydroxy-1,8-naphthyridin-2-amine 18

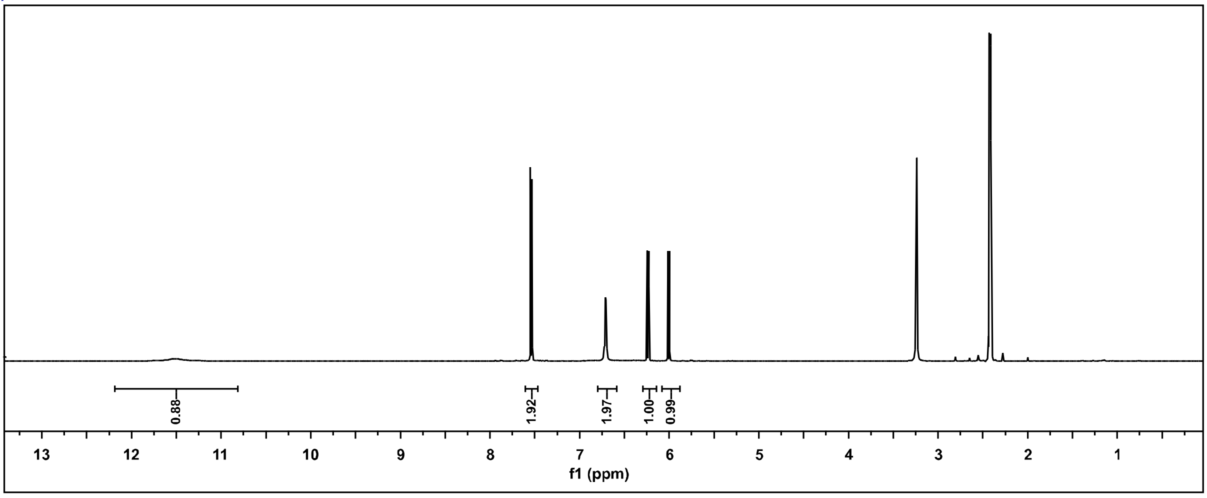

7-(2-ethyl-hexanoyl)-amino-8H-(7-oxo-[1,8]-naphthyridine-2-yl) 19

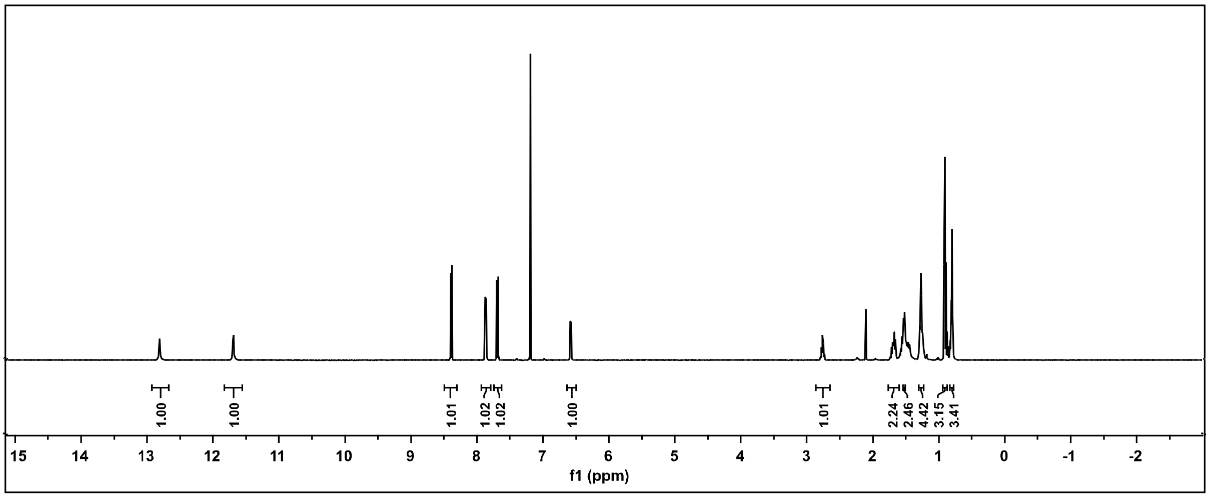

7-(2-ethyl-hexanoyl)-amino-7-chloro-[1,8]-naphthyridine-2-yl 20

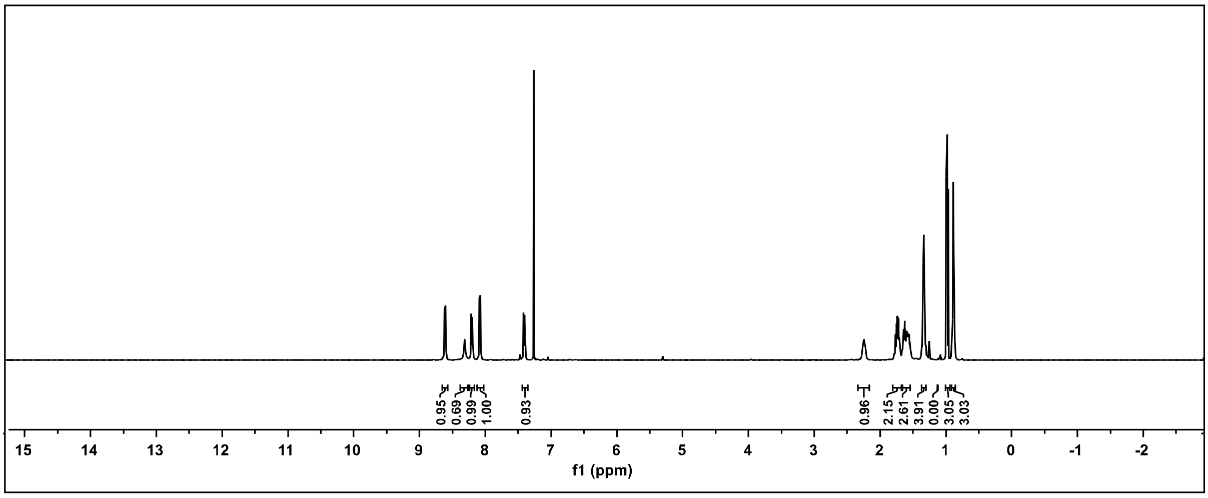

N-(7-Amino-1,8-naphthyridin-2-yl)-2-ethylhexanamide 21

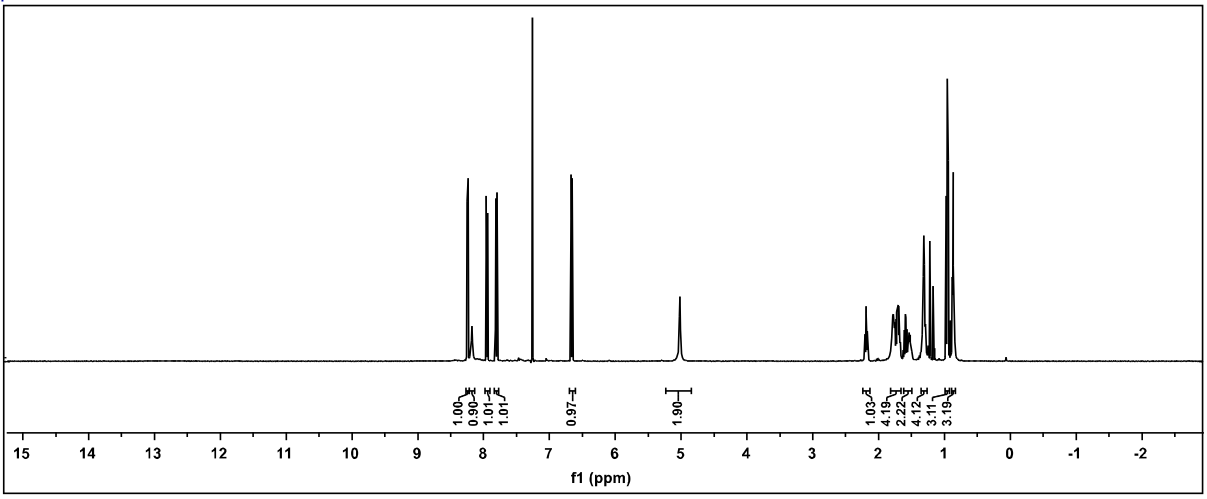

N-((1,8-Naphthyridin-2-yl)-2-ethylhexanamide)non-8-ynamide 22

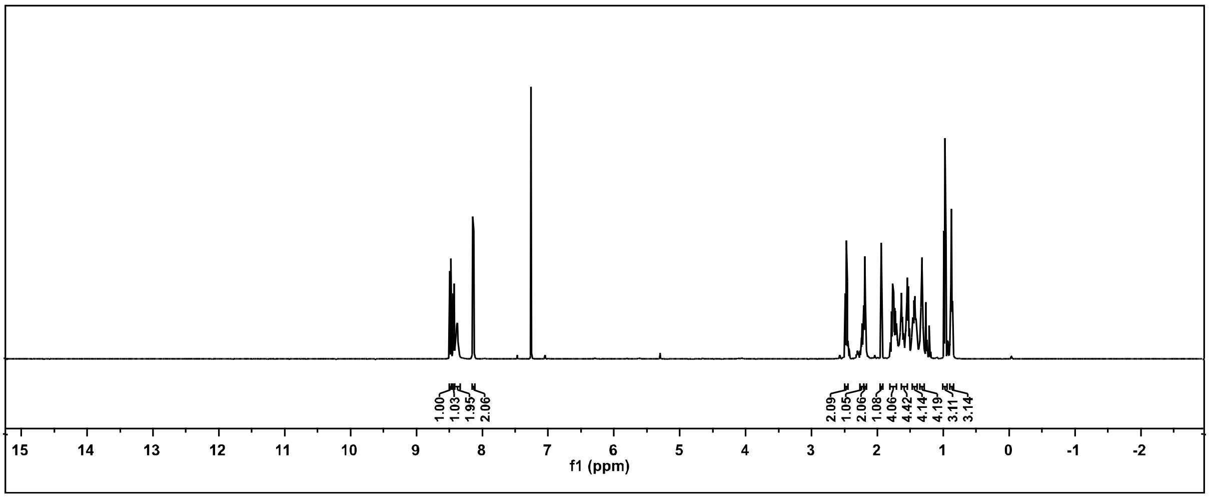

(*E*)-9-(4-((2,6-Difluoro-4-(8-(3-(pyridin-2-yl)ureido)oct-1-yn-1-yl)phenyl)diazenyl)-3,5-difluorophenyl)-N-(1,8- naphthyridin-2-yl)non-8-ynamide Foldamer I

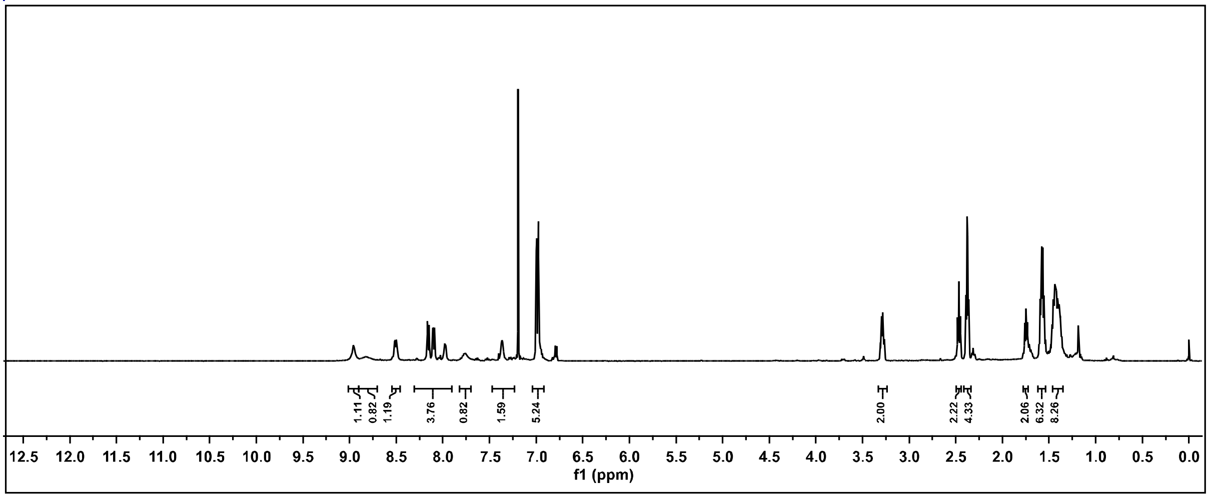

(*Z*)-9-(4-((2,6-Difluoro-4-(8-(3-(pyridin-2-yl)ureido)oct-1-yn-1-yl)phenyl)diazenyl)-3,5-difluorophenyl)-N-(1,8- naphthyridin-2-yl)non-8-ynamide Foldamer I

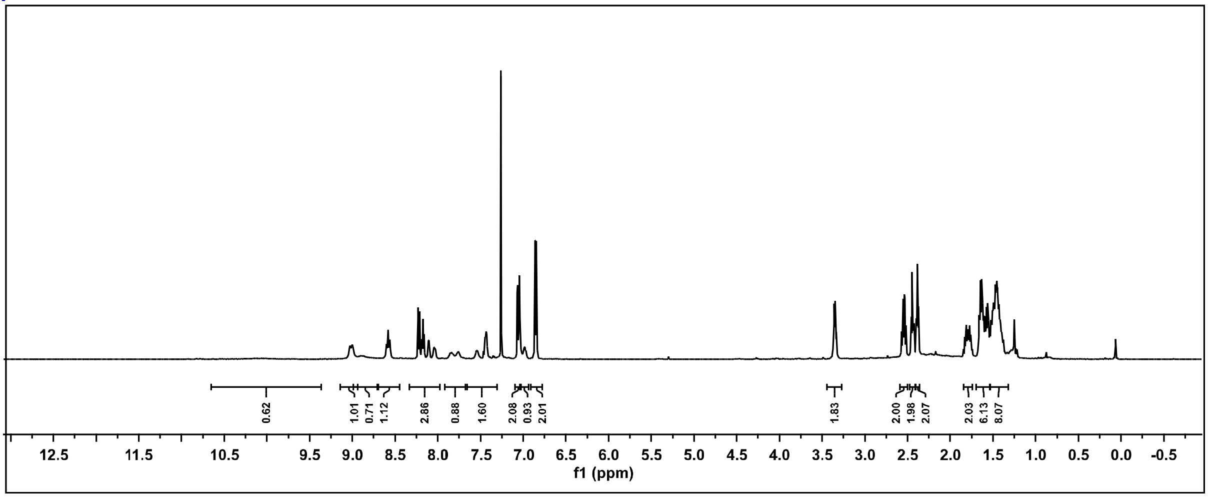

(*E*)-1,2-Bis(2,6-difluoro-4-1-(Oct-7-yn-1-yl)-N-(4-(1-ethylpentyl)-1,6-dihydro-6-oxo-2-pyrimidinyl))urea)diazene Foldamer II

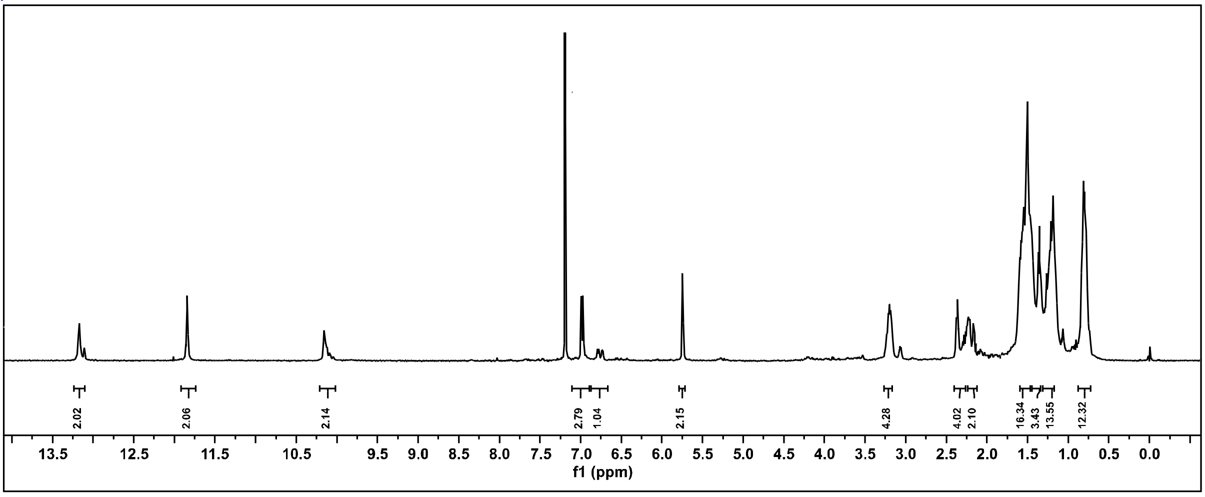

(*Z*)-1,2-Bis(2,6-difluoro-4-1-(Oct-7-yn-1-yl)-N-(4-(1-ethylpentyl)-1,6-dihydro-6-oxo-2-pyrimidinyl))urea)diazene Foldamer II

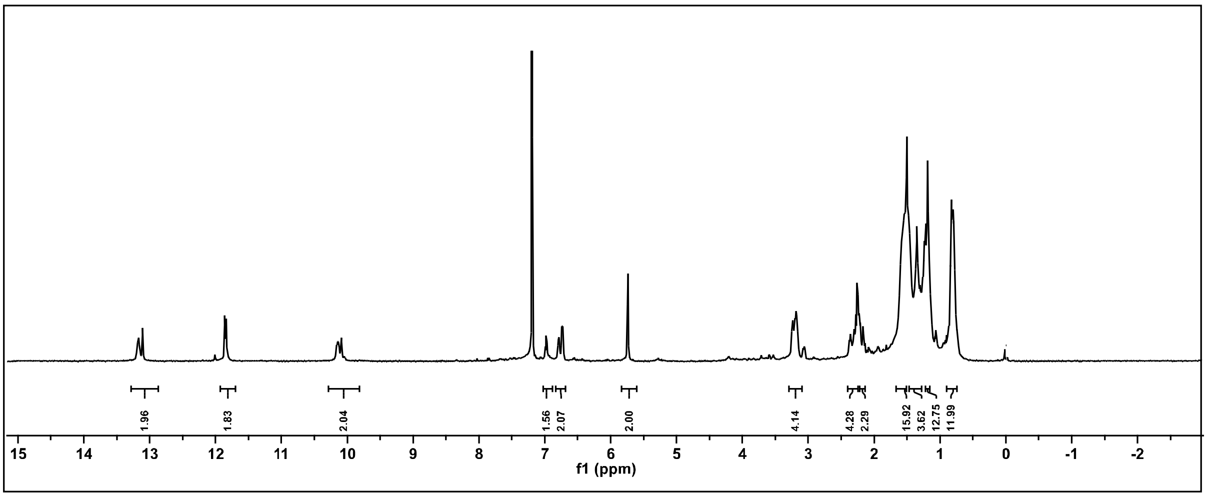

(*E*)- 9- (4- ((2,6-Difluoro - 4 - (oct-7-yn-1-yl) -N- (4-(1-ethylpentyl) - 1,6 - dihydro-6-oxo -2-pyrimidinyl)) urea) phenyl) diazenyl) - 3,5 - difluorophenyl) -N- ((1,8-naphthyridin-2-yl)-2-ethylhexanamide)non-8-ynamide Foldamer III

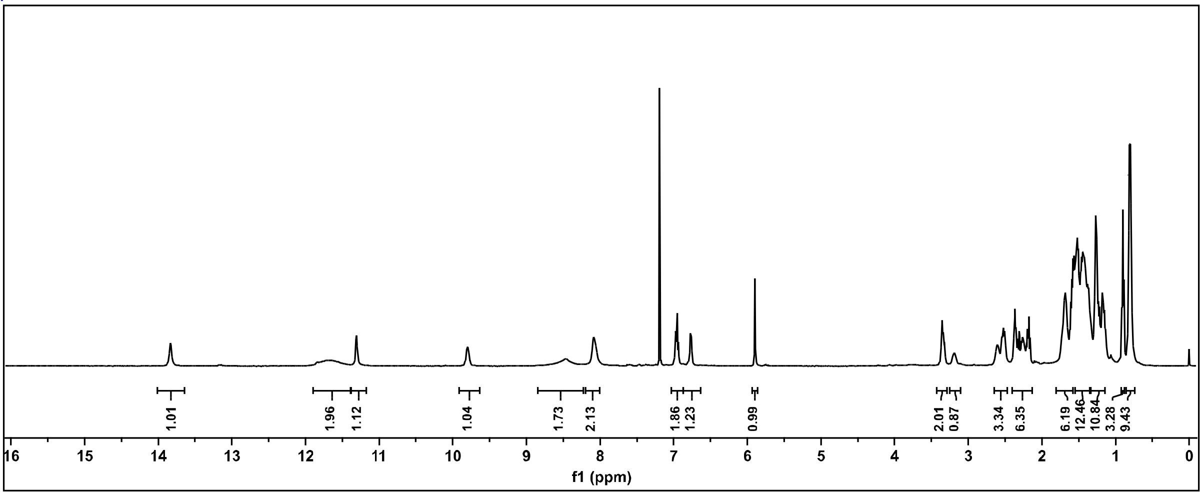

(*Z*)- 9- (4- ((2,6-Difluoro - 4 - (oct-7-yn-1-yl) -N- (4-(1-ethylpentyl) - 1,6 - dihydro-6-oxo -2-pyrimidinyl)) urea) phenyl) diazenyl) - 3,5 - difluorophenyl) -N- ((1,8-naphthyridin-2-yl)-2-ethylhexanamide)non-8-ynamide Foldamer III

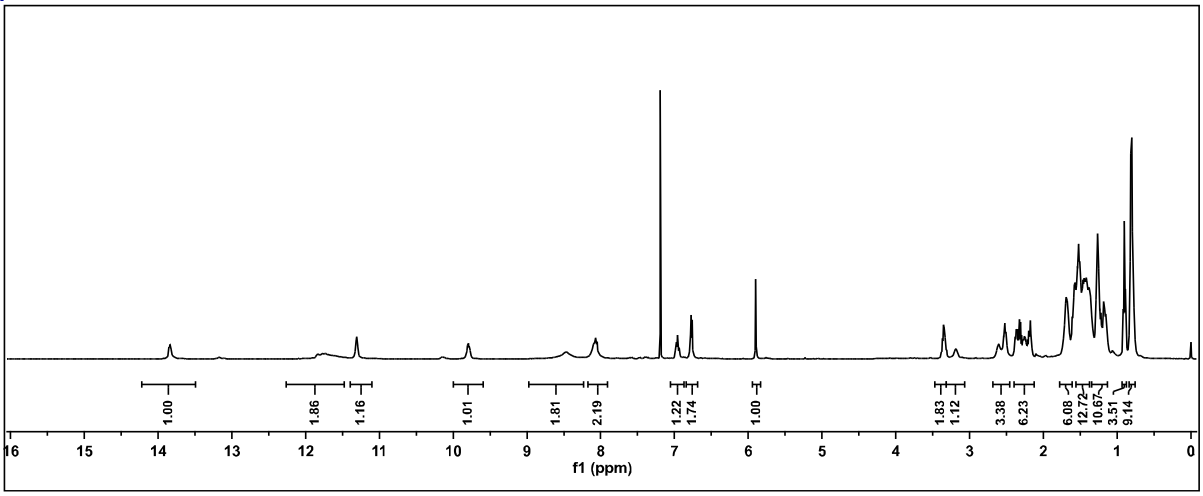

8.2 ^{13}C NMR Spectra

1-(4-Bromo-2,6-difluorophenyl)-2-(2,6-difluoro-4-iodophenyl)diazene 3

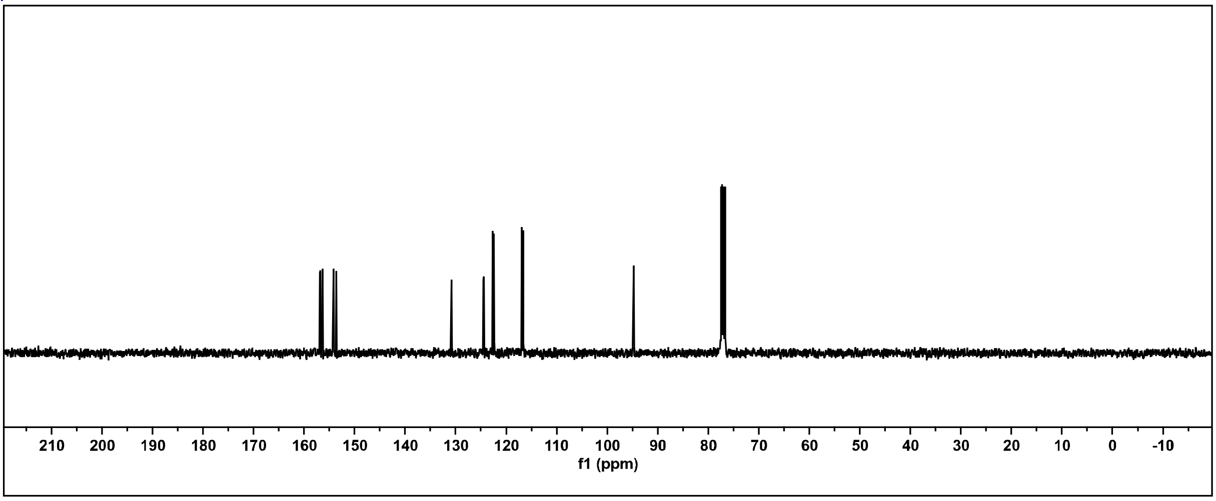

8-Noynoic acid 5

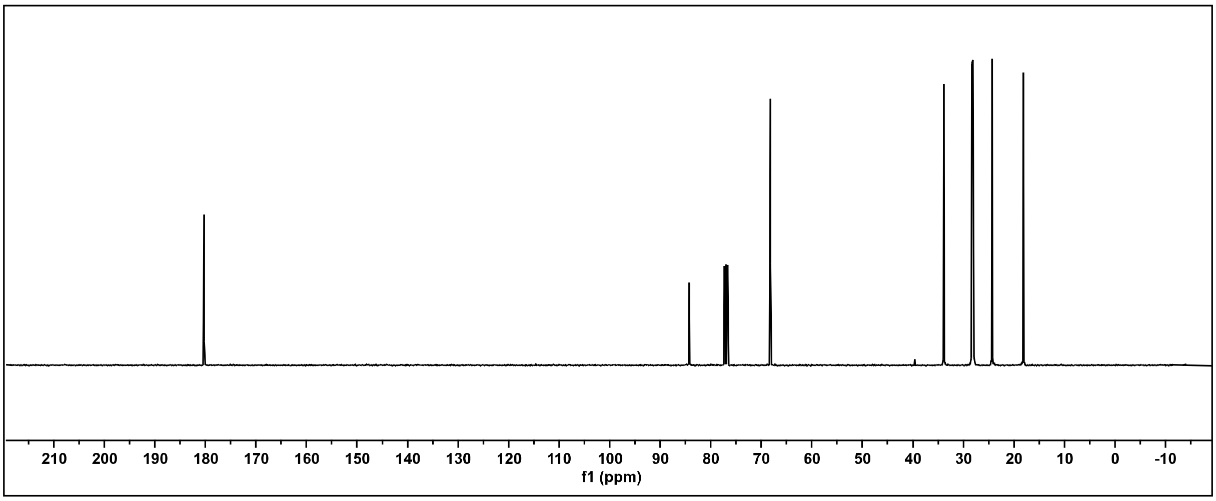

1-(Oct-7-yn-1-yl)-3-(pyridin-2-yl)urea 6

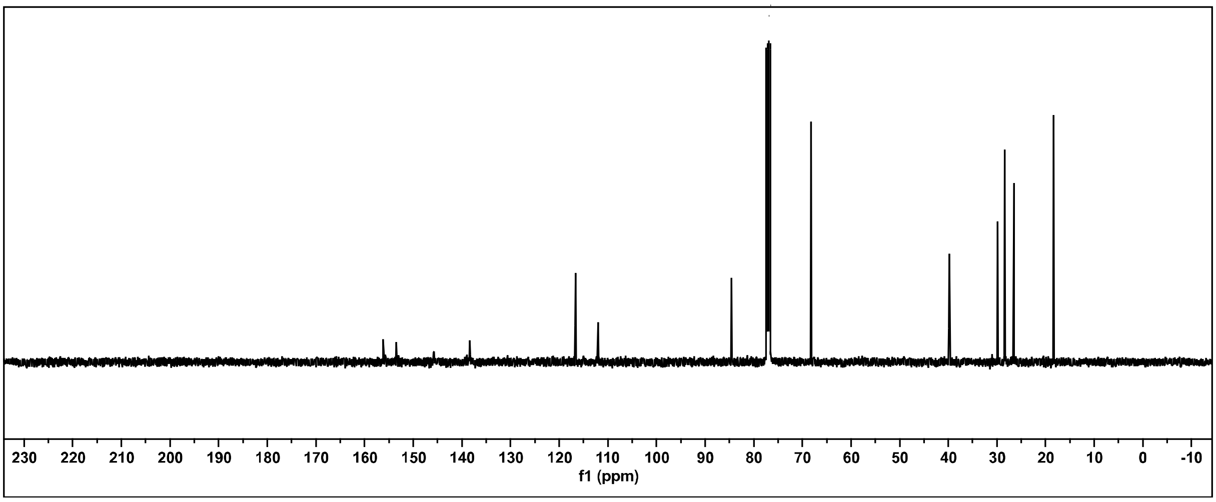

1,8-Naphthyridin-2-amine 8

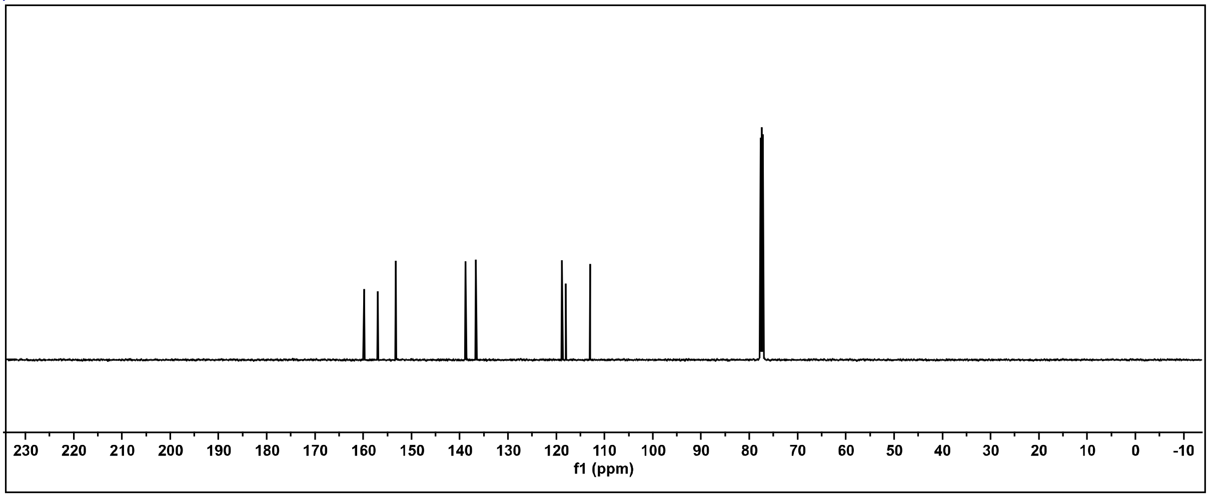

N-(1,8-Naphthyridin-2-yl)non-8-ynamide 9

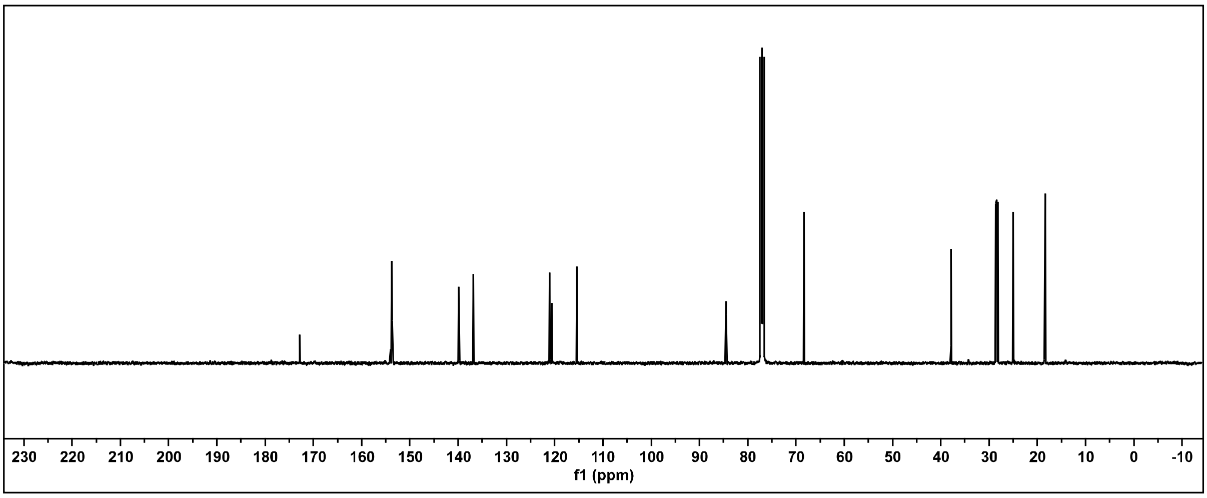

(E)-1- (8- (4-((4-Bromo -2,6- difluorophenyl)diazenyl)-3,5-difluorophenyl)oct7-yn-1-yl)-3-(pyridin-2-yl)urea 10

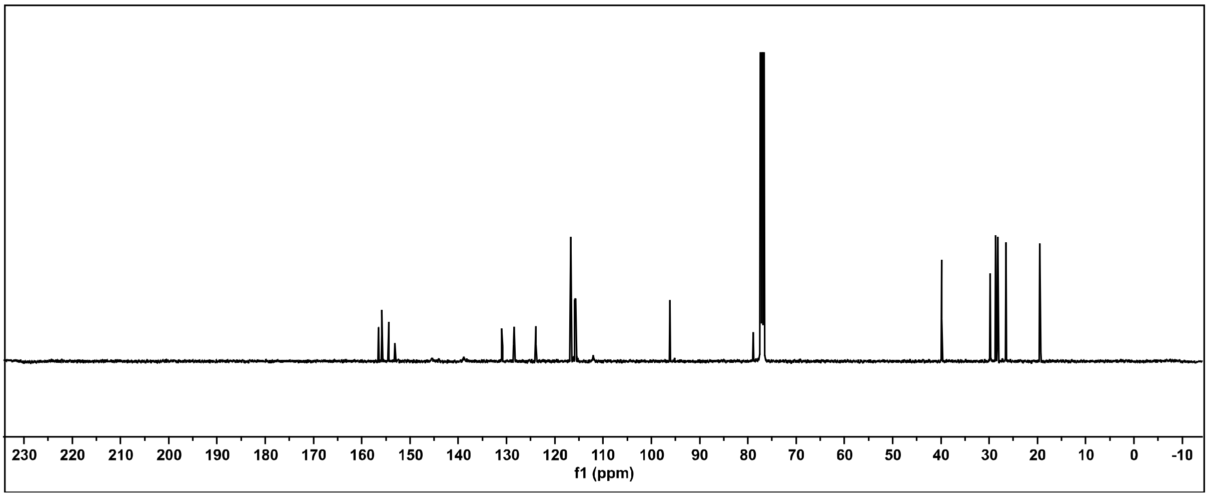

1-(Oct-7-yn-1-yl)-N-(4-(1-ethylpentyl)-1,6-dihydro-6-oxo-2-pyrimidinyl))urea 12

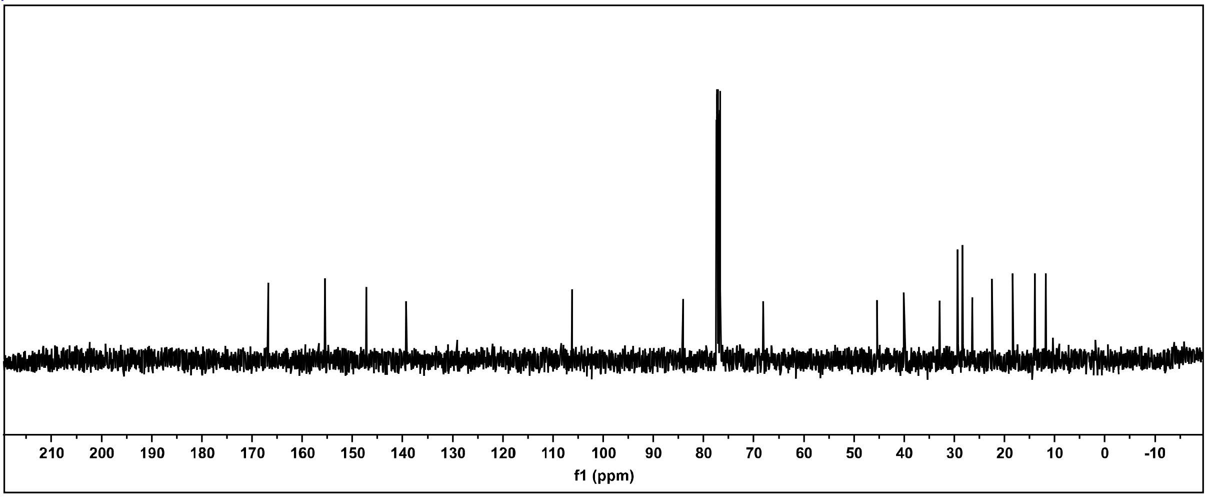

1-(8-(4-((4-Bromo -2,6- difluorophenyl) diazenyl)- 3,5-difluorophenyl) oct7-yn-1-yl)-N-(4-(1-ethylpentyl)-1,6-dihydro-6-oxo-2-pyrimidinyl))urea) 15

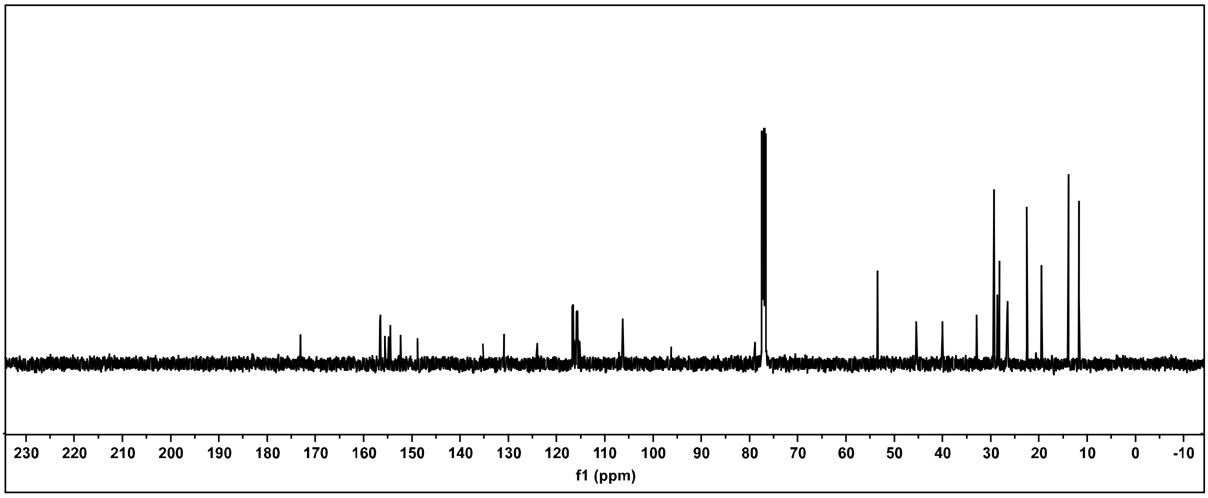

7-Hydroxy-1,8-naphthyridin-2-amine 18

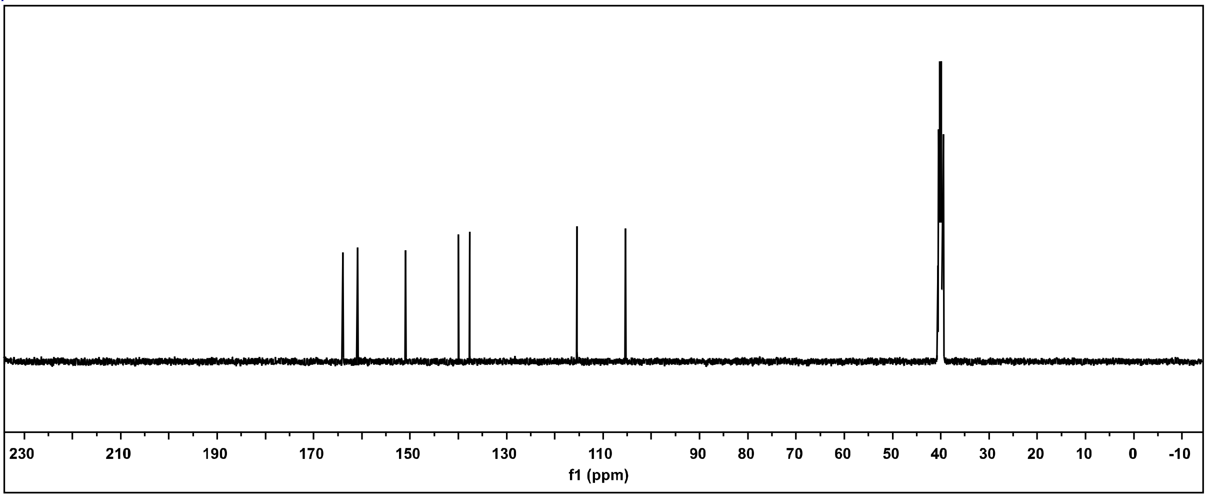

7-(2-ethyl-hexanoyl)-amino-8H-(7-oxo-[1,8]-naphthyridine-2-yl) 19

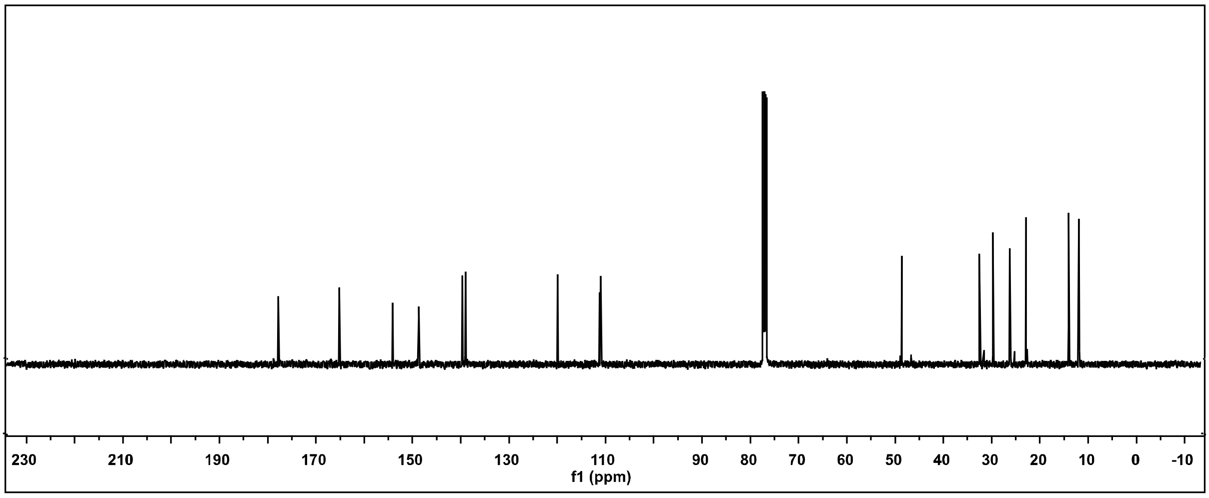

7-(2-ethyl-hexanoyl)-amino-7-chloro-[1,8]-naphthyridine-2-yl 20

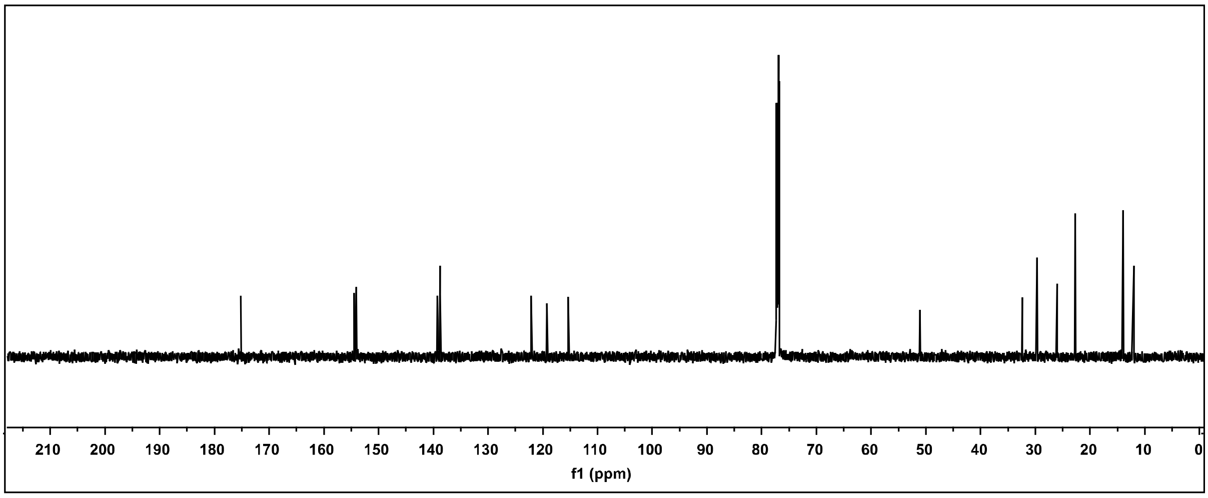

N-(7-Amino-1,8-naphthyridin-2-yl)-2-ethylhexanamide 21

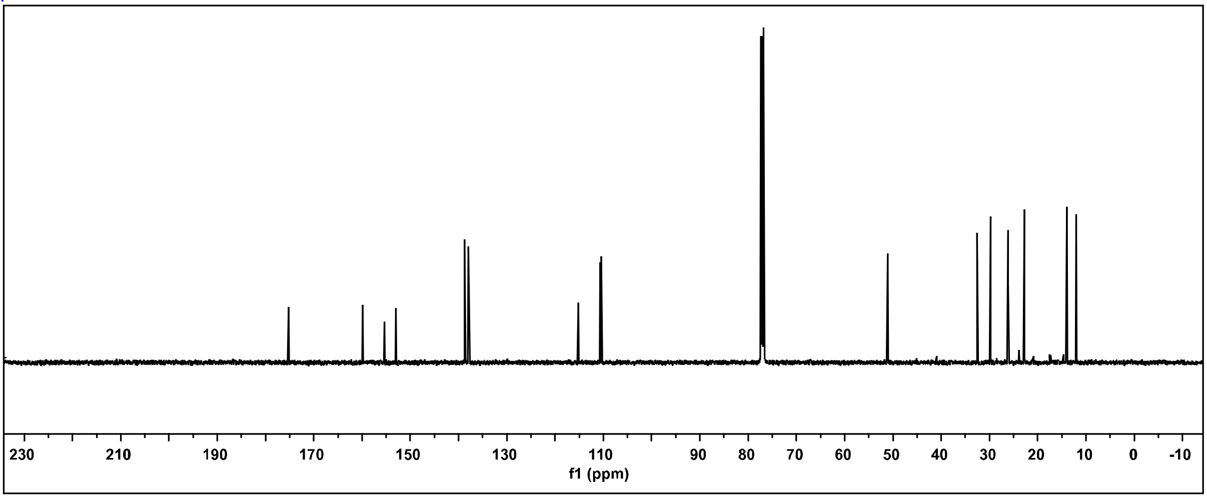

N-((1,8-Naphthyridin-2-yl)-2-ethylhexanamide)non-8-ynamide 22

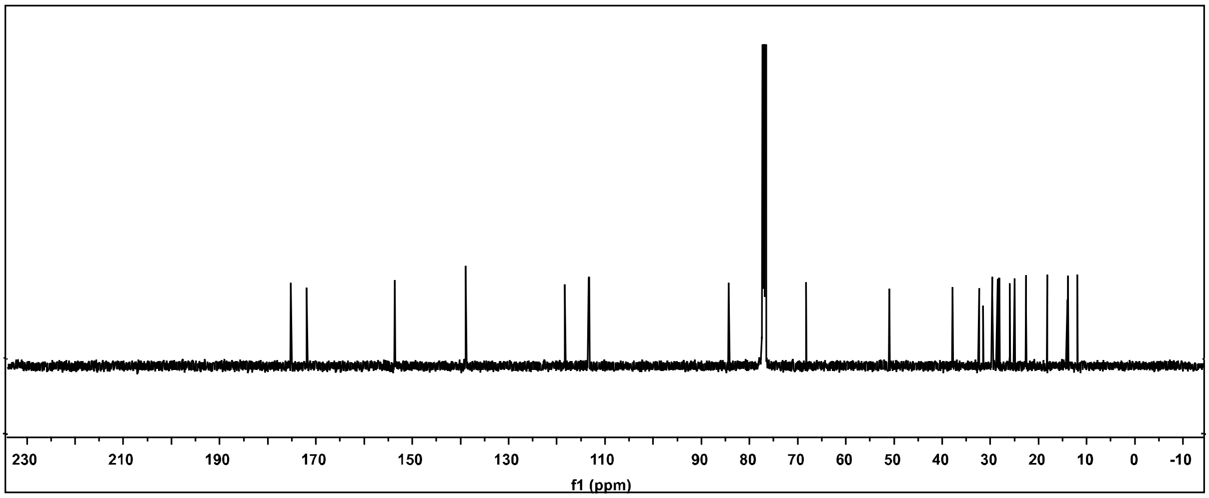

9-(4-((2,6-Difluoro-4-(8-(3-(pyridin-2-yl)ureido)oct-1-yn-1-yl)phenyl)diazenyl)-3,5-difluorophenyl)-N-(1,8- naphthyridin-2-yl)non-8-ynamide Foldamer I

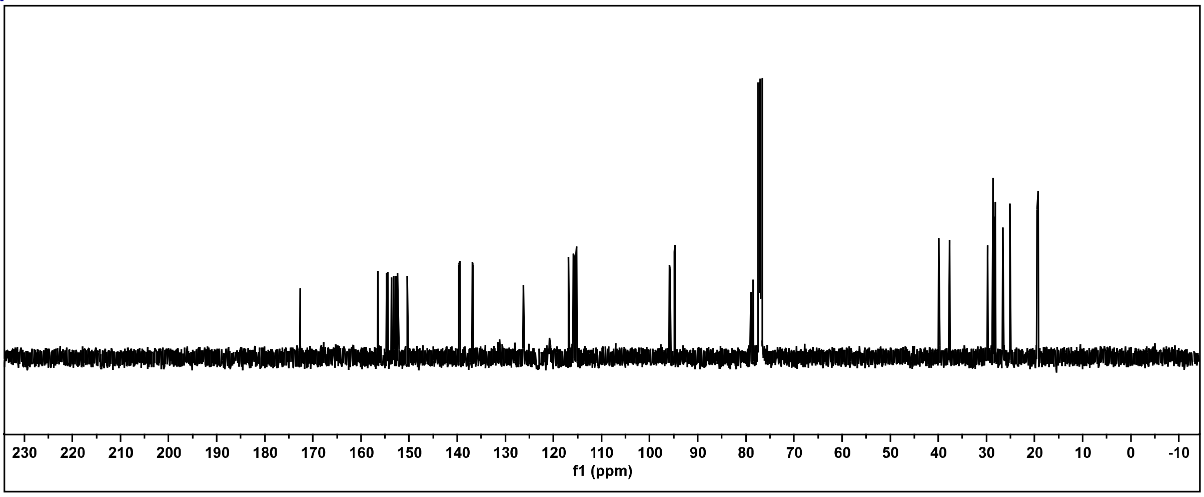

1,2-Bis(2,6-difluoro-4-1-(Oct-7-yn-1-yl)-N-(4-(1-ethylpentyl)-1,6-dihydro-6-oxo-2-pyrimidinyl))urea)diazene Foldamer II

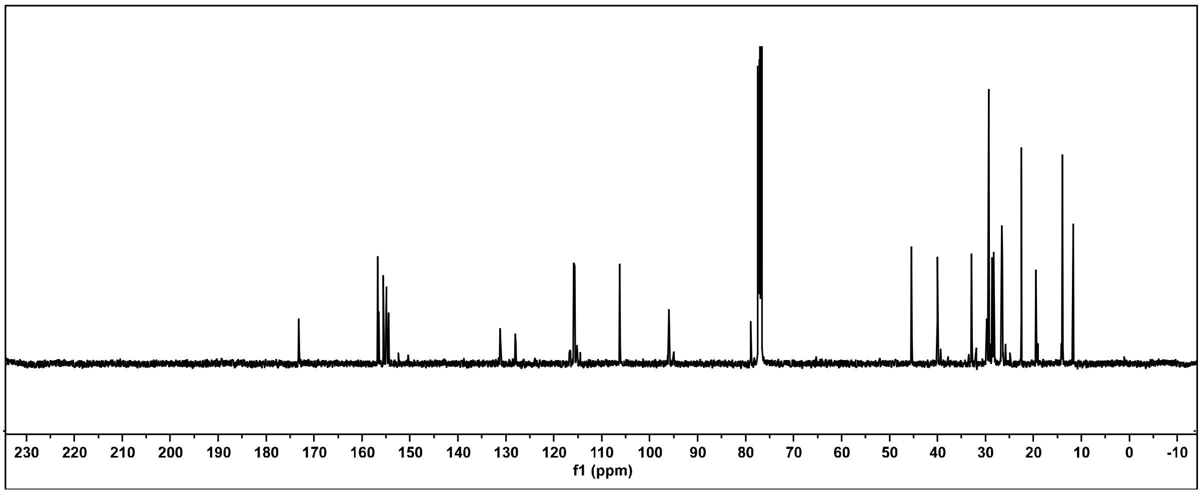

9- (4- ((2,6-Difluoro - 4 - (oct-7-yn-1-yl) -N- (4-(1-ethylpentyl) - 1,6 - dihydro-6-oxo -2-pyrimidinyl)) urea) phenyl) diazenyl) - 3,5 - difluorophenyl) -N- ((1,8-naphthyridin-2-yl)-2-ethylhexanamide)non-8-ynamide Foldamer III

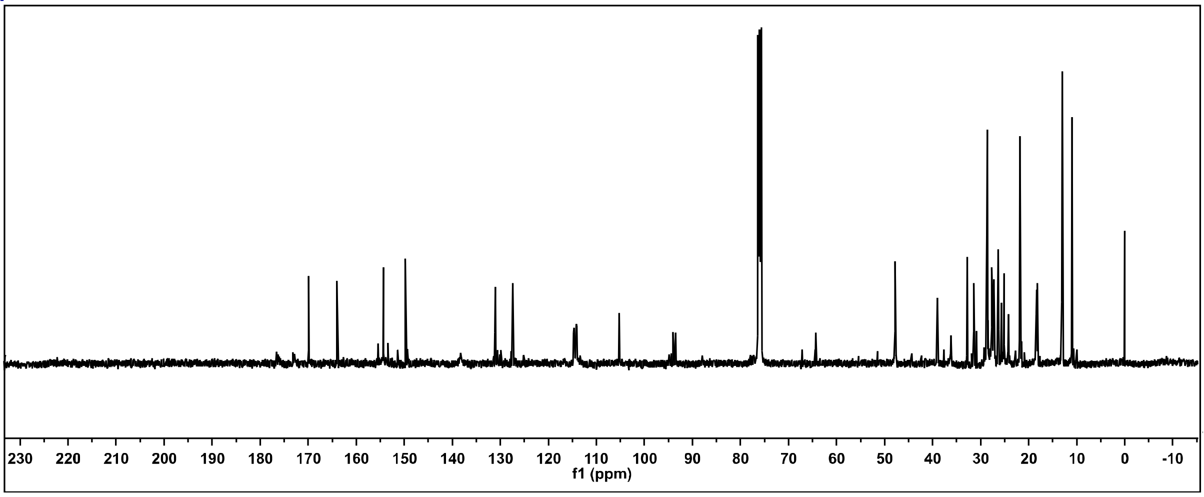

8.3 ^{19}F NMR Spectra

1-(4-Bromo-2,6-difluorophenyl)-2-(2,6-difluoro-4-iodophenyl)diazene 3

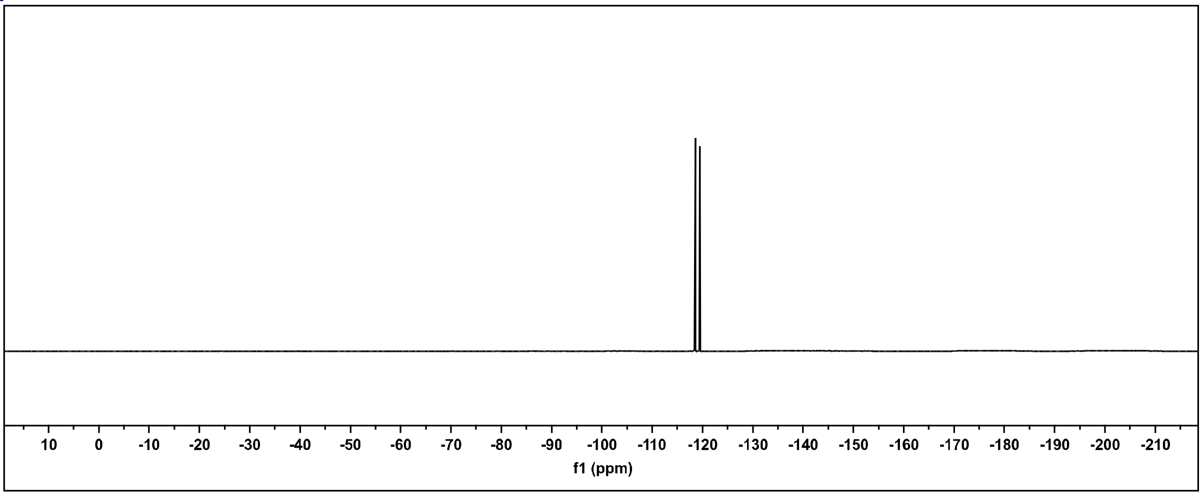

(E)-1- (8- (4-((4-Bromo -2,6- difluorophenyl)diazenyl)-3,5-difluorophenyl)oct7-yn-1-yl)-3-(pyridin-2-yl)urea 10

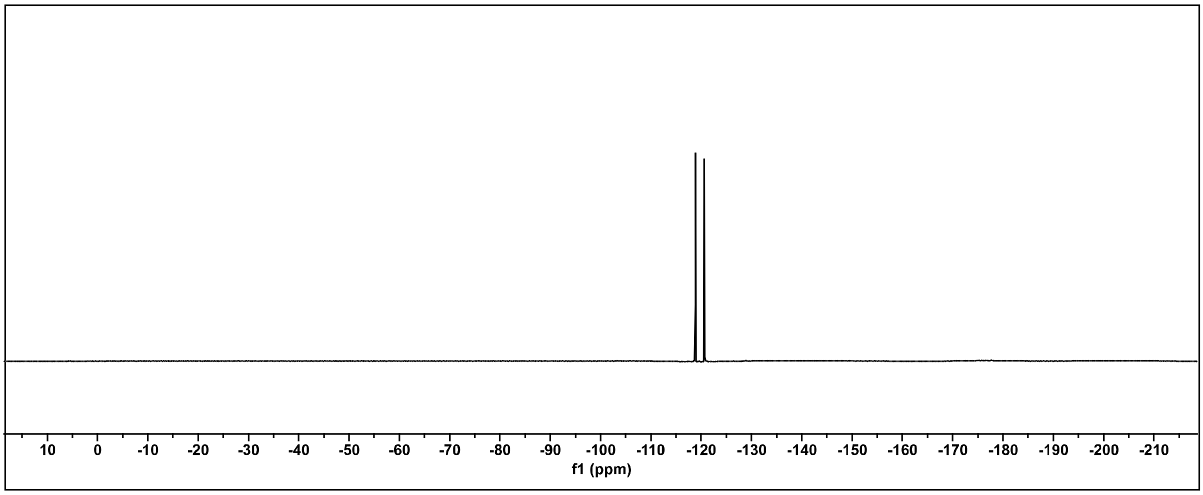

1-(8-(4-((4-Bromo -2,6- difluorophenyl) diazenyl)- 3,5-difluorophenyl) oct7-yn-1-yl)-N-(4-(1-ethylpentyl)-1,6-dihydro-6-oxo-2-pyrimidinyl))urea) 15

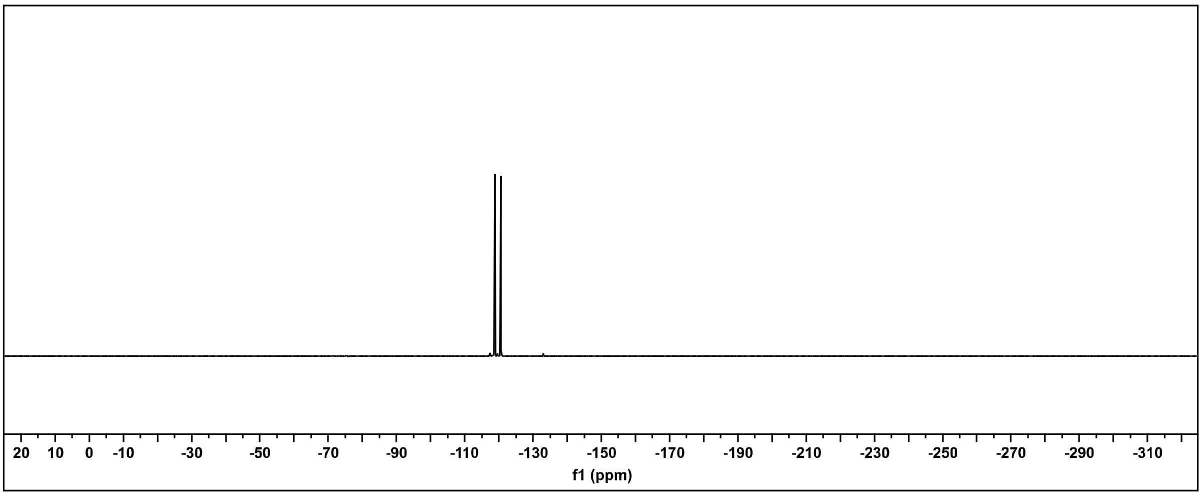

9-(4-((2,6-Difluoro-4-(8-(3-(pyridin-2-yl)ureido)oct-1-yn-1-yl)phenyl)diazenyl)-3,5-difluorophenyl)-N-(1,8- naphthyridin-2-yl)non-8-ynamide Foldamer I

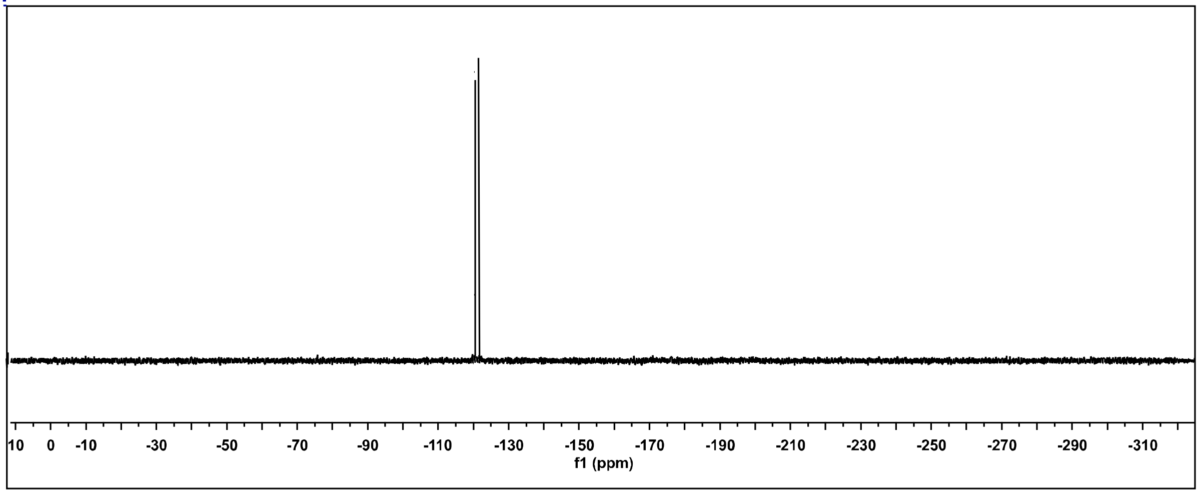

1,2-Bis(2,6-difluoro-4-1-(Oct-7-yn-1-yl)-N-(4-(1-ethylpentyl)-1,6-dihydro-6-oxo-2-pyrimidinyl))urea)diazene Foldamer II

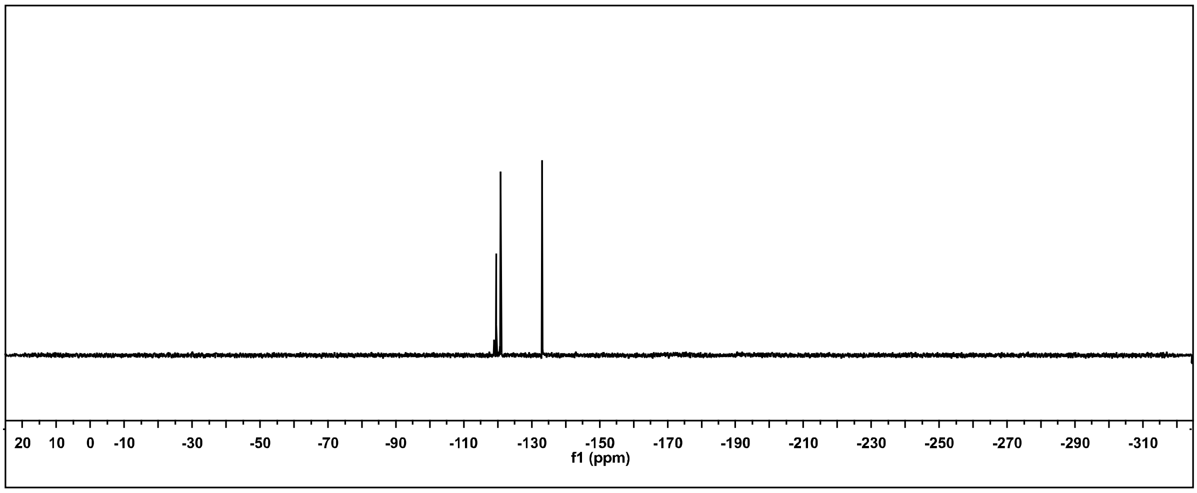

9- (4- ((2,6-Difluoro - 4 - (oct-7-yn-1-yl) -N- (4-(1-ethylpentyl) - 1,6 - dihydro-6-oxo -2-pyrimidinyl)) urea) phenyl) diazenyl) - 3,5 - difluorophenyl) -N- ((1,8-naphthyridin-2-yl)-2-ethylhexanamide)non-8-ynamide Foldamer III

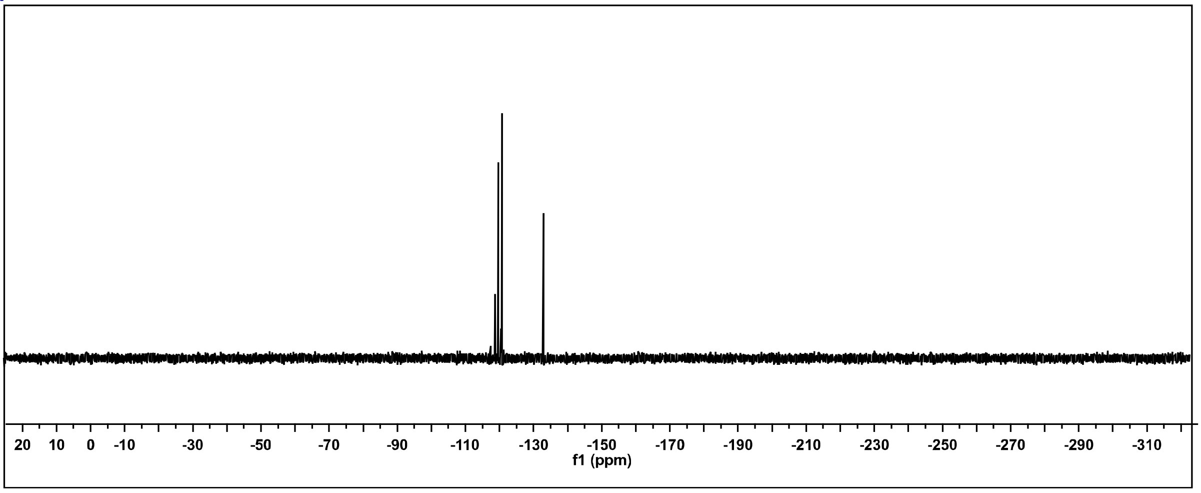

9.0 References

[1] A. Baeyer, *Berichte der deutschen chemischen Gesellschaft* **1874**, *7*, 1638-1640.

[2] C. Reichardt, W. Scheibelein, *Tetrahedron Lett.* **1977**, *18*, 2087-2090.

[3] C. R. Opie, N. Kumagai, M. Shibasaki, *Angew. Chem. Int. Ed.* **2017**, *56*, 3349-3353.

[4] G. B. W. L. Ligthart, H. Ohkawa, R. P. Sijbesma, E. W. Meijer, *J. Org. Chem.* **2006**, *71*, 375-378.

[5] H. M. Coubrough, S. C. C. van der Lubbe, K. Hetherington, A. Minard, C. Pask, M. J. Howard, C. Fonseca Guerra, A. J. Wilson, *Chem. Eur. J.* **2019**, *25*, 785-795.

[6] C. R. Opie, N. Kumagai, M. Shibasaki, *Angew. Chem., Int. Ed.* **2017**, *56*, 3349-3353.

[7] H. M. Coubrough, S. C. C. van der Lubbe, K. Hetherington, A. Minard, C. Pask, M. J. Howard, C. Fonseca Guerra, A. J. Wilson, **2019**, *25*, 785-795.

[8] C. Bannwarth, E. Caldeweyher, S. Ehlert, A. Hansen, P. Pracht, J. Seibert, S. Spicher, S. Grimme, *WIREs Computational Molecular Science* **2021**, *11*, e1493.

[9] C. Bannwarth, S. Ehlert, S. Grimme, *J. Chem. Theory Comput.* **2019**, *15*, 1652-1671.

[10] S. Ehlert, M. Stahn, S. Spicher, S. Grimme, *J. Chem. Theory Comput.* **2021**, *17*, 4250-4261.
